# Supplementary material for: Efficient access to general α-tertiary amines via water-accelerated organocatalytic multicomponent allylation
Source: Nat Commun. 2022 May 16;13:2702. doi: 10.1038/s41467-022-30281-z (PMC9110412; doi:10.1038/s41467-022-30281-z)
Supplement: Supplementary file 1 — Supplementary information [file 41467_2022_30281_MOESM1_ESM.pdf]

*Supplementary Information*

# Efficient access to general α-tertiary amines via water-accelerated organocatalytic multicomponent allylation

Prithwish Goswami[†], Sung Yeon Cho[†], Jin Hyun Park[†], Woo Hee Kim, Hyun Jin Kim, Myoung Hyeon Shin, Han Yong Bae*

Department of Chemistry, Sungkyunkwan University, Suwon, Republic of Korea, 16419
*E-mail: hybae@skku.edu

## Table of Contents


**1. General Information** ..................... **S3**

**2. Reaction Optimization** ..................... **S5**

2.1. Acid catalyst screening ..................... **S5**

2.2. Activator screening ..................... **S6**

2.3. Reaction medium screening ..................... **S6**

2.4. Allylation reagent screening ..................... **S7**

2.5. Amine source screening ..................... **S7**

**3. Synthetic Protocols** ..................... **S8**

3.1. General procedures for the allylation reactions ..................... **S8**

3.2. Procedures for the synthetic transformations ..................... **S8**

**4. Analytical Data of the Products** ..................... **S10**

**5. NMR Spectra of the Products** ..................... **S20**

**6. Computational Studies on the Mechanistic Investigation** ..................... **S67**

**7. Supplementary References** ..................... **S69**

## 1. General Information

■ **Chemical:** Chemicals were purchased from commercial vendors (*e.g.*, Aldrich, Alfa Aesar, Combi-Blocks, TCI) and used as received unless otherwise stated. Prepared β-keto esters were used after recrystallization or column chromatographic purification. α,β-Unsaturated ketones were synthesized according to the reported procedures[1,2]. Aldehydes were used after distillation. Prepared hydrazides were used after recrystallization. Squaramide-type catalysts were synthesized according to the reported procedures[3–5]. HPLC grade solvents, anhydrous solvents, NMR solvents, and additional organic solvents were purchased from commercial vendors (*e.g.*, Aldrich, Alfa Aesar, CIL Inc., Merck, Wako) and used without further distillation or purification.

■ **Reaction:** Reaction mixtures were stirred magnetically in flame-dried glassware (*e.g.*, round-bottom flask (RBF), vial, Schlenk tube) under argon gas atmosphere associating standard Schlenk technique and the room temperature was maintained at 24 ± 1 °C consistently unless otherwise specified. Chemical yields refer to spectroscopically pure products unless otherwise stated.

■ **Thin-Layer Chromatography (TLC):** TLC results were monitored using silica gel plates (Merck, Kieselgel 60 F254 0.25 mm). Visualization by staining methods (*e.g.*, $KMnO_4$ upon heating) was performed when if it was needed.

■ **Column chromatographic purification:** Purification processes were performed by using silica gel (Merck, 60 Å, 230–400 mesh, 0.040–0.063 mm).

■ **Nuclear Magnetic Resonance (NMR) spectroscopy:** $^{1}H$ NMR (500 MHz) and $^{13}C$ NMR (125.7 MHz) spectra were recorded using Bruker Ascend™ 500 spectrometer. Chemical shifts (δ) were reported by using tetramethylsilane (TMS, $Me_4Si$) as the internal standard for $^{1}H$ and $^{13}C$ NMR. Integration data were represented as follows; coupling constant ($J$ = Hz), multiplicity (s = singlet, d = doublet, t = triplet, q = quartet, m = multiplet). All analytical data and NMR spectra of known compounds are in accordance with reported literatures.

■ **High-Performance Liquid Chromatography (HPLC):** HPLC analyses were performed on YL9100 Plus$^{+}$ HPLC System instruments equipped with an isostatic pump using chiral column (CHIRALPAK® IA; 250 x 4.6 mm; particle size 5 μm) for the determination of the enantiomeric excess (*ee*) of the products.

■ **Mass Spectroscopy (MS):** High-resolution mass spectra were analyzed in electrospray ionization (HR-ESI-MS) mode by using Waters SYNAPT G2, or in fast atom bombardment (HR-FAB-MS) option by using JMS-700.

■ **Melting point:** Melting points were measured by using Büchi® B-540.

■ **Abbreviation:**

(*alphabetical order*)

allyl-Bpin; allyl-boronic acid pinacol ester

anhyd.; anhydrous

aq.; aqueous

Ar; argon

$Boc_2O$; di-tert-butyl dicarbonate

Brine; sat. NaCl aq. soln.
Bz; benzoyl
cal; calorie
calcd.; calculated
catal.; catalyst
conv.; conversion
DBSA; 4-dodecylbenzenesulfonic acid
DCM; dichloromethane
DIPEA; diisopropylethylamine (Hünig base)
DMAP; 4-(dimethylamino)pyridine
DW; deionized water
E; energy
equiv.; equivalent
ESI; electrospray ionization
Et; ethyl
FAB; fast atom bombardment
h; hours
HPLC; high-performance liquid chromatography
J; Joule
M; molar concentration (molarity)
Me; methyl
mol; mole
mp; melting point
MS; mass spectroscopy
*m/z*; mass-to-charge ratio
NBS; N-bromosuccinimide
NMR; nuclear magnetic resonance
Ph; phenyl
*p*TsOH; *p*-toluenesulfonic acid
RBF; round-bottom flask
$R_f$; retardation (retention) factor
rpm; revolutions per minute
rt; room temperature
sat.; saturated
soln.; solution
THF; tetrahydrofuran
TLC; thin-layer chromatography
v/v; volume per volume

# 2. Reaction Optimization

## 2.1. Supplementary Table 1. Acid catalyst screening

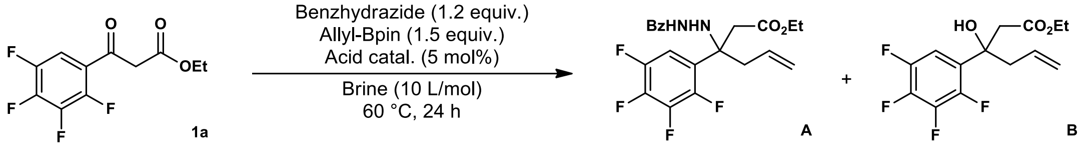


| Entry | Acid catal. | Conv. (%, [A/B]) |
|---|---|---|
| 1 | - | n.d. : 22 |
| 2 | BzOH | < 5 : 24 |
| 3 | Erucic acid | n.d. : 32 |
| 4 | (±)-PA | 46 : 15 |
| 5 | (*S*)-BINOL | n.d. : 30 |
| 6 | STDS | 67 : 24[e] |
| 7 | 4-SC[4]A | 14 : 34[e] |
| 8 | Nafion™ NR50 | 27 : 25 |
| 9 | $Tf_2NH$ | 63 : 20 |
| 10 | TfOH | 9 : 22 |
| 11 | *p*TsOH | 3 : 25 |
| 12 | PFBS | 43 : 23 |
| 13 | DBSA | 62 : 12 |
| 14 | DBSA | 78 : 8[f] |
| 15 | DBSA | 63 : 11[g] |

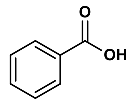

BzOH

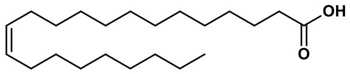

Erucic acid

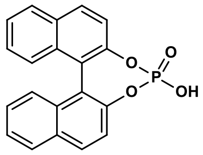

(±)-PA

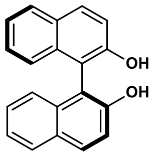

(*S*)-BINOL

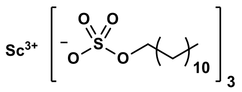

STDS

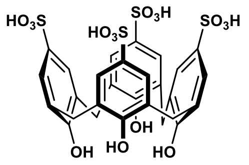

4-SC[4]A

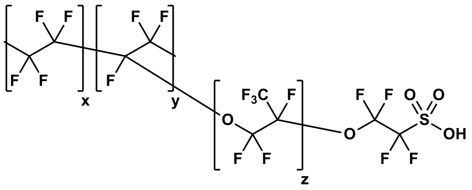

Nafion™ NR50

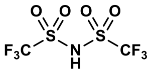

$Tf_2NH$

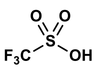

TfOH

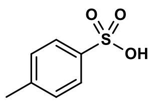

*p*TsOH

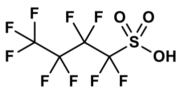

PFBS

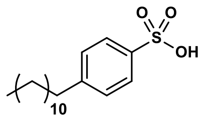

DBSA

[a]Reaction condition: **1a** (0.2 mmol), benzhydrazide (1.2 equiv., 0.24 mmol), allyl-Bpin (1.5 equiv., 0.3 mmol), acid catal. (5 mol%, 0.01 mmol), brine (10 L/mol, 2 mL), 60 °C, 24 h. [b]Conversion (conv.) was determined by $^1$H NMR integration (internal standard: 1,3,5-trimethoxybenzene). [c]n.d. = not detected. [d]Abbreviation: PFBS; perfluorobutanesulfonic acid, $Tf_2NH$; trifluoromethanesulfonimide, TfOH; trifluoromethanesulfonic acid. [e]complex mixture & partial decomposition. [f]DBSA (10 mol%) was used as catalyst. [g]Variation: rt, 72 h.

### 2.2. Supplementary Table 2. Activator screening

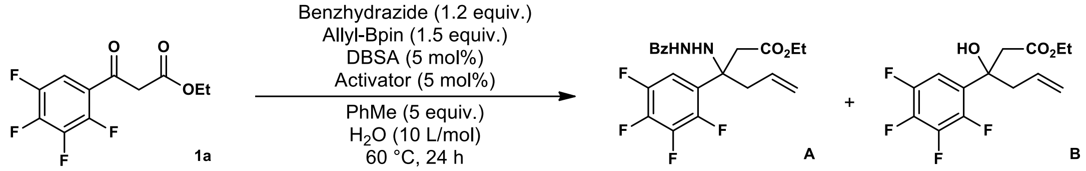


| Entry | Activator | Conv. (%, [A/B]) |
|---|---|---|
| 1 | SQA-III | 1 : 8[c] |
| 2 | SQA-III | 88 : 4[d] |
| 3 | SQA-III | 96 : 4 |
| 4 | SQA-I | 83 : 2 |
| 5 | SQA-II | 17 : 6 |

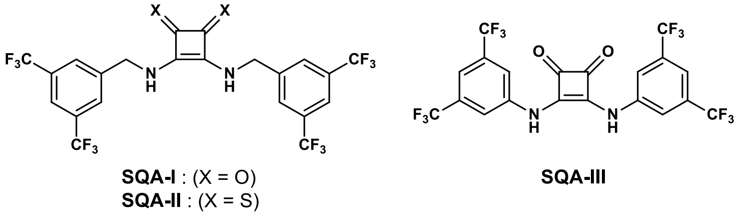


[a]Reaction condition: **1a** (0.2 mmol), benzhydrazide (1.2 equiv., 0.24 mmol), allyl-Bpin (1.5 equiv., 0.3 mmol), DBSA (5 mol%, 0.01 mmol), activator (5 mol%, 0.01 mmol), PhMe (5 equiv., 1 mmol), $H_2O$ (10 L/mol, 2 mL), 60 °C, 24 h. [b]Conversion (conv.) was determined by $^1$H NMR integration (internal standard: 1,3,5-trimethoxybenzene). [c]No (w/o) DBSA (5 mol%, 0.01 mmol). [d]No (w/o) PhMe (5 equiv., 1 mmol).

### 2.3. Supplementary Table 3. Reaction medium screening

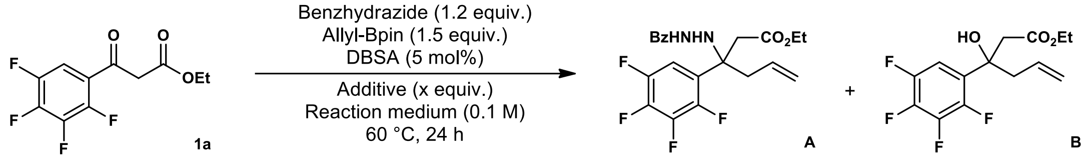


| Entry | Additive (x) | Reaction medium | Conv. (%, [A/B]) |
|---|---|---|---|
| 1 | - | PhMe | 45 : n.d. |
| 2 | $H_2O$ (5) | PhMe | 33 : 3 |
| 3 | - | $Et_2O$ | 19 : 5 |
| 4 | - | THF | 22 : 2 |
| 5 | - | DCM | 21 : 1 |
| 6 | - | MeCN | 24 : 1 |
| 7 | - | ChCl/glycerol = 1:2[d] | 33 : 8 |
| 8 | - | ChCl/urea = 1:2[d] | < 1 : 15 |
| 9 | - | SPGS-550-M (2 wt. %) / $H_2O$[d] | 11 : 1 |
| 10 | - | TPGS (2 wt. %) / $H_2O$[d] | 15 : 1 |
| 11 | - | $H_2O$[d] | 77 : 3 |
| 12 | - | Brine[d] | 62 : 10[f] |
| 13 | - | Sat. $LiClO_4$ aq. soln.[d] | 46 : 11 |

[a]Reaction condition: **1a** (0.2 mmol), benzhydrazide (1.2 equiv., 0.24 mmol), allyl-Bpin (1.5 equiv., 0.3 mmol), DBSA (5 mol%, 0.01 mmol), additive (x equiv., 0.2*x mmol), reaction medium (0.1 M, 2 mL), 60 °C, 24 h. [b]Conversion (conv.) was determined by $^1$H NMR integration (internal standard: 1,3,5-trimethoxybenzene). [c]Abbreviation: ChCl; choline chloride. [d]Reaction medium (10 L/mol). [e]n.d. = not

detected. [f]No (w/o) stir.

### 2.4. Supplementary Figure 1. Allylation reagent screening

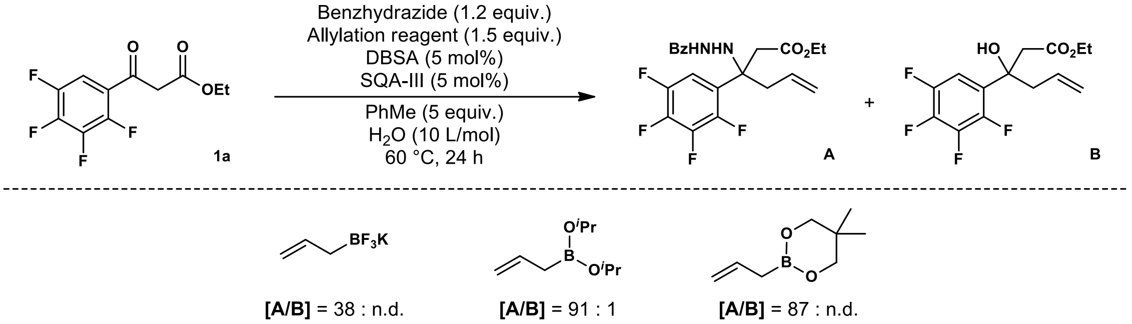


[a]Reaction condition: **1a** (0.2 mmol), benzhydrazide (1.2 equiv., 0.24 mmol), allylation reagent (1.5 equiv., 0.3 mmol), DBSA (5 mol%, 0.01 mmol), SQA-III (5 mol%, 0.01 mmol), PhMe (5 equiv., 1 mmol), $H_2O$ (10 L/mol, 2 mL), 60 °C, 24 h. [b]Conversion (conv.) was determined by $^1$H NMR integration (internal standard: 1,3,5-trimethoxybenzene). [c]Abbreviation: $^i$Pr; iso-propyl. [d]n.d. = not detected.

### 2.5. Supplementary Figure 2. Amine source screening

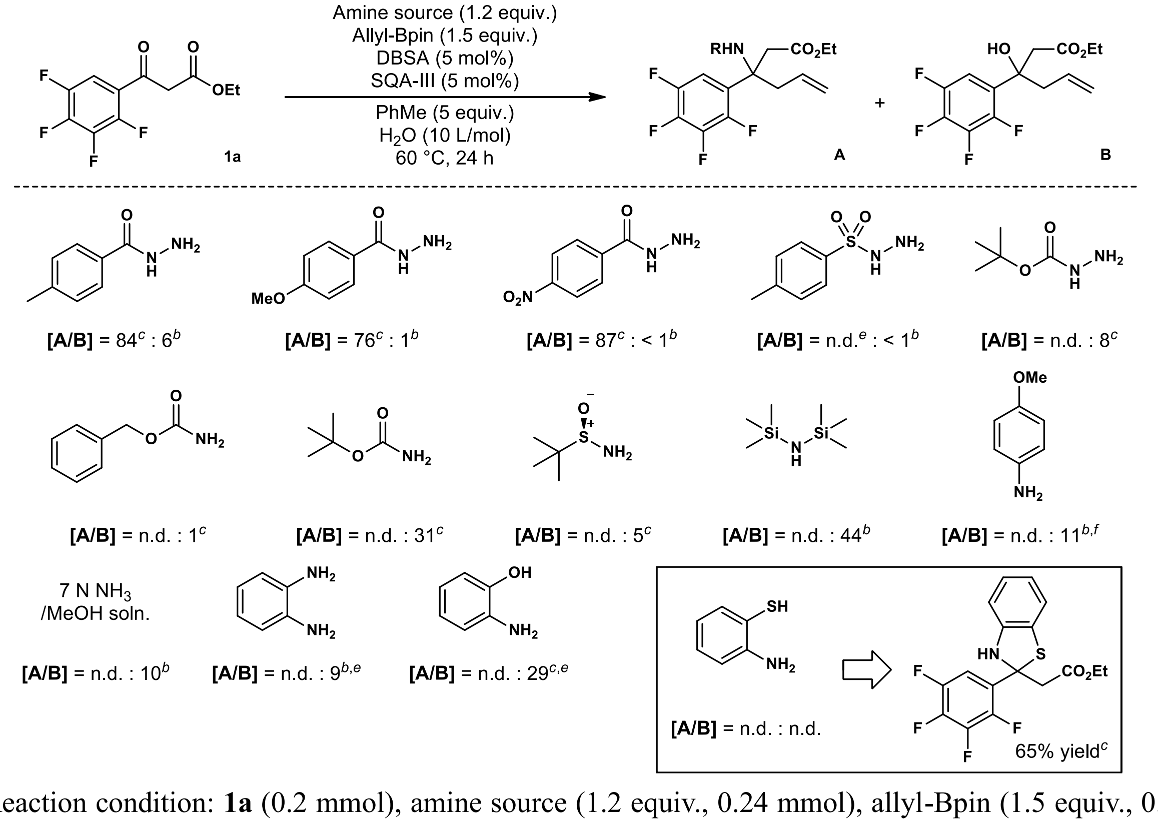


[a]Reaction condition: **1a** (0.2 mmol), amine source (1.2 equiv., 0.24 mmol), allyl-Bpin (1.5 equiv., 0.3 mmol), DBSA (5 mol%, 0.01 mmol), SQA-III (5 mol%, 0.01 mmol), PhMe (5 equiv., 1 mmol), $H_2O$ (10 L/mol, 2 mL), 60 °C, 24 h. [b]Conversion (conv.) was determined by $^1$H NMR integration (internal standard: 1,3,5-trimethoxybenzene). [c]Isolated yield. [d]n.d. = not detected. [e]partial decomposition. [f]complex mixture.

# 3. Synthetic Protocols

**3.1. Supplementary Methods 1.** General procedures for the allylation reactions

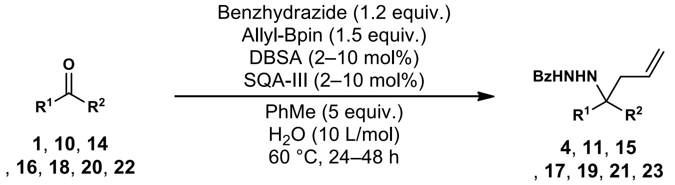


In a flame-dried capped vial, equipped with multiple magnetic stirring bar and filled with Ar gas, **1**, **10**, **14**, **16**, **18**, **20**, **22** (0.2 mmol), benzhydrazide (1.2 equiv.), SQA-III (2 – 10 mol%), and DBSA (2 – 10 mol%) were added. Subsequently, allyl-Bpin (1.5 equiv.), PhMe (5 equiv.), and $H_2O$ (deionized, 10 L/mol) were added to the reaction mixture then sealed to stir vigorously (rpm > 1000) at rt or 60 °C for 24 – 48 h. The resulting mixture was diluted and extracted with EtOAc/brine, and the combined organic layer was dried over anhyd. $Na_2SO_4$, and filtered. The filtrate was concentrated *in vacuo*, and the residue was purified by column chromatography on silica gel (EtOAc:hexanes = 1:9 to 1:4 v/v) to afford corresponding allylation-reaction product (**4**, **11**, **15**, **17**, **19**, **21**, **23**).

*** Typical scale-up experiment (on 1 g scale)**

In a flame-dried capped RBF, equipped with a magnetic stirring bar and filled with Ar gas, **1a**, **1b**, **10a**, **10c**, **14d** (1 g), benzhydrazide (1.2 equiv.), SQA-III (0.5 – 2.5 mol%), and DBSA (0.5 – 2.5 mol%) were added. Subsequently, allyl-Bpin (1.5 equiv.), PhMe (5 equiv.), and $H_2O$ (deionized, 10 L/mol) were added to the reaction mixture then sealed to stir vigorously (rpm > 1000) at rt or 60 °C for 48 h. The resulting mixture was diluted and extracted with EtOAc/brine, and the combined organic layer was dried over anhyd. $Na_2SO_4$, and filtered. The filtrate was concentrated *in vacuo*, and the residue was purified by column chromatography on silica gel (EtOAc:hexanes = 1:9 to 1:4 v/v) to afford the corresponding allylation-reaction product (**4a** (1.46 g, 91% yield), **4b** (2.20 g, 99% yield), **11a** (3.09 g, 96% yield), **11c** (2.45 g, 94% yield), **15d** (1.97 g, 90% yield)).

**3.2. Supplementary Methods 2.** Procedures for the synthetic transformations

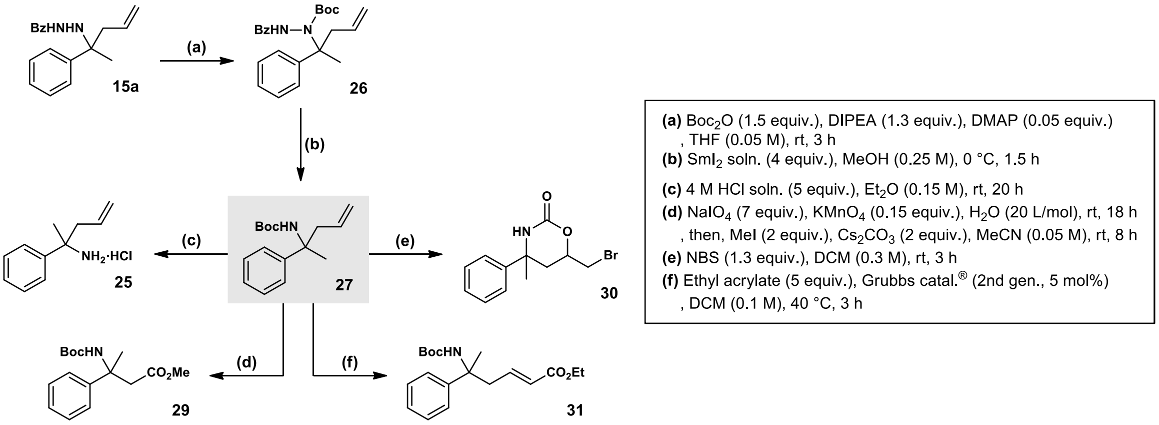


In a flame-dried capped RBF, equipped with a magnetic stirring bar and filled with Ar gas, allylation-reaction product (**15a**, 3 mmol) was dissolved in THF (0.1 M, 30 mL). After addition of DIPEA (1.3 equiv., 3.9 mmol) and cooled down to 0 °C, $Boc_2O$ (1.5 equiv., 4.5 mmol) dissolved in THF (0.1 M, 30 mL) was added to the reaction mixture dropwise. Subsequently, DMAP (0.05 equiv., 0.15 mmol) was added to the reaction mixture then sealed to stir at rt for 3 h. The crude mixture was quenched with DW and extracted with EtOAc, and the combined organic layer was dried over anhyd. $Na_2SO_4$, and filtered. The filtrate was concentrated *in vacuo*, and the residue was purified by column chromatography on

silica gel (EtOAc:hexanes = 1:9 to 1:4 v/v) to afford corresponding product (**26**).

In a flame-dried capped RBF, equipped with a magnetic stirring bar and filled with Ar gas, **26** (1.5 mmol) was dissolved in MeOH (anhyd., 0.25 M, 6 mL). After cooling down to 0 °C, $SmI_2$ (0.1 M THF soln., 4 equiv., 6 mmol) was added to the reaction mixture dropwise then sealed to stir at 0 °C for 1.5 h. The crude mixture was quenched with DW and extracted with EtOAc, and the combined organic layer was dried over anhyd. $Na_2SO_4$, and filtered. The filtrate was concentrated *in vacuo*, and the residue was purified by column chromatography on silica gel (EtOAc:hexanes = 1:20 to 1:9 v/v) to afford corresponding product (**27**).

In a flame-dried capped vial, equipped with a magnetic stirring bar and filled with Ar gas, **27** (0.1 mmol) was dissolved in $Et_2O$ (anhyd., 0.15 M, 0.67 mL) and cooled down to 0 °C. Subsequently, 4 M HCl/1,4-dioxane soln. (5 equiv., 0.5 mmol) was added to the reaction mixture then sealed to stir at rt for 20 h. The crude mixture was concentrated *in vacuo*, and the residue was washed with $Et_2O$ (0.15 M, 0.67 mL) to afford the corresponding product (**25**).

In a flame-dried capped vial, equipped with a magnetic stirring bar and filled with Ar gas, $KMnO_4$ (0.15 equiv., 0.015 mmol) and $NaIO_4$ (7 equiv., 0.7 mmol) were dissolved in $H_2O$ (deionized, 20 L/mol, 2 mL). Subsequently, **27** (0.1 mmol) was added to the reaction mixture then sealed to stir at rt for 18 h. The crude mixture was extracted with $Et_2O$, and the combined organic layer was dried over anhyd. $Na_2SO_4$, and filtered. The filtrate was concentrated *in vacuo*, and the residue was directly used in next step without further purification. The dried resulting mixture was dissolved in MeCN (anhyd., 0.05 M, 2 mL). Subsequently, $Cs_2CO_3$ (2 equiv., 0.2 mmol) and MeI (2 equiv., 0.2 mmol) were added to the reaction mixture then sealed to stir at rt for 8 h. The crude mixture was quenched with DW and extracted with EtOAc, and the combined organic layer was dried over anhyd. $Na_2SO_4$, and filtered. The filtrate was concentrated *in vacuo*, and the residue was purified by column chromatography on silica gel (EtOAc:hexanes = 1:9 to 1:4 v/v) to afford corresponding product (**29**).

In a flame-dried capped vial, equipped with a magnetic stirring bar and filled with Ar gas, **27** (0.1 mmol) was dissolved in DCM (anhyd., 0.3 M, 0.33 mL). Subsequently, NBS (1.3 equiv., 0.13 mmol) was added to the reaction mixture then sealed to stir at rt for 3 h. The crude mixture was concentrated *in vacuo*, then $Et_2O$ (0.3 M, 0.33 mL) and 10% NaOH aq. soln. (0.7 M, 0.14 mL) were added to the resulting mixture. After stir at rt for 0.5 h, the crude mixture was extracted with DCM, and the combined organic layer was dried over anhyd. $Na_2SO_4$, and filtered. The filtrate was concentrated *in vacuo*, and the residue was purified by short-path column chromatography on silica gel (EtOAc:hexanes = 1:2 v/v then DCM:MeOH = 2:1 v/v) to afford the corresponding product (**30**).

In a flame-dried capped Schlenk tube, equipped with a magnetic stirring bar and filled with Ar gas, **27** (0.1 mmol) and ethyl acrylate (5 equiv., 0.5 mmol) were dissolved in DCM (anhyd., 0.1 M. 1 mL). After addition of the Grubbs catalyst® (2nd generation, 5 mol%, 0.005 mmol), the reaction mixture was sealed to stir at 40 °C for 3 h. The crude mixture was concentrated *in vacuo*, and the residue was purified by column chromatography on silica gel (EtOAc:hexanes = 1:20 to 1:4 v/v) to afford corresponding product (**31**).

## 4. Analytical Data of the Products

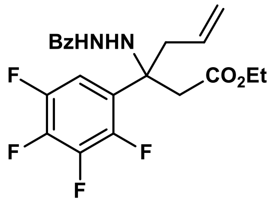


**4a**, *new compound*, yellow waxy oil, 79.6 mg, 93% yield.
**$R_f$** = 0.35 (EtOAc:hexanes = 1:4 v/v).
**$^1$H NMR** (500 MHz, $CDCl_3$, $Me_4Si$): δ 8.11 (d, *J* = 7.4 Hz, 1H), 7.77 (d, *J* = 7.5 Hz, 2H), 7.53 (t, *J* = 7.4 Hz, 1H), 7.49 – 7.37 (m, 3H), 5.91 (d, *J* = 7.6 Hz, 1H), 5.72 – 5.57 (m, 1H), 5.25 – 5.05 (m, 2H), 4.13 – 4.00 (m, 2H), 3.11 (d, *J* = 15.4 Hz, 1H), 2.97 (d, *J* = 15.3 Hz, 1H), 2.77 – 2.61 (m, 2H), 1.13 (t, *J* = 7.1 Hz, 3H).
**$^{13}$C NMR** (126 MHz, $CDCl_3$, $Me_4Si$): δ 171.31, 166.93, 146.30 (dd, $^1J_{C\text{-}F}$ = 245.2, 9.2 Hz), 145.16 (ddd, $^1J_{C\text{-}F}$ = 246.4, 11.2, 3.0 Hz), 140.72 (dddd, $^1J_{C\text{-}F}$ = 252.1, 16.4, 11.9, 3.6 Hz), 139.15 (ddd, $^1J_{C\text{-}F}$ = 253.4, 13.4, 2.1 Hz), 132.19, 132.13, 131.89, 128.81, 126.88, 126.50 (dt, $^2J_{C\text{-}F}$ = 9.6, 4.7 Hz), 120.03, 110.18 (dt, $^2J_{C\text{-}F}$ = 21.6, 3.7 Hz), 63.17 (d, $^3J_{C\text{-}F}$ = 3.2 Hz), 61.00, 41.83 (d, $^4J_{C\text{-}F}$ = 3.1 Hz), 41.59 (d, $^4J_{C\text{-}F}$ = 5.3 Hz), 14.00.
**HR-MS**: *m/z* calcd. $[C_{21}H_{20}F_4N_2O_3 + H]^+$: 425.1483; found (FAB): 425.1485.

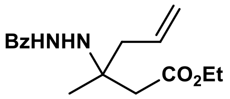


**4b**, *new compound*, yellow oil, 57.4 mg, > 99% yield.
**$R_f$** = 0.17 (EtOAc:hexanes = 1:4 v/v).
**$^1$H NMR** (500 MHz, $CDCl_3$, $Me_4Si$): δ 8.17 (s, 1H), 7.86 – 7.77 (m, 2H), 7.51 (t, *J* = 7.4 Hz, 1H), 7.44 (t, *J* = 7.5 Hz, 2H), 5.96 – 5.83 (m, 1H), 5.59 – 5.02 (m, 3H), 4.20 (q, *J* = 7.1 Hz, 2H), 2.51 (d, *J* = 13.9 Hz, 1H), 2.46 (d, *J* = 13.9 Hz, 1H), 2.37 (dd, *J* = 13.7, 7.7 Hz, 1H), 2.31 (dd, *J* = 13.7, 7.3 Hz, 1H), 1.29 (t, *J* = 7.1 Hz, 3H), 1.21 (s, 3H).
**$^{13}$C NMR** (126 MHz, $CDCl_3$, $Me_4Si$): δ 171.18, 165.66, 132.28, 131.65, 130.77, 127.68, 125.81, 118.12, 59.74, 58.40, 42.17, 42.10, 22.08, 13.20.
**HR-MS**: *m/z* calcd. $[C_{16}H_{22}N_2O_3 + H]^+$: 291.1703; found (FAB): 291.1706.

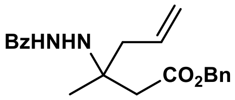


**4c**, *new compound*, yellow oil, 64.2 mg, 91% yield.
**$R_f$** = 0.29 (EtOAc:hexanes = 1:4 v/v).
**$^1$H NMR** (500 MHz, $CDCl_3$, $Me_4Si$): δ 7.94 (s, 1H), 7.69 (d, *J* = 7.3 Hz, 2H), 7.50 (t, *J* = 7.4 Hz, 1H), 7.45 – 7.28 (m, 7H), 5.96 – 5.82 (m, 1H), 5.30 – 5.03 (m, 5H), 2.55 (d, *J* = 13.7 Hz, 1H), 2.50 (d, *J* = 13.7 Hz, 1H), 2.36 (dd, *J* = 13.8, 7.8 Hz, 1H), 2.31 (dd, *J* = 13.8, 7.3 Hz, 1H), 1.21 (s, 3H).
**$^{13}$C NMR** (126 MHz, $CDCl_3$, $Me_4Si$): δ 171.79, 166.64, 135.58, 133.22, 132.61, 131.73, 128.66, 128.48, 128.45, 126.77, 119.22, 66.74, 59.61, 43.22, 43.11, 23.16.
**HR-MS**: *m/z* calcd. $[C_{21}H_{24}N_2O_3 + H]^+$: 353.1860; found (FAB): 353.1863.

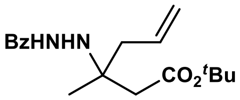


**4d**, *new compound*, yellow oil, 53.8 mg, 84% yield.
**$R_f$** = 0.36 (EtOAc:hexanes = 1:4 v/v).
**$^1$H NMR** (500 MHz, $CDCl_3$, $Me_4Si$): δ 8.16 (s, 1H), 7.85 – 7.73 (m, 2H), 7.51 (t, *J* = 7.4 Hz, 1H), 7.44 (t, *J* = 7.5 Hz, 2H), 5.97 – 5.83 (m, 1H), 5.52 – 5.02 (m, 3H), 2.46 – 2.33 (m, 3H), 2.29 (dd, *J* = 13.7, 7.3 Hz, 1H), 1.49 (s, 9H), 1.20 (s, 3H).
**$^{13}$C NMR** (126 MHz, $CDCl_3$, $Me_4Si$): δ 171.71, 166.52, 133.58, 132.89, 131.80, 128.78, 126.92, 119.10, 81.43, 59.64, 44.46, 43.32, 28.26, 23.23.
**HR-MS**: *m/z* calcd. $[C_{18}H_{26}N_2O_3 + Na]^+$: 341.1836; found (ESI): 341.1841.

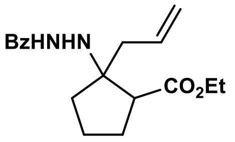

**4e**, *new compound*, yellow oil, 40.3 mg, 63% yield.
**$R_f$** = 0.40 (EtOAc:hexanes = 1:4 v/v).
**$^1$H NMR** (500 MHz, $CDCl_3$, $Me_4Si$): δ 8.27 (d, *J* = 6.1 Hz, 1H), 7.83 – 7.75 (m, 2H), 7.58 – 7.48 (m, 1H), 7.45 (t, *J* = 7.5 Hz, 2H), 6.03 – 5.86 (m, 1H), 5.28 (d, *J* = 7.6 Hz, 1H), 5.24 – 5.15 (m, 2H), 4.23 (dddd, *J* = 10.2, 10.2, 7.1, 7.1 Hz, 2H), 2.74 (dd, *J* = 10.3, 7.7 Hz, 1H), 2.53 (dd, *J* = 13.8, 7.5 Hz, 1H), 2.41 (dd, *J* = 13.8, 7.3 Hz, 1H), 2.27 – 2.10 (m, 1H), 2.01 – 1.70 (m, 4H), 1.60 – 1.47 (m, 1H), 1.31 (t, *J* = 7.1 Hz, 3H).
**$^{13}$C NMR** (126 MHz, $CDCl_3$, $Me_4Si$): δ 175.57, 165.74, 133.58, 132.60, 131.68, 128.70, 126.72, 119.39, 71.21, 60.89, 53.17, 42.36, 33.78, 29.02, 23.33, 14.28.
**HR-MS**: *m/z* calcd. $[C_{18}H_{24}N_2O_3 + H]^+$: 317.1860; found (FAB): 317.1869.

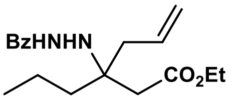

**4f**, *new compound*, yellow oil, 58.5 mg, 91% yield.
**$R_f$** = 0.42 (EtOAc:hexanes = 1:4 v/v).
**$^1$H NMR** (500 MHz, $CDCl_3$, $Me_4Si$): δ 8.22 (s, 1H), 7.85 – 7.77 (m, 2H), 7.51 (t, *J* = 7.3 Hz, 1H), 7.44 (t, *J* = 7.5 Hz, 2H), 5.97 – 5.83 (m, 1H), 5.33 (s, 1H), 5.22 – 5.12 (m, 2H), 4.20 (q, *J* = 7.1 Hz, 2H), 2.52 (d, *J* = 13.8 Hz, 1H), 2.48 (d, *J* = 13.8 Hz, 1H), 2.40 – 2.25 (m, 2H), 1.57 – 1.34 (m, 4H), 1.29 (t, *J* = 7.1 Hz, 3H), 0.92 (t, *J* = 6.8 Hz, 3H).
**$^{13}$C NMR** (126 MHz, $CDCl_3$, $Me_4Si$): δ 172.63, 166.20, 133.31, 132.86, 131.76, 128.77, 126.87, 119.07, 61.83, 60.91, 42.03, 40.05, 37.83, 16.71, 14.71, 14.31.
**HR-MS**: *m/z* calcd. $[C_{18}H_{26}N_2O_3 + H]^+$: 319.2016; found (FAB): 319.2019.

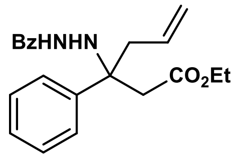

**4g**, *new compound*, yellow waxy solid, 61.0 mg, 86% yield.
**$R_f$** = 0.32 (EtOAc:hexanes = 1:4 v/v).
**$^1$H NMR** (500 MHz, $CDCl_3$, $Me_4Si$): δ 8.12 (s, 1H), 7.65 (d, *J* = 7.3 Hz, 2H), 7.54 – 7.43 (m, 3H), 7.41 – 7.30 (m, 4H), 7.28 – 7.25 (m, 1H), 6.04 (s, 1H), 5.67 – 5.53 (m, 1H), 5.20 – 5.02 (m, 2H), 4.18 – 4.02 (m, 2H), 3.02 (d, *J* = 15.2 Hz, 1H), 2.98 (d, *J* = 15.2 Hz, 1H), 2.78 – 2.67 (m, 2H), 1.18 (t, *J* = 7.1 Hz, 3H).
**$^{13}$C NMR** (126 MHz, $CDCl_3$, $Me_4Si$): δ 171.94, 165.84, 142.13, 133.08, 132.93, 131.65, 128.69, 128.49, 127.34, 126.83, 126.37, 119.37, 64.21, 60.88, 43.78, 42.12, 14.14.
**HR-MS**: *m/z* calcd. $[C_{21}H_{24}N_2O_3 + H]^+$: 353.1860; found (FAB): 353.1861.

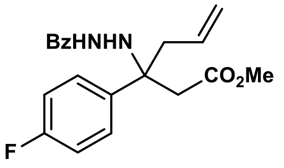

**4h**, *new compound*, yellow oil, 50.6 mg, 71% yield.
**$R_f$** = 0.24 (EtOAc:hexanes = 1:4 v/v).
**$^1$H NMR** (500 MHz, $CDCl_3$, $Me_4Si$): δ 7.98 (s, 1H), 7.72 – 7.61 (m, 2H), 7.54 – 7.43 (m, 3H), 7.39 (t, *J* = 7.6 Hz, 2H), 7.03 (t, *J* = 8.6 Hz, 2H), 5.96 (s, 1H), 5.67 – 5.51 (m, 1H), 5.22 – 5.00 (m, 2H), 3.65 (s, 3H), 3.00 (d, *J* = 15.3 Hz, 1H), 2.97 (d, *J* = 15.4 Hz, 1H), 2.78 – 2.62 (m, 2H).
**$^{13}$C NMR** (126 MHz, $CDCl_3$, $Me_4Si$): δ 172.21, 166.25, 161.97 (d, $^1J_{C\text{-}F}$ = 246.4 Hz), 137.95 (d, $^4J_{C\text{-}F}$ = 3.1 Hz), 132.82, 131.85, 128.79, 128.19 (d, $^3J_{C\text{-}F}$ = 7.9 Hz), 126.86, 119.63, 115.35 (d, $^2J_{C\text{-}F}$ = 21.2 Hz), 63.92, 52.03, 43.86, 41.79.
**HR-MS**: *m/z* calcd. $[C_{20}H_{21}FN_2O_3 + H]^+$: 357.1609; found (FAB): 357.1613.

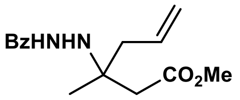

**4i**, *new compound*, yellow oil, 50.3 mg, 91% yield.
**$R_f$** = 0.17 (EtOAc:hexanes = 1:4 v/v).
**$^1$H NMR** (500 MHz, $CDCl_3$, $Me_4Si$): δ 8.10 (s, 1H), 7.86 – 7.75 (m, 2H), 7.55 – 7.48 (m, 1H), 7.48 – 7.39 (m, 2H), 5.96 – 5.82 (m, 1H), 5.59 – 5.03 (m, 3H), 3.73 (s, 3H), 2.50 (q, *J* = 14.0 Hz, 2H), 2.34 (qd, *J* = 13.8, 7.5 Hz, 2H), 1.21 (S, 3H).

**$^{13}$C NMR** (126 MHz, $CDCl_3$, $Me_4Si$): δ 172.69, 166.89, 133.34, 132.73, 131.91, 128.81, 126.94, 119.26, 59.47, 51.91, 43.25, 42.97, 23.18.
**HR-MS**: *m/z* calcd. $[C_{15}H_{20}N_2O_3 + Na]^+$: 299.1366; found (ESI): 299.1373.

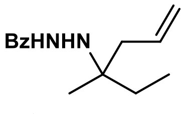


**11a**, *known compound*[6], 44.6 mg, 96% yield.
**$^1$H NMR** (500 MHz, $CDCl_3$, $Me_4Si$): δ 7.87 – 7.69 (m, 2H), 7.68 – 7.46 (m, 2H), 7.42 (t, *J* = 7.5 Hz, 2H), 6.02 – 5.86 (m, 1H), 5.24 – 4.41 (m, 3H), 2.27 – 2.17 (m, 2H), 1.57 – 1.40 (m, 2H), 1.08 (s, 3H), 0.94 (t, *J* = 7.5 Hz, 3H).

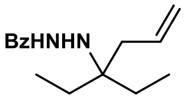


**11b**, *new compound*, yellow oil, 45.3 mg, 92% yield.
**$R_f$** = 0.36 (EtOAc:hexanes = 1:4 v/v).
**$^1$H NMR** (500 MHz, $CDCl_3$, $Me_4Si$): δ 7.72 (d, *J* = 7.6 Hz, 2H), 7.49 (t, *J* = 7.3 Hz, 1H), 7.42 (t, *J* = 7.3 Hz, 2H), 7.36 (s, 1H), 6.08 – 5.91 (m, 1H), 5.31 – 4.66 (m, 3H), 2.21 (d, *J* = 7.3 Hz, 2H), 1.47 (q, *J* = 7.5 Hz, 4H), 0.93 (t, *J* = 7.5 Hz, 6H).
**$^{13}$C NMR** (126 MHz, $CDCl_3$, $Me_4Si$): δ 166.76, 134.95, 133.11, 131.71, 128.74, 126.86, 117.49, 61.87, 39.69, 26.98, 7.76.
**HR-MS**: *m/z* calcd. $[C_{15}H_{22}N_2O + Na]^+$: 269.1624; found (ESI): 269.1630.

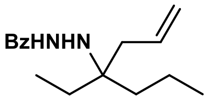


**11c**, *new compound*, yellow oil, 49.4 mg, 95% yield.
**$R_f$** = 0.41 (EtOAc:hexanes = 1:4 v/v).
**$^1$H NMR** (500 MHz, $CDCl_3$, $Me_4Si$): δ 7.72 (d, *J* = 7.5 Hz, 2H), 7.49 (t, *J* = 7.3 Hz, 1H), 7.42 (t, *J* = 7.5 HZ, 2H), 7.34 (s, 1H), 6.09 – 5.89 (m, 1H), 5.33 – 4.82 (m, 3H), 2.22 (d, *J* = 7.2 Hz, 2H), 1.47 (dd, *J* = 14.9, 7.4 Hz, 2H), 1.39 (s, 4H), 1.02 – 0.87 (m, 6H).
**$^{13}$C NMR** (126 MHz, $CDCl_3$, $Me_4Si$): δ 166.75, 135.03, 133.14, 131.72, 128.76, 126.85, 117.48, 61.82, 40.23, 37.09, 27.54, 16.59, 14.93, 7.85.
**HR-MS**: *m/z* calcd. $[C_{16}H_{24}N_2O + Na]^+$: 283.1781; found (ESI): 283.1789.

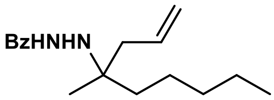


**11d**, *known compound*[7], 66.8 mg, 92% yield.
**$^1$H NMR** (500 MHz, $CDCl_3$, $Me_4Si$): δ 7.79 – 7.70 (m, 2H), 7.50 (t, *J* = 7.4 Hz, 1H), 7.46 (s, 1H), 7.42 (t, *J* = 7.5 Hz, 2H), 6.00 – 5.86 (m, 1H), 5.24 – 4.75 (m, 3H), 2.29 – 2.16 (m, 2H), 1.46 – 1.23 (m, 8H), 1.09 (s, 3H), 0.89 (t, *J* = 7.1 Hz, 3H).

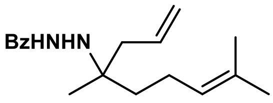


**11e**, *new compound*, yellow oil, 51.4 mg, 90% yield.
**$R_f$** = 0.33 (EtOAc:hexanes = 1:4 v/v).
**$^1$H NMR** (500 MHz, $CDCl_3$, $Me_4Si$): δ 7.73 (dd, *J* = 5.2, 3.3 Hz, 2H), 7.56 – 7.34 (m, 4H), 5.94 (ddt, *J* = 17.6, 10.3, 7.4 Hz, 1H), 5.23 – 4.76 (m, 4H), 2.29 – 2.18 (m, 2H), 2.07 (dd, *J* = 15.8, 7.8 Hz, 2H), 1.68 (s, 3H), 1.62 (s, 3H), 1.53 – 1.41 (m, 2H), 1.11 (s, 3H).
**$^{13}$C NMR** (126 MHz, $CDCl_3$, $Me_4Si$): δ 167.12, 134.67, 133.06, 131.81, 128.79, 126.90, 124.56, 117.96, 59.70, 42.76, 37.86, 25.80, 22.92, 22.51, 17.77.
**HR-MS**: *m/z* calcd. $[C_{18}H_{26}N_2O + Na]^+$: 309.1937; found (ESI): 309.1941.

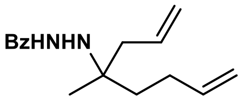


**11f**, *new compound*, yellow oil, 48.3 mg, 93% yield.
**$R_f$** = 0.29 (EtOAc:hexanes = 1:4 v/v).
**$^1$H NMR** (500 MHz, $CDCl_3$, $Me_4Si$): δ 7.79 – 7.69 (m, 2H), 7.63 (m, 2H), 7.42 (t, *J* = 7.5 Hz, 2H), 5.99 – 5.88 (m, 1H), 5.83 (ddt, *J* = 16.8, 10.1, 6.6 Hz, 1H), 5.18 – 5.09 (m, 2H), 5.04 (dd, *J* = 17.1, 1.2 Hz, 1H), 4.95 (d, *J* = 10.1 Hz, 1H), 2.29 – 2.21 (m, 2H), 2.16 (dd, *J* = 15.2, 7.8 Hz, 2H), 1.60 – 1.48 (m, 2H), 1.11 (s, 3H).

**$^{13}$C NMR** (126 MHz, $CDCl_3$, $Me_4Si$): δ 167.20, 138.89, 134.43, 132.92, 131.75, 128.70, 126.84, 117.96, 114.47, 59.54, 42.67, 36.91, 28.07, 22.80.
**HR-MS**: *m/z* calcd. $[C_{16}H_{22}N_2O + H]^+$: 259.1805; found (FAB): 259.1806.

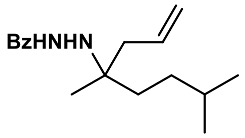

**11g**, *new compound*, yellow oil, 49.4 mg, 90% yield.
**$R_f$** = 0.33 (EtOAc:hexanes = 1:4 v/v).
**$^1$H NMR** (500 MHz, $CDCl_3$, $Me_4Si$): δ 7.73 (d, *J* = 7.4 Hz, 2H), 7.51 (t, *J* = 7.2 Hz, 1H), 7.48 – 7.30 (m, 3H), 5.93 (td, *J* = 17.4, 7.4 Hz, 1H), 5.27 – 4.72 (m, 3H), 2.29 – 2.17 (m, 2H), 1.56 – 1.36 (m, 3H), 1.26 (dd, *J* = 15.2, 7.8 Hz, 2H), 1.09 (s, 3H), 0.90 (d, *J* = 6.6 Hz, 6H).
**$^{13}$C NMR** (126 MHz, $CDCl_3$, $Me_4Si$): δ 167.14, 134.80, 133.12, 131.81, 128.81, 126.89, 117.83, 59.77, 42.67, 35.61, 32.69, 28.74, 22.97, 22.77.
**HR-MS**: *m/z* calcd. $[C_{17}H_{26}N_2O + Na]^+$: 297.1937; found (ESI): 297.1945.

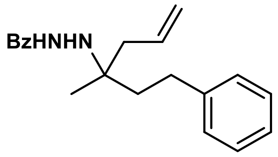

**11h**, *known compound*[8], 56.1 mg, 90% yield.
**$^1$H NMR** (500 MHz, $CDCl_3$, $Me_4Si$): δ 7.70 (d, *J* = 7.6 Hz, 2H), 7.48 (t, *J* = 7.3 Hz, 1H), 7.45 – 7.34 (m, 3H), 7.31 – 7.23 (m, 2H), 7.23 – 7.13 (m, 3H), 5.96 (td, *J* = 17.2, 7.4 Hz, 1H), 5.26 – 5.10 (m, 2H), 2.72 (t, *J* = 8.5 Hz, 2H), 2.38 – 2.23 (m, 2H), 1.84 – 1.65 (m, 2H), 1.16 (s, 3H).

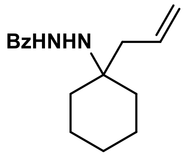

**11i**, *known compound*[6], 43.1 mg, 83% yield.
**$^1$H NMR** (500 MHz, $CDCl_3$, $Me_4Si$): δ 7.73 (d, *J* = 7.3 Hz, 2H), 7.55 – 7.35 (m, 4H), 6.01 (ddd, *J* = 24.1, 10.5, 7.4 Hz, 1H), 5.19 – 5.08 (m, 2H), 2.27 (d, *J* = 7.4 Hz, 2H), 1.73 – 1.60 (m, 2H), 1.58 – 1.49 (m, 4H), 1.48 – 1.34 (m, 4H).

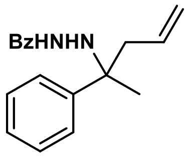

**15a**, *known compound*[8], 49.0 mg, 88% yield.
**$^1$H NMR** (500 MHz, $CDCl_3$, $Me_4Si$): δ 7.63 – 7.57 (m, 2H), 7.55 (d, *J* = 7.4 Hz, 2H), 7.44 (t, *J* = 7.4 Hz, 1H), 7.41 – 7.32 (m, 4H), 7.31 – 7.25 (m, 1H), 7.23 (s, 1H), 5.74 – 5.59 (m, 1H), 5.16 – 4.99 (m, 2H), 2.62 (dd, *J* = 13.7, 6.6 Hz, 1H), 2.51 (dd, *J* = 13.7, 8.0 Hz, 1H), 1.55 (s, 3H).

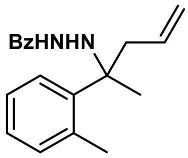

**15b**, *new compound*, yellow waxy solid, 43.6 mg, 74% yield.
**$R_f$** = 0.43 (EtOAc:hexanes = 1:4 v/v).
**$^1$H NMR** (500 MHz, $CDCl_3$, $Me_4Si$): δ 7.55 (d, *J* = 7.3 Hz, 2H), 7.44 (t, *J* = 7.4 Hz, 1H), 7.40 – 7.29 (m, 3H), 7.23 – 7.14 (m, 3H), 7.07 (d, *J* = 4.2 Hz, 1H), 5.71 – 5.54 (m, 1H), 5.38 (d, *J* = 6.7 Hz, 1H), 5.18 – 4.97 (m, 2H), 2.85 (dd, *J* = 13.8, 6.3 Hz, 1H), 2.73 (s, 3H), 2.56 (dd, *J* = 13.8, 8.2 Hz, 1H), 1.62 (s, 3H).
**$^{13}$C NMR** (126 MHz, $CDCl_3$, $Me_4Si$): δ 166.33, 140.76, 137.11, 133.62, 133.21, 132.91, 131.57, 128.58, 128.17, 127.37, 126.73, 125.84, 118.42, 64.03, 43.28, 24.37, 22.69.
**HR-MS**: *m/z* calcd. $[C_{19}H_{22}N_2O + Na]^+$: 317.1624; found (ESI): 317.1630.

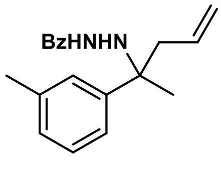

**15c**, *new compound*, yellow waxy solid, 50.1 mg, 85% yield.
**$R_f$** = 0.33 (EtOAc:hexanes = 1:4 v/v).
**$^1$H NMR** (500 MHz, $CDCl_3$, $Me_4Si$): δ 7.59 (d, *J* = 7.3 Hz, 2H), 7.44 (t, *J* = 7.4 Hz, 1H), 7.40 – 7.31 (m, 4H), 7.29 – 7.22 (m, 2H), 7.09 (d, *J* = 7.4 Hz, 1H), 5.74

– 5.61 (m, 1H), 5.42 (s, 1H), 5.19 – 4.97 (m, 2H), 2.61 (dd, *J* = 13.7, 6.5 Hz, 1H), 2.50 (dd, *J* = 13.7, 8.0 Hz, 1H), 2.37 (s, 3H), 1.53 (s, 3H).
**$^{13}$C NMR** (126 MHz, $CDCl_3$, $Me_4Si$): δ 166.57, 144.37, 138.10, 133.77, 133.00, 131.67, 128.66, 128.43, 127.90, 127.23, 126.84, 123.58, 118.55, 62.45, 46.17, 22.82, 21.78.
**HR-MS**: *m/z* calcd. $[C_{19}H_{22}N_2O_3 + H]^+$: 295.1805; found (FAB): 295.1806.

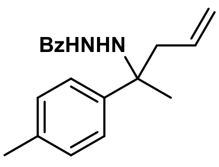


**15d**, *known compound*[7], 53.8 mg, 91% yield.
**$^{1}$H NMR** (500 MHz, $CDCl_3$, $Me_4Si$): δ 7.58 (d, *J* = 7.5 Hz, 2H), 7.48 – 7.37 (m, 4H), 7.32 (t, *J* = 7.6 Hz, 2H), 7.16 (d, *J* = 8.0 Hz, 2H), 5.65 (dq, *J* = 9.9, 7.7 Hz, 1H), 5.41 (s, 1H), 5.14 – 4.98 (m, 2H), 2.60 (dd, *J* = 13.7, 6.5 Hz, 1H), 2.48 (dd, *J* = 13.7, 8.0 Hz, 1H), 2.33 (s, 3H), 1.51 (s, 3H).

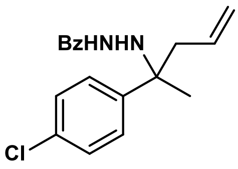


**15e**, *new compound*, yellow oil, 57.5 mg, 91% yield.
**$R_f$** = 0.36 (EtOAc:hexanes = 1:4 v/v).
**$^{1}$H NMR** (500 MHz, $CDCl_3$, $Me_4Si$): δ 7.59 (d, *J* = 7.3 Hz, 2H), 7.53 – 7.42 (m, 3H), 7.41 – 7.27 (m, 5H), 5.63 (td, *J* = 16.3, 7.6 Hz, 1H), 5.41 (s, 1H), 5.18 – 4.96 (m, 2H), 2.57 (dd, *J* = 13.2, 6.3 Hz, 1H), 2.47 (dd, *J* = 13.1, 8.2 Hz, 1H), 1.52 (s, 3H).
**$^{13}$C NMR** (126 MHz, $CDCl_3$, $Me_4Si$): δ 166.84, 142.98, 133.14, 132.87, 132.64, 131.75, 128.62, 128.52, 128.08, 126.77, 118.89, 62.25, 46.07, 22.59.
**HR-MS**: *m/z* calcd. $[C_{18}H_{19}ClN_2O + H]^+$: 315.1259; found (FAB): 315.1261.

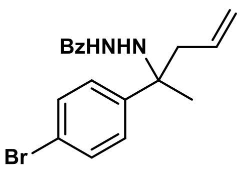


**15f**, *known compound*[7], 61.2 mg, 85% yield.
**$^{1}$H NMR** (500 MHz, $CDCl_3$, $Me_4Si$): δ 7.59 (d, *J* = 7.5 Hz, 2H), 7.56 (s, 1H), 7.42 (dt, *J* = 12.5, 8.6 Hz, 5H), 7.33 (t, *J* = 7.7 Hz, 2H), 5.60 (dt, *J* = 17.3, 7.7 Hz, 1H), 5.40 (s, 1H), 5.09 – 4.97 (m, 2H), 2.55 (dd, *J* = 13.7, 6.6 Hz, 1H), 2.45 (dd, *J* = 13.7, 8.0 Hz, 1H), 1.49 (s, 3H).

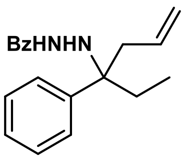


**15g**, *known compound*[7], 44.8 mg, 76% yield.
**$^{1}$H NMR** (500 MHz, $CDCl_3$, $Me_4Si$): δ 7.56 (d, *J* = 7.4 Hz, 2H), 7.53 (d, *J* = 7.6 Hz, 2H), 7.45 (t, *J* = 7.4 Hz, 1H), 7.42 – 7.32 (m, 4H), 7.27 (t, *J* = 7.3 Hz, 1H), 7.07 (s, 1H), 5.85 (ddt, *J* = 17.1, 10.1, 7.1 Hz, 1H), 5.58 (s, 1H), 5.18 – 5.05 (m, 2H), 2.72 – 2.61 (m, 2H), 1.96 – 1.75 (m, 2H), 0.82 (t, *J* = 7.4 Hz, 3H).

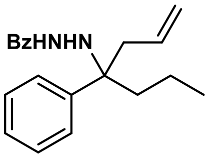


**15h**, *known compound*[8], 52.5 mg, 85% yield.
**$^{1}$H NMR** (500 MHz, $CDCl_3$, $Me_4Si$): δ 7.56 (d, *J* = 7.6 Hz, 2H), 7.53 (d, *J* = 7.7 Hz, 2H), 7.44 (t, *J* = 7.3 Hz, 1H), 7.42 – 7.31 (m, 4H), 7.31 – 7.22 (m, 1H), 7.10 (s, 1H), 5.87 (td, *J* = 17.0, 7.2 Hz, 1H), 5.55 (s, 1H), 5.22 – 5.04 (m, 2H), 2.76 – 2.58 (m, 2H), 1.77 (m, 2H), 1.37 – 1.16 (m, 2H), 0.84 (t, *J* = 7.2 Hz, 3H).

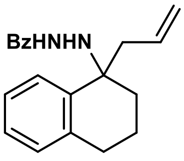


**15i**, *new compound*, transparent oil, 40.8 mg, 67% yield.
**$R_f$** = 0.43 (EtOAc:hexanes = 1:4 v/v).
**$^{1}$H NMR** (500 MHz, $CDCl_3$, $Me_4Si$): δ 7.67 (d, *J* = 7.5 Hz, 1H), 7.62 (d, *J* = 7.4 Hz, 2H), 7.46 (t, *J* = 7.0 Hz, 1H), 7.37 (t, *J* = 7.3 Hz, 2H), 7.30 – 7.14 (m, 3H), 7.10 (d, *J* = 7.1 Hz, 1H), 5.75 (td, *J* = 16.5, 8.5 Hz, 1H), 5.41 (s, 1H), 5.23 – 5.02 (m, 2H), 2.85 – 2.65 (m, 3H), 2.52 (dd, *J* = 13.6, 8.5 Hz, 1H), 2.01 – 1.73 (m, 4H).

**$^{13}$C NMR** (126 MHz, $CDCl_3$, $Me_4Si$): δ 166.66, 138.87, 138.61, 133.78, 132.83, 131.64, 129.34, 128.62, 127.13, 127.00, 126.76, 126.14, 118.75, 61.24, 45.16, 30.69, 30.01, 19.73.
**HR-MS**: *m/z* calcd. $[C_{20}H_{22}N_2O + Na]^+$: 329.1624; found (ESI): 329.1629.

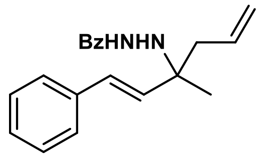


**17a**, *known compound*[7], 49.0 mg, 80% yield.
**$^1$H NMR** (500 MHz, $CDCl_3$, $Me_4Si$): δ 7.75 (s, 1H), 7.68 (d, *J* = 7.5 Hz, 2H), 7.43 (t, *J* = 7.4 Hz, 1H), 7.40 – 7.32 (m, 4H), 7.28 (t, *J* = 7.6 Hz, 2H), 7.20 (t, *J* = 7.3 Hz, 1H), 6.49 (d, *J* = 16.3 Hz, 1H), 6.25 (d, *J* = 16.3 Hz, 1H), 5.88 (ddt, *J* = 14.8, 10.2, 7.3 Hz, 1H), 5.25 (s, 1H), 5.19 – 5.01 (m, 2H), 2.51 – 2.33 (m, 2H), 1.32 (s, 3H).

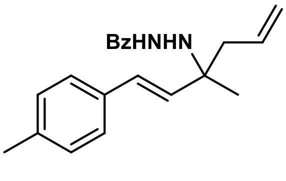


**17b**, *new compound*, yellow waxy oil, 35.2 mg, 55% yield.
**$R_f$** = 0.23 (EtOAc:hexanes = 1:4 v/v).
**$^1$H NMR** (500 MHz, $CDCl_3$, $Me_4Si$): δ 7.68 (d, *J* = 7.4 Hz, 2H), 7.51 (s, 1H), 7.46 (t, *J* = 7.4 Hz, 1H), 7.37 (t, *J* = 7.6 Hz, 2H), 7.27 (d, *J* = 8.0 Hz, 2H), 7.11 (d, *J* = 7.9 Hz, 2H), 6.48 (d, *J* = 16.3 Hz, 1H), 6.20 (d, *J* = 16.3 Hz, 1H), 5.95 – 5.83 (m, 1H), 5.22 (s, 1H), 5.18 – 5.06 (m, 2H), 2.49 – 2.36 (m, 2H), 2.32 (s, 3H), 1.32 (s, 3H).
**$^{13}$C NMR** (126 MHz, $CDCl_3$, $Me_4Si$): δ 166.93, 137.40, 134.10, 133.69, 132.98, 132.96, 131.67, 129.73, 129.28, 128.66, 126.79, 126.34, 118.47, 61.16, 44.08, 22.16, 21.16.
**HR-MS**: *m/z* calcd. $[C_{21}H_{24}N_2O + Na]^+$: 343.1781; found (ESI): 343.1785.

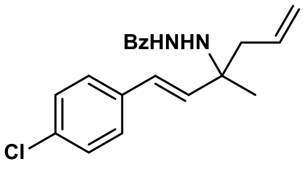


**17c**, *new compound*, yellow waxy oil, 45.0 mg, 66% yield.
**$R_f$** = 0.19 (EtOAc:hexanes = 1:4 v/v).
**$^1$H NMR** (500 MHz, $CDCl_3$, $Me_4Si$): δ 7.77 (s, 1H), 7.68 (d, *J* = 7.5 Hz, 2H), 7.50 – 7.43 (m, 1H), 7.36 (t, *J* = 7.6 Hz, 2H), 7.25 (d, *J* = 8.5 Hz, 2H), 7.21 (d, *J* = 8.5 Hz, 2H), 6.43 (d, *J* = 16.3 Hz, 1H), 6.22 (d, *J* = 16.3 Hz, 1H), 5.86 (dt, *J* = 16.6, 7.4 Hz, 1H), 5.21 – 5.07 (m, 2H), 2.50 – 2.33 (m, 2H), 1.31 (s, 3H).
**$^{13}$C NMR** (126 MHz, $CDCl_3$, $Me_4Si$): δ 167.27, 135.45, 134.80, 133.48, 133.07, 132.82, 131.76, 128.66, 128.47, 127.62, 126.80, 118.59, 61.24, 43.89, 22.06.
**HR-MS**: *m/z* calcd. $[C_{20}H_{21}ClN_2O + Na]^+$: 363.1235; found (ESI): 363.1241.

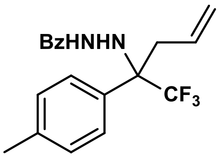


**19a**, *known compound*[9], 58.9 mg, 84% yield.
**$^1$H NMR** (500 MHz, $CDCl_3$, $Me_4Si$): δ 7.65 (d, *J* = 7.6 Hz, 2H), 7.54 (d, *J* = 7.9 Hz, 2H), 7.50 (t, *J* = 7.4 Hz, 1H), 7.41 (t, *J* = 7.6 Hz, 2H), 7.33 (d, *J* = 7.7 Hz, 1H), 7.22 (d, *J* = 8.0 Hz, 2H), 5.96 (d, *J* = 7.9 Hz, 1H), 5.72 (td, *J* = 16.8, 7.1 Hz, 1H), 5.17 (d, *J* = 17.0 Hz, 1H), 5.09 (d, *J* = 10.2 Hz, 1H), 3.01 (dd, *J* = 15.1, 6.8 Hz, 1H), 2.93 (dd, *J* = 15.1, 7.0 Hz, 1H), 2.36 (s, 3H).

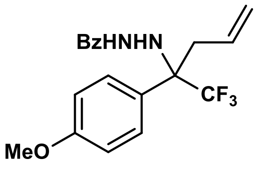


**19b**, *new compound*, white solid, 58.4 mg, 80% yield.
**$R_f$** = 0.40 (EtOAc:hexanes = 1:4 v/v).
**mp**: 108 °C.
**$^1$H NMR** (500 MHz, $CDCl_3$, $Me_4Si$): δ 7.66 (d, *J* = 7.2 Hz, 2H), 7.59 (d, *J* = 8.7 Hz, 2H), 7.51 (t, *J* = 7.4 Hz, 1H), 7.42 (t, *J* = 7.7 Hz, 2H), 7.34 (d, *J* = 7.8 Hz, 1H), 6.93 (d, *J* = 9.0 Hz, 2H), 5.92 (d, *J* = 7.9 Hz, 1H), 5.74 (dq, *J* = 9.9, 7.0 Hz, 1H), 5.17 (dd, *J* = 17.0, 1.5 Hz, 1H), 5.10 (dd, *J* = 10.2, 1.3 Hz, 1H), 3.82 (s, 3H), 3.01 (dd, *J* = 15.2, 6.8 Hz, 1H), 2.93 (dd, *J* = 15.1, 7.1 Hz, 1H).

**$^{13}$C NMR** (126 MHz, $CDCl_3$, $Me_4Si$): δ 166.45, 159.65, 132.37, 132.04, 131.42, 129.05, 128.77, 126.87, 126.54, 126.47 (q, $^{1}J_{C\text{-}F}$ = 287.3 Hz), 119.51, 113.93, 67.42 (q, $^{2}J_{C\text{-}F}$ = 24.1 Hz), 55.25, 36.59.
**HR-MS**: *m/z* calcd. $[C_{19}H_{19}F_3N_2O_2 + Na]^+$: 387.1291; found (ESI): 387.1295.

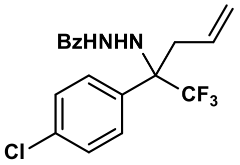


**19c**, *known compound*[9], 65.7 mg, 89% yield.
**$^{1}$H NMR** (500 MHz, $CDCl_3$, $Me_4Si$): δ 7.69 – 7.56 (m, 4H), 7.53 – 7.47 (m, 1H), 7.47 – 7.28 (m, 5H), 5.89 (d, *J* = 6.3 Hz, 1H), 5.78 – 5.61 (m, 1H), 5.29 – 4.98 (m, 2H), 3.00 (dd, *J* = 14.2, 5.7 Hz, 1H), 2.90 (dd, *J* = 14.3, 6.7 Hz, 1H).

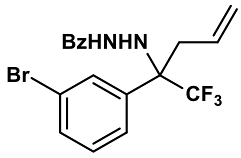


**19d**, *new compound*, transparent oil, 57.8 mg, 70% yield.
**$R_f$** = 0.50 (EtOAc:hexanes = 1:19 v/v).
**$^{1}$H NMR** (500 MHz, $CDCl_3$, $Me_4Si$): δ 7.84 (s, 1H), 7.66 (d, *J* = 7.4 Hz, 2H), 7.61 (d, *J* = 8.0 Hz, 1H), 7.56 – 7.48 (m, 2H), 7.42 (t, *J* = 7.7 Hz, 2H), 7.35 – 7.27 (m, 2H), 5.93 (d, *J* = 7.6 Hz, 1H), 5.71 (td, *J* = 17.0, 7.1 Hz, 1H), 5.19 (dd, *J* = 17.0, 1.2 Hz, 1H), 5.14 (d, *J* = 10.2 Hz, 1H), 3.01 (dd, *J* = 15.1, 6.8 Hz, 1H), 2.89 (dd, *J* = 15.1, 7.2 Hz, 1H).
**$^{13}$C NMR** (126 MHz, $CDCl_3$, $Me_4Si$): δ 166.79, 137.25, 132.19, 132.17, 131.92, 130.89, 130.74, 130.12, 128.81, 126.93, 126.43, 126.14 (q, $^{1}J_{C\text{-}F}$ = 287.6 Hz), 122.84, 120.19, 67.64 (q, $^{2}J_{C\text{-}F}$ = 24.4 Hz), 36.97.
**HR-MS**: *m/z* calcd. $[C_{18}H_{16}BrF_3N_2O + Na]^+$: 435.0290; found (ESI): 435.0297.

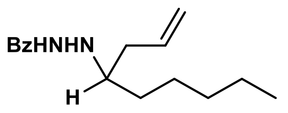


**21a**, *known compound*[10], 26.0 mg, 50% yield.
**$^{1}$H NMR** (500 MHz, $CDCl_3$, $Me_4Si$): δ 8.10 (d, *J* = 7.6 Hz, 1H), 7.78 (d, *J* = 7.5 Hz, 2H), 7.52 (t, *J* = 7.3 Hz, 1H), 7.45 (t, *J* = 7.5 Hz, 2H), 5.90 (ddd, *J* = 24.3, 10.0, 7.3 Hz, 1H), 5.15 (m, 2H), 3.11 – 3.00 (m, 1H), 2.38 – 2.28 (m, 1H), 2.27 – 2.18 (m, 1H), 1.57 – 1.28 (m, 8H), 0.88 (t, *J* = 6.7 Hz, 3H).

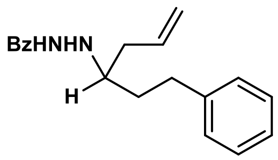


**21b**, *known compound*[11], 28.9 mg, 49% yield.
**$^{1}$H NMR** (500 MHz, $CDCl_3$, $Me_4Si$): δ 7.86 (s, 1H), 7.72 (d, *J* = 7.5 Hz, 2H), 7.49 (t, *J* = 7.4 Hz, 1H), 7.40 (t, *J* = 7.6 Hz, 2H), 7.30 – 7.23 (m, 2H), 7.22 – 7.13 (m, 3H), 5.89 (ddt, *J* = 17.1, 10.0, 7.2 Hz, 1H), 5.19 – 5.10 (m, 2H), 3.16 – 3.05 (m, 1H), 2.81 – 2.64 (m, 2H), 2.39 – 2.22 (m, 2H), 1.88 – 1.71 (m, 2H).

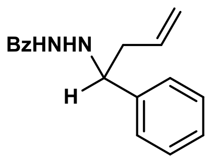


**21c**, *known compound*[10], 33.0 mg, 62% yield.
**$^{1}$H NMR** (500 MHz, $CDCl_3$, $Me_4Si$): δ 7.65 (s, 1H), 7.59 (d, *J* = 7.4 Hz, 2H), 7.45 (t, *J* = 7.4 Hz, 1H), 7.40 – 7.30 (m, 6H), 7.30 – 7.24 (m, 1H), 5.82 (ddd, *J* = 16.8, 8.9, 7.2 Hz, 1H), 5.23 – 5.05 (m, 2H), 4.17 (t, *J* = 7.0 Hz, 1H), 2.61 – 2.41 (m, 2H).

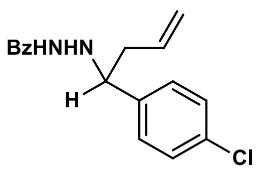


**21d**, *known compound*[12], 40.3 mg, 66% yield.
**$^{1}$H NMR** (500 MHz, $CDCl_3$, $Me_4Si$): δ 7.59 (d, *J* = 7.3 Hz, 2H), 7.48 (dd, *J* = 10.5, 4.2 Hz, 2H), 7.38 (t, *J* = 7.7 Hz, 2H), 7.31 (s, 4H), 5.86 – 5.70 (m, 1H), 5.32 – 5.03 (m, 3H), 4.16 (t, *J* = 7.0 Hz, 1H), 2.47 (qt, *J* = 14.0, 7.0 Hz, 2H).

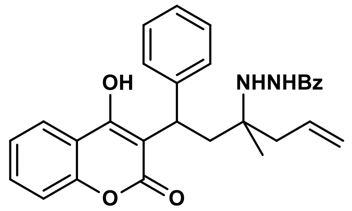

**23a**, *new compound*, transparent oil, 37.4 mg, 40% yield.
$\mathbf{R_f}$ = 0.44 (EtOAc:hexanes = 1:3 v/v).
**$^1$H NMR** (500 MHz, $CDCl_3$, $Me_4Si$): δ 11.92 (s, 1H), 8.00 (s, 1H), 7.81 – 7.65 (m, 3H), 7.50 (t, *J* = 7.4 Hz, 1H), 7.43 – 7.31 (m, 3H), 7.25 – 7.10 (m, 5H), 6.90 – 6.76 (m, 2H), 5.90 – 5.73 (m, 1H), 5.23 – 5.03 (m, 2H), 4.73 (s, 1H), 3.95 (t, *J* = 12.3 Hz, 1H), 2.66 (dd, *J* = 13.9, 7.2 Hz, 1H), 2.53 – 2.35 (m, 2H), 2.07 (dd, *J* = 13.9, 2.7 Hz, 1H), 1.54 (s, 3H).
**$^{13}$C NMR** (126 MHz, $CDCl_3$, $Me_4Si$): δ 202.14, 168.13, 167.25, 162.71, 140.75, 136.70, 132.68, 132.36, 131.85, 131.41, 128.97, 128.61, 127.55, 127.47, 126.95, 119.69, 119.11, 118.19, 63.37, 45.06, 41.25, 38.09, 29.70, 24.37.
**HR-MS**: *m/z* calcd. $[C_{29}H_{28}N_2O_4 + Na]^+$: 491.1941; found (ESI): 491.1947.

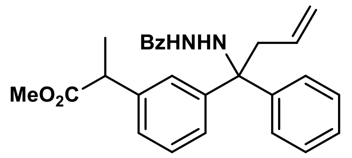

**23b**, *new compound*, transparent oil, 38.6 mg, 45% yield.
$\mathbf{R_f}$ = 0.42 (EtOAc:hexanes = 1:2 v/v).
**$^1$H NMR** (500 MHz, $CDCl_3$, $Me_4Si$): δ 7.63 – 7.27 (m, 12H), 7.26 – 7.15 (m, 2H), 7.06 (d, *J* = 3.9 Hz, 1H), 5.77 (s, 1H), 5.73 – 5.55 (m, 1H), 5.17 – 4.93 (m, 2H), 3.69 (q, *J* = 7.0 Hz, 1H), 3.59 (d, *J* = 1.5 Hz, 3H), 3.11 (d, *J* = 6.8 Hz, 2H), 1.45 (d, *J* = 7.2 Hz, 3H).
**$^{13}$C NMR** (126 MHz, $CDCl_3$, $Me_4Si$): δ 174.94, 174.93, 165.74, 144.05, 143.50, 140.38, 140.35, 133.40, 132.90, 131.63, 128.61, 128.52, 128.19, 127.73, 127.20, 127.16, 126.70, 126.65, 126.12, 126.03, 118.89, 68.50, 51.96, 45.45, 42.08, 42.05, 18.57, 18.50.
**HR-MS**: *m/z* calcd. $[C_{27}H_{28}N_2O_3 + Na]^+$: 451.1992; found (ESI): 451.2001.

§ Note: Due to the rotameric conformation, $^{13}$C NMR spectrum shows excess number of peaks.

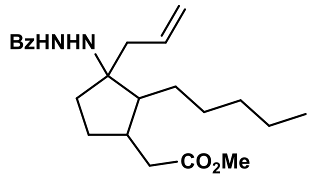

**23c**, *new compound*, transparent oil, 56.4 mg, 73% yield.
$\mathbf{R_f}$ = 0.66 (EtOAc:hexanes = 1:2 v/v).
**$^1$H NMR** (500 MHz, $CDCl_3$, $Me_4Si$): δ 7.86 – 7.67 (m, 2H), 7.63 – 7.32 (m, 4H), 6.13 – 5.80 (m, 1H), 5.23 – 5.01 (m, 3H), 3.65 (s, 3H), 2.65 – 2.50 (m, 1H), 2.49 – 2.25 (m, 3H), 2.24 – 2.02 (m, 2H), 1.92 – 1.50 (m, 4H), 1.45 – 1.26 (m, 8H), 0.96 – 0.82 (m, 3H).
**$^{13}$C NMR** (126 MHz, $CDCl_3$, $Me_4Si$): δ 173.76, 173.49, 167.23, 167.05, 135.13, 135.07, 132.92, 132.85, 131.74, 128.70, 126.82, 126.80, 117.90, 70.29, 70.12, 51.68, 51.45, 50.73, 41.59, 41.43, 40.99, 40.71, 40.58, 38.02, 32.56, 32.53, 30.11, 29.08, 28.96, 28.82, 28.57, 28.51, 22.59, 22.56, 14.12, 14.09.
**HR-MS**: *m/z* calcd. $[C_{23}H_{34}N_2O_3 + Na]^+$: 409.2462; found (ESI): 409.2468.

§ Note: Based on the $^1$H NMR spectrum integration ratio comparison at δ 6.13 – 5.80, corresponding to one proton, diastereomeric ratio was calculated in 2.67:1. Furthermore, with described issue, $^{13}$C NMR spectrum shows excess number of peaks.

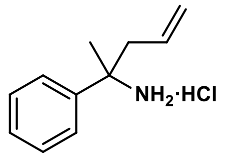

**25**, *known compound*[14], 19.1 mg, 97% yield.
**$^1$H NMR** (500 MHz, $CDCl_3$, $Me_4Si$): δ 9.02 (s, 3H), 7.54 (d, *J* = 7.6 Hz, 2H), 7.36 (t, *J* = 7.5 Hz, 2H), 7.30 (t, *J* = 7.3 Hz, 1H), 5.50 (ddt, *J* = 17.3, 10.0, 7.2 Hz, 1H), 5.17 (d, *J* = 17.0 Hz, 1H), 5.14 – 5.02 (m, 1H), 2.89 (dd, *J* = 14.1, 7.5 Hz, 1H), 2.82 (dd, *J* = 14.1, 7.1 Hz, 1H), 1.83 (s, 3H).

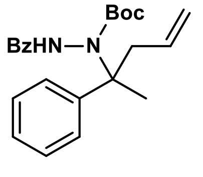


**26**, *new compound*, white solid, 803.8 mg, 70% yield (mixture of rotamers).
**$R_f$** = 0.22 (EtOAc:hexanes = 1:4 v/v).
**$^1$H NMR** (500 MHz, $CDCl_3$, $Me_4Si$, major and minor isomers): δ 8.32 – 7.88 (m, 1H), 7.86 – 7.76 (m, 2H), 7.75 – 7.65 (m, 2H), 7.57 – 7.47 (m, 1H), 7.46 – 7.37 (m, 2H), 7.33 (t, *J* = 7.6 Hz, 2H), 7.22 (t, *J* = 7.2 Hz, 1H), 5.85 (td, *J* = 16.3, 9.1 Hz, 0.78H), 5.45 (td, *J* = 16.8, 8.0 Hz, 0.23H), 5.31 – 5.06 (m, 1.64H), 5.00 – 4.82 (m, 0.48H), 3.29 – 2.68 (m, 2H), 1.69 (d, *J* = 10.9 Hz, 3H), 1.16 (s, 9H).
**$^{13}$C NMR** (126 MHz, $CDCl_3$, $Me_4Si$, major and minor isomers): δ 168.45, 168.19, 148.42, 146.51, 134.20, 134.11, 132.65, 132.07, 131.99, 128.70, 128.60, 128.06, 127.96, 127.40, 127.31, 126.28, 126.16, 125.91, 125.11, 119.76, 118.11, 81.73, 81.60, 66.57, 66.52, 45.91, 27.93, 27.89, 26.78, 22.96.

§ Note: Inseparable mixture of rotamers (major and minor). Based on the $^1$H NMR integration ratio at (δ 5.85, 5.45, 5.31 – 5.06, 5.00 – 4.82), given in non-integer (decimal) values, rotameric ratio was determined as 3.4:1. Due to the unstable properties (*e.g.*, hygroscopicity, decomposition), this compound was immediately used to the next step without further analysis (*e.g.*, mp, HR-MS).

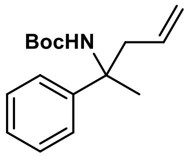


**27**, *known compound*[13], 364.3 mg, 92% yield.
**$^1$H NMR** (500 MHz, $CDCl_3$, $Me_4Si$): δ 7.44 – 7.28 (m, 4H), 7.21 (t, J = 7.0 Hz, 1H), 5.83 – 5.52 (m, 1H), 5.25 – 5.05 (m, 2H), 4.93 (s, 1H), 2.74 (s, 1H), 2.53 (s, 1H), 1.67 (s, 3H), 1.40 (s, 9H).

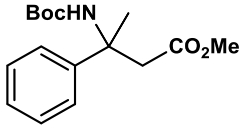


**29**, *new compound*, transparent oil, 18.7 mg, 64% yield.
**$R_f$** = 0.32 (EtOAc:hexanes = 1:4 v/v).
**$^1$H NMR** (500 MHz, $CDCl_3$, $Me_4Si$): δ 7.44 – 7.28 (m, 4H), 7.25 – 7.18 (m, 1H), 5.74 (s, 1H), 3.62 (s, 3H), 3.03 (d, *J* = 14.2 Hz, 1H), 2.80 (d, *J* = 14.1 Hz, 1H), 1.76 (s, 3H), 1.38 (s, 9H).
**$^{13}$C NMR** (126 MHz, $CDCl_3$, $Me_4Si$): δ 171.45, 154.48, 128.38, 126.83, 124.69, 79.30, 56.30, 51.66, 45.71, 28.29, 27.15.
**HR-MS**: *m/z* calcd. $[C_{16}H_{23}NO_4 + Na]^+$: 316.1519; found (ESI): 316.1526.

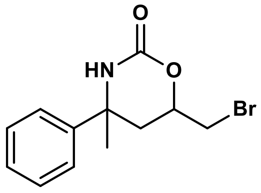


**30**, *new compound*, white solid, 19.9 mg, 70% yield.
**$R_f$** = 0.12 (EtOAc:hexanes = 1:2 v/v).
**mp**: 196 °C.
**$^1$H NMR** (500 MHz, $CDCl_3$, $Me_4Si$): δ 7.49 – 7.28 (m, 5H), 6.99 (s, 1H), 4.12 – 3.92 (m, 1H), 3.49 – 3.28 (m, 2H), 2.51 (d, *J* = 13.6 Hz, 1H), 2.02 (t, *J* = 12.7 Hz, 1H), 1.66 (s, 3H).
**$^{13}$C NMR** (126 MHz, $CDCl_3$, $Me_4Si$): δ 153.74, 144.93, 128.96, 127.59, 124.96, 73.19, 56.86, 39.84, 32.97, 31.17.
**HR-MS**: *m/z* calcd. $[C_{12}H_{14}BrNO_2 + Na]^+$: 306.0100; found (ESI): 306.0108.

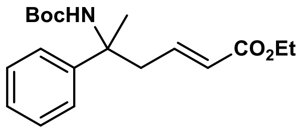


**31**, *new compound*, transparent oil, 32.4 mg, 97% yield.
**$R_f$** = 0.37 (EtOAc:hexanes = 1:4 v/v).
**$^1$H NMR** (500 MHz, $CDCl_3$, $Me_4Si$): δ 7.46 – 7.28 (m, 4H), 7.26 – 7.18 (m, 1H), 6.84 (dt, *J* = 15.4, 7.6 Hz, 1H), 5.90 (d, *J* = 15.6 Hz, 1H), 4.91 (s, 1H), 4.19 (d, *J* = 7.1 Hz, 1H), 4.16 (d, *J* = 7.1 Hz, 1H), 3.12 (s, 1H), 2.81 (s, 1H), 1.61 (s, 3H), 1.48 – 1.17 (m, 12H).

$^{13}$**C NMR** (126 MHz, $CDCl_3$, $Me_4Si$): δ 166.18, 154.11, 145.77, 143.91, 128.44, 126.85, 124.97, 124.88, 79.37, 60.29, 57.40, 42.74, 28.30, 27.73, 14.24.
**HR-MS**: *m/z* calcd. $[C_{19}H_{27}NO_4 + Na]^+$: 356.1832; found (ESI): 356.1838.

# 5. NMR Spectra of the Products

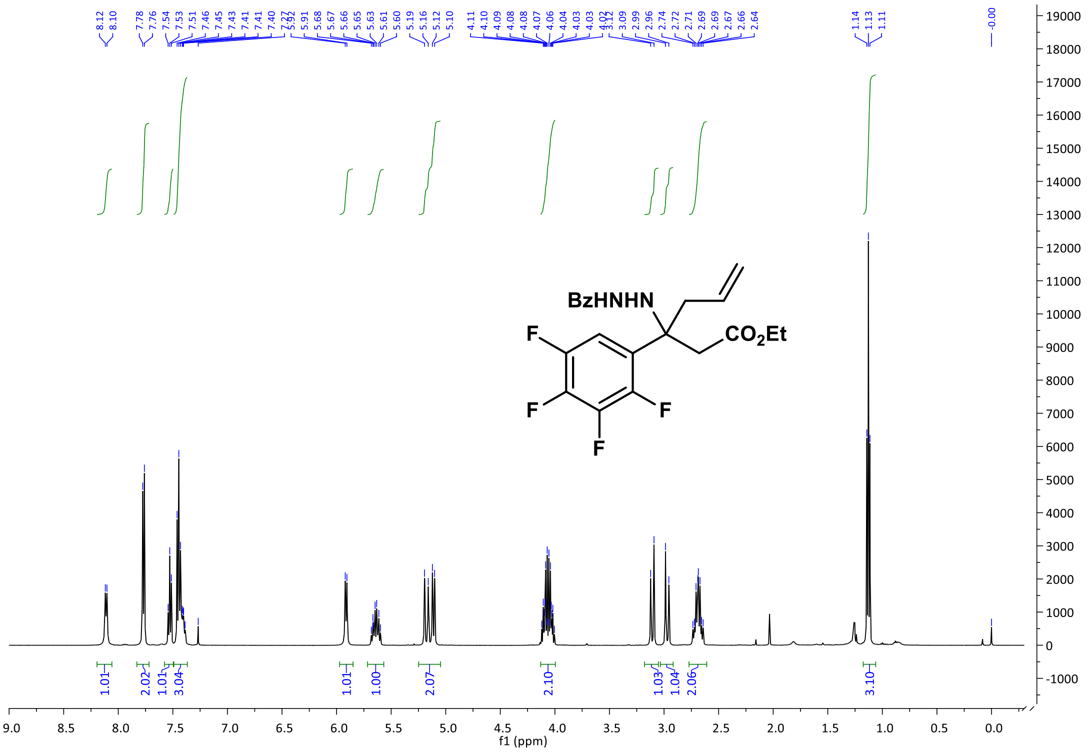

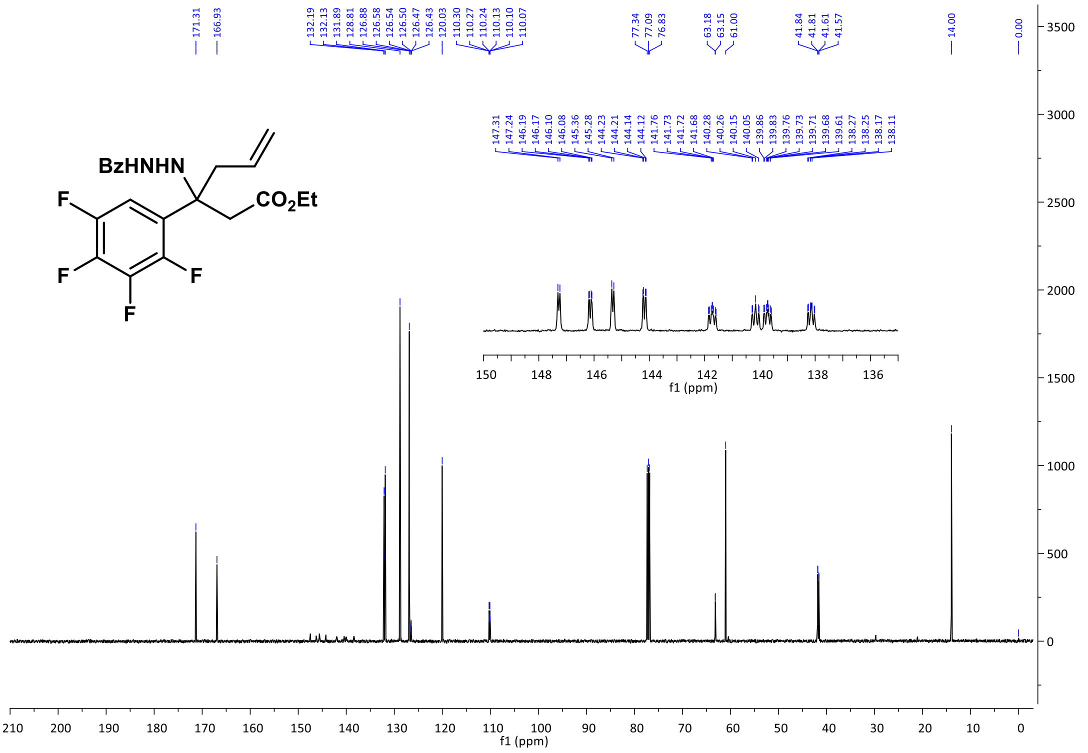

**Supplementary Figure 3.** $^{1}$H and $^{13}$C NMR spectra (**4a**)

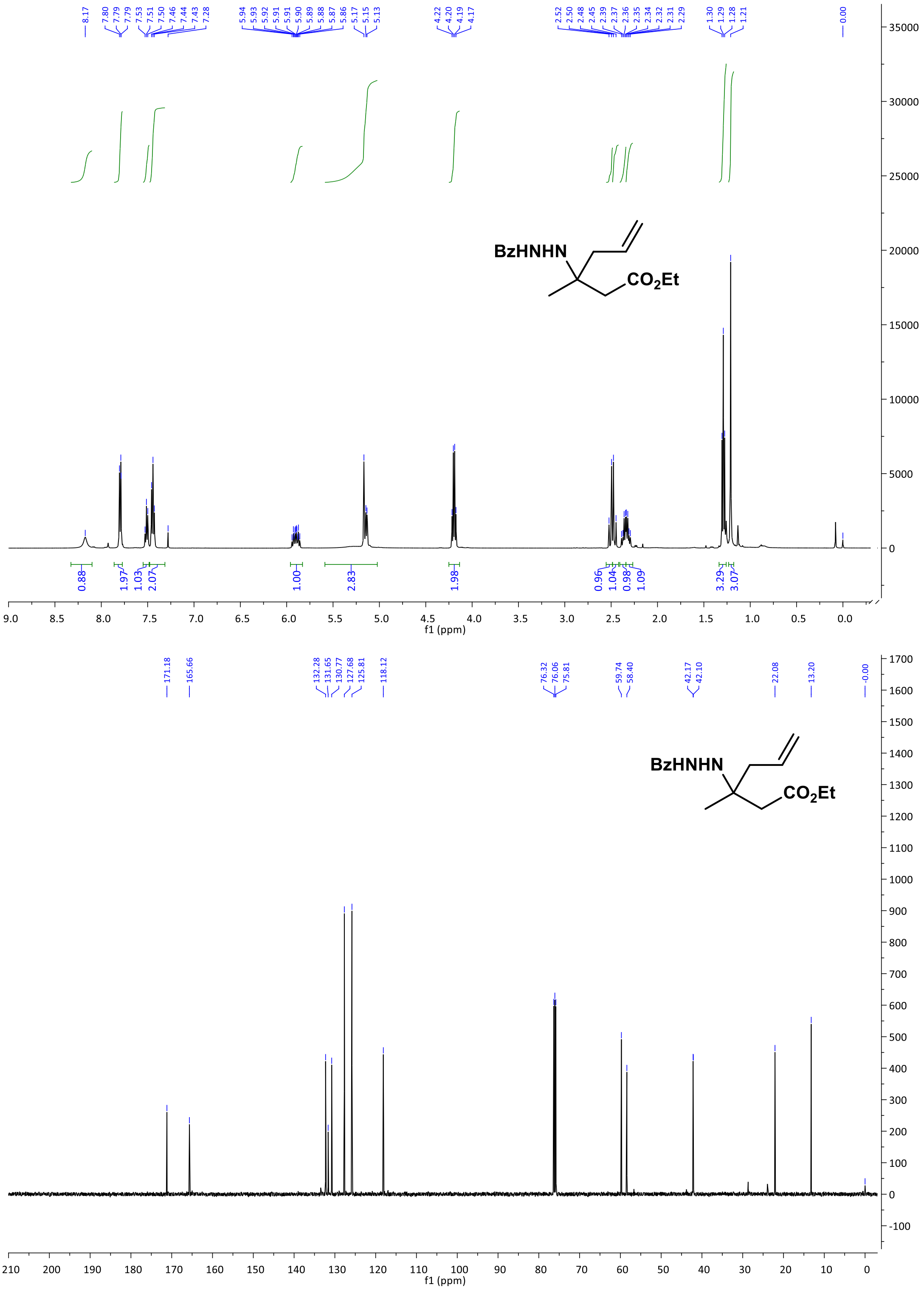


**Supplementary Figure 4.** $^{1}H$ and $^{13}C$ NMR spectra (**4b**)

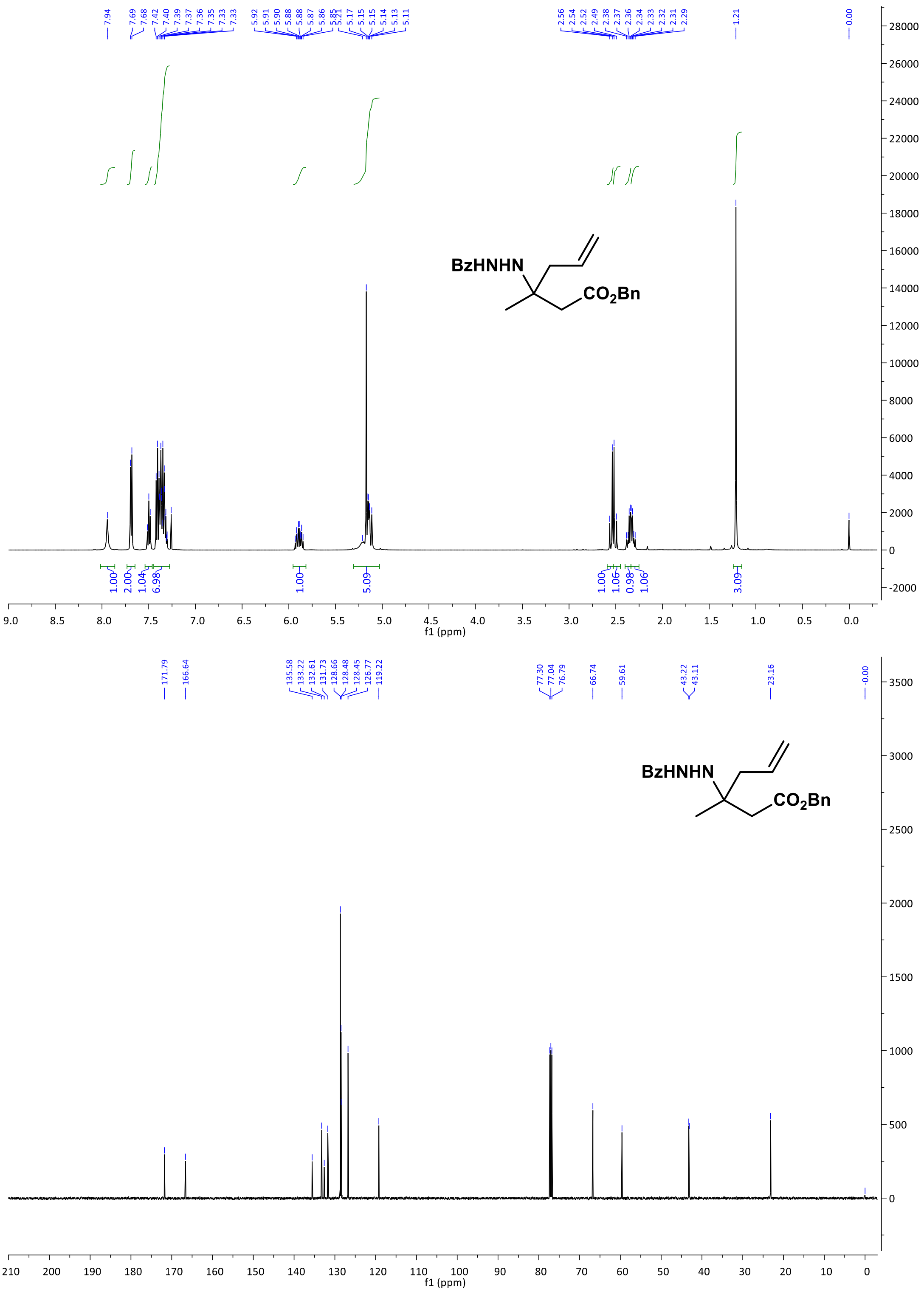


**Supplementary Figure 5.** $^1$H and $^{13}$C NMR spectra (**4c**)

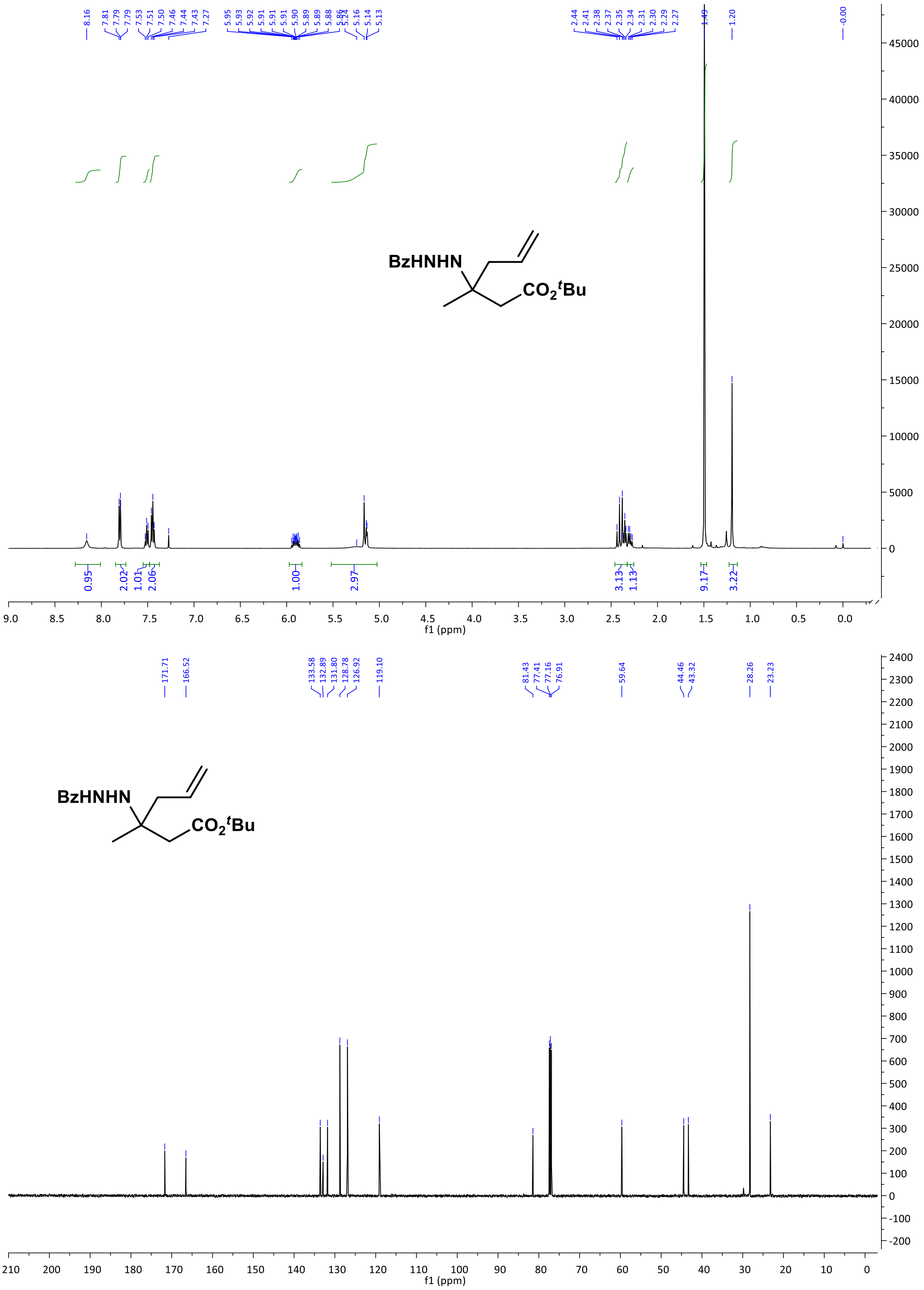


**Supplementary Figure 6.** $^{1}$H and $^{13}$C NMR spectra (**4d**)

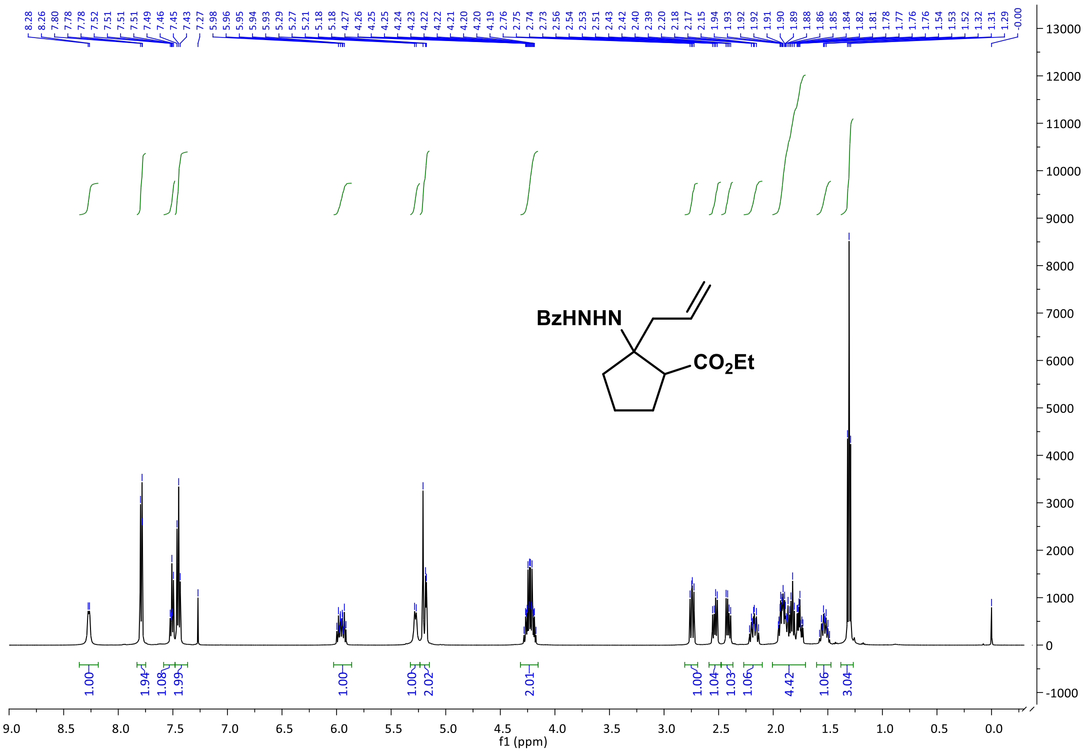


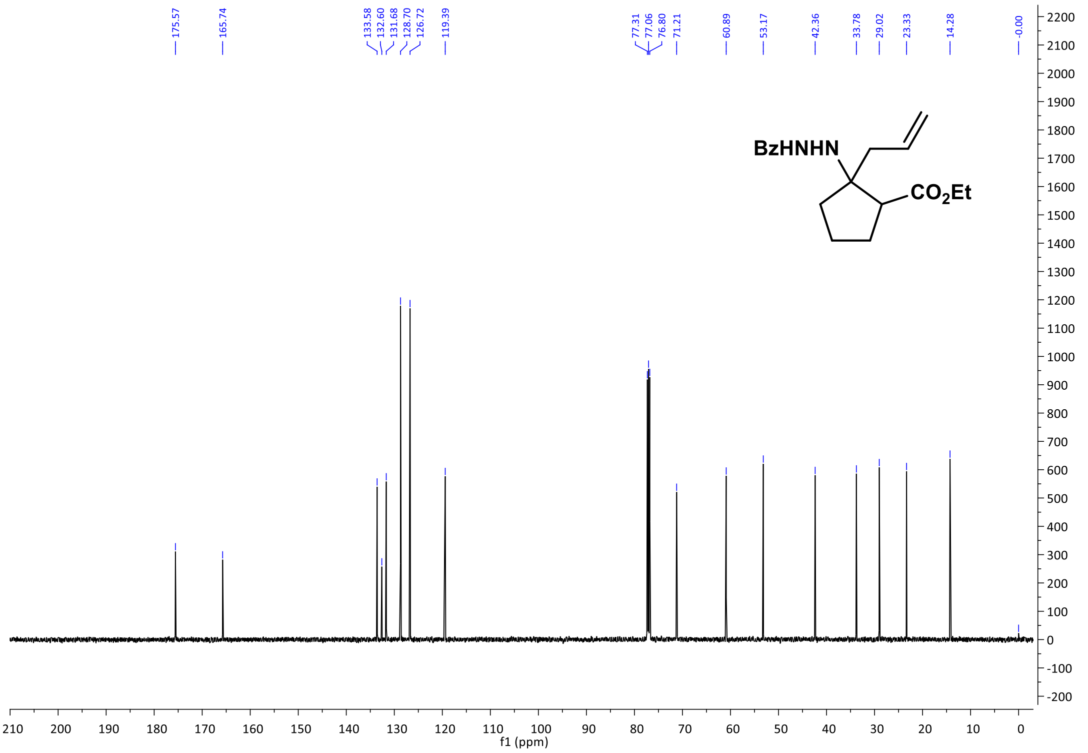


**Supplementary Figure 7.** $^{1}$H and $^{13}$C NMR spectra (**4e**)

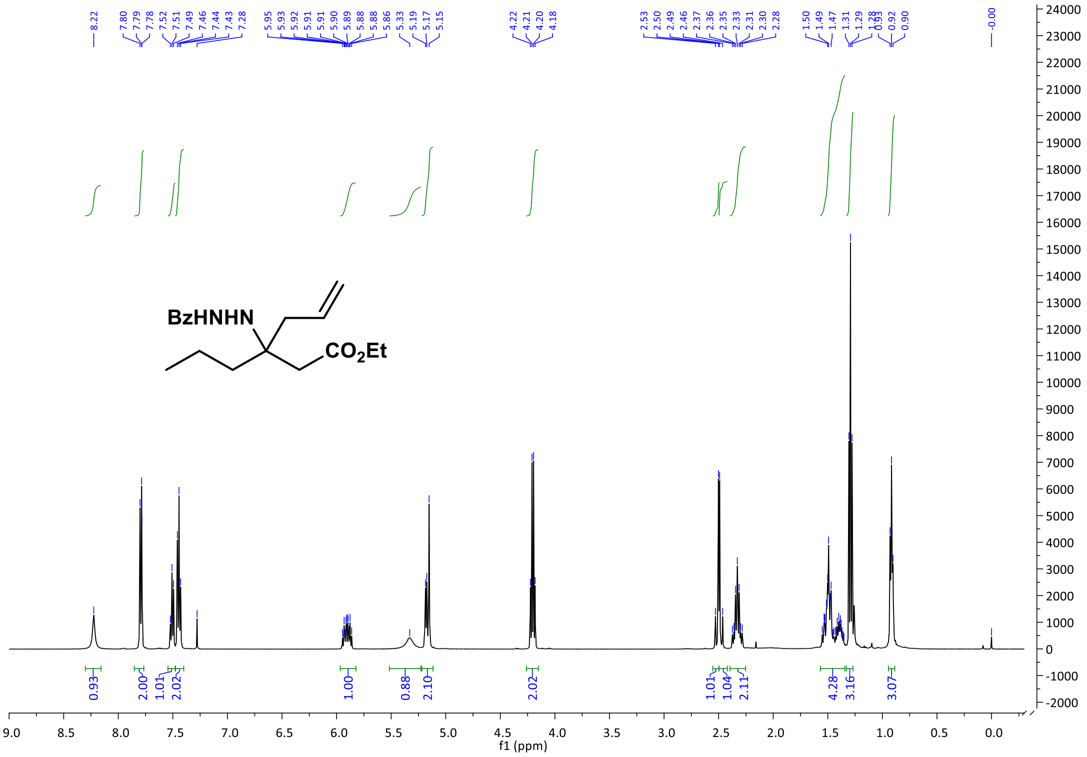


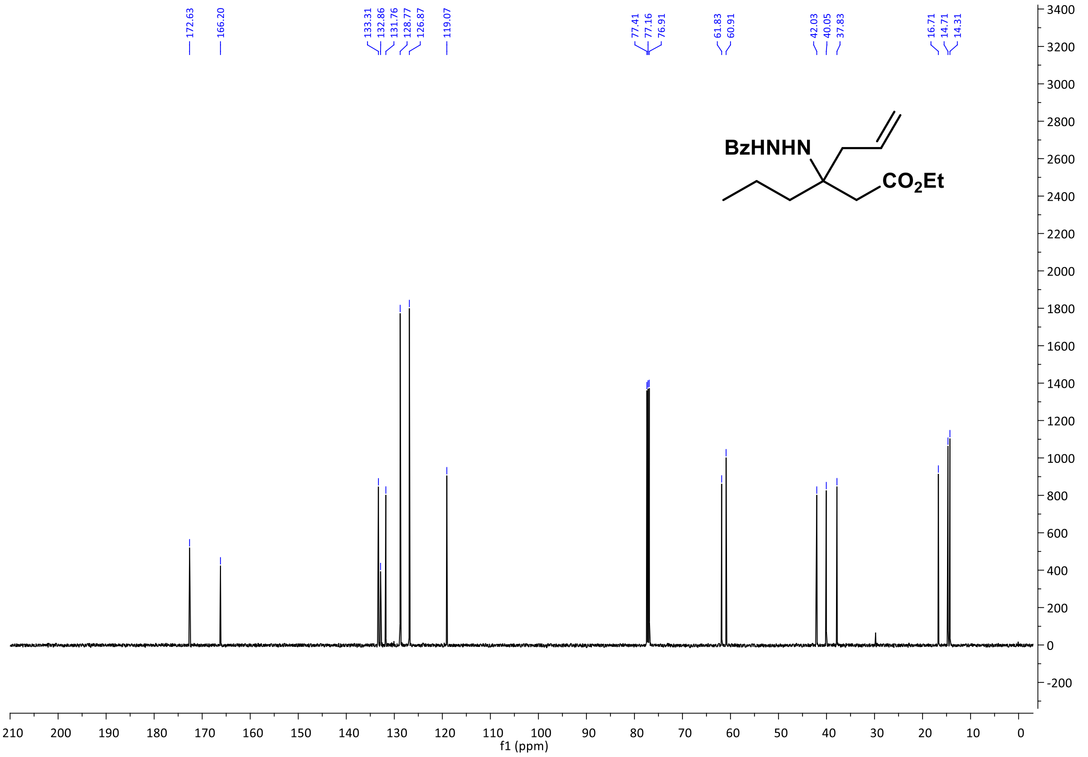


**Supplementary Figure 8.** $^{1}$H and $^{13}$C NMR spectra (**4f**)

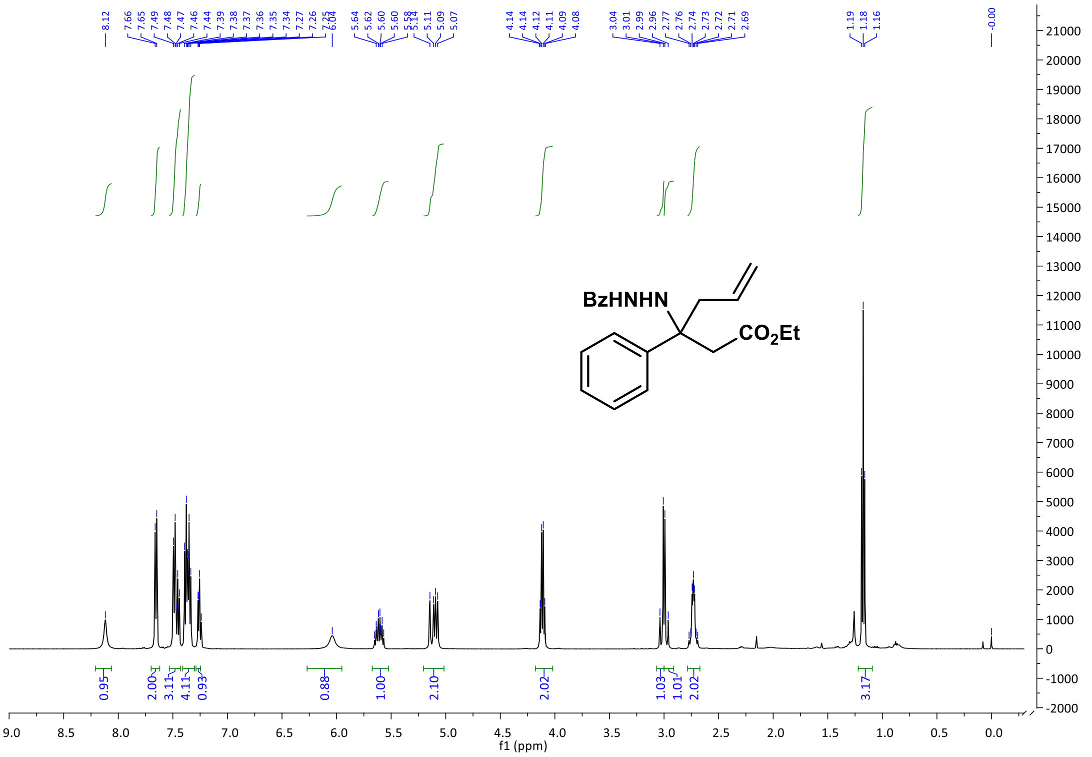

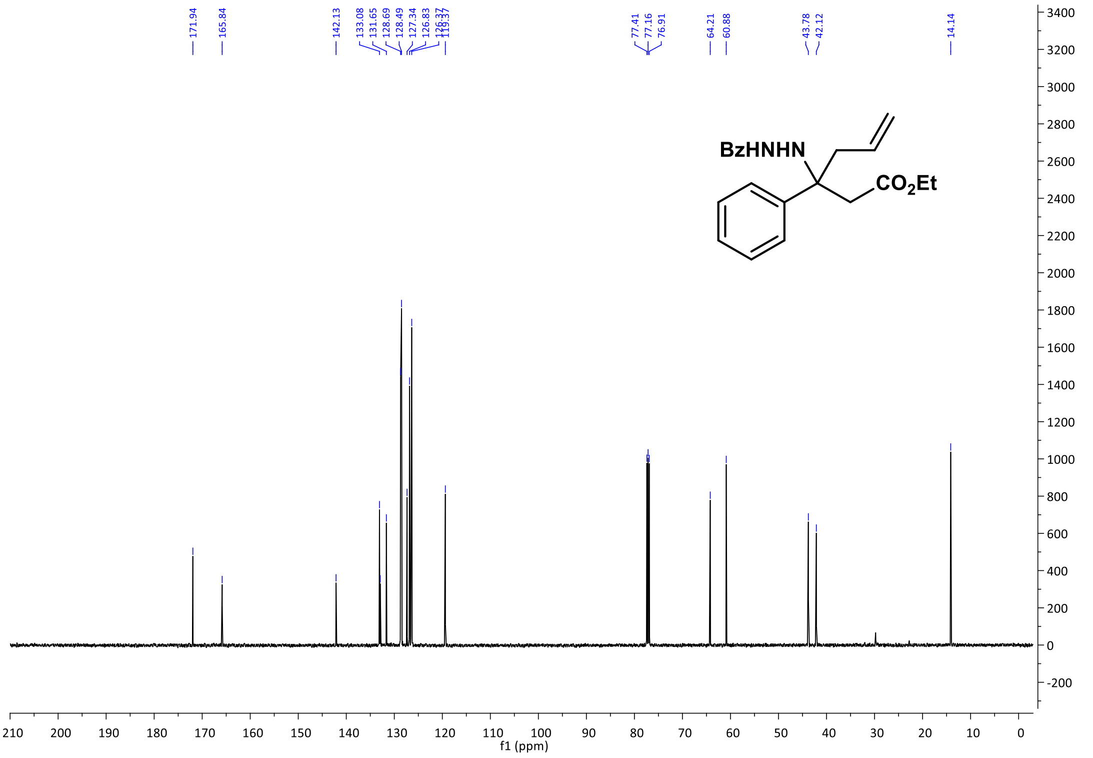

**Supplementary Figure 9.** $^{1}H$ and $^{13}C$ NMR spectra (**4g**)

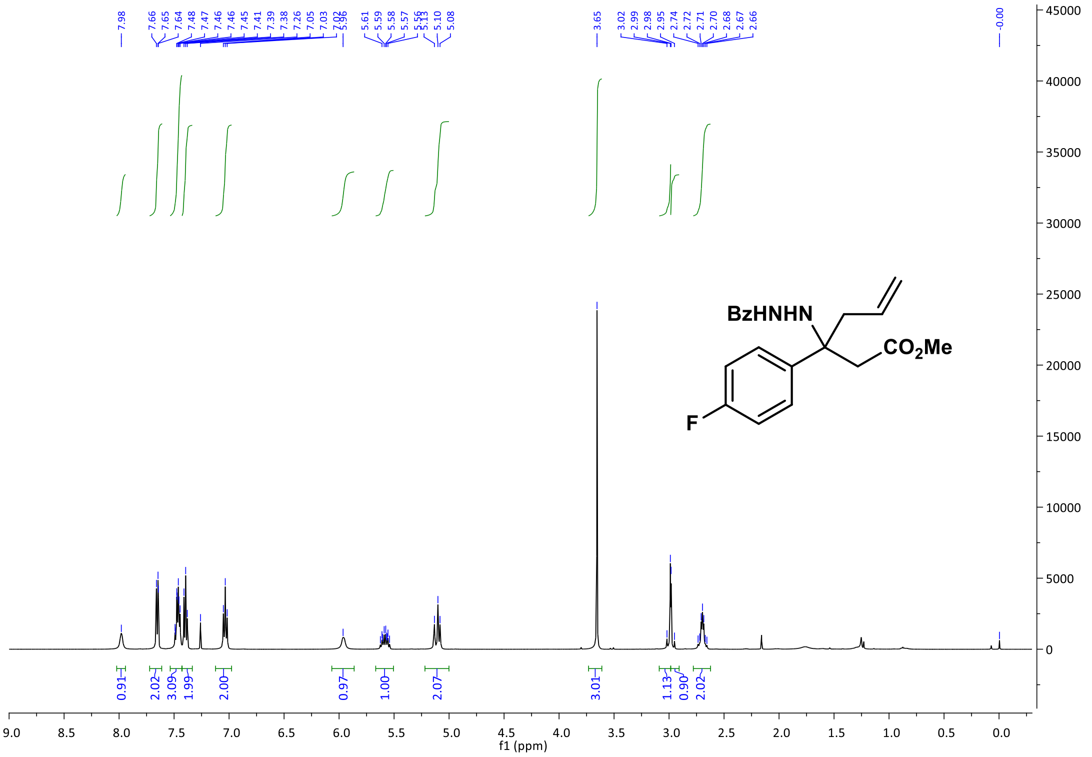


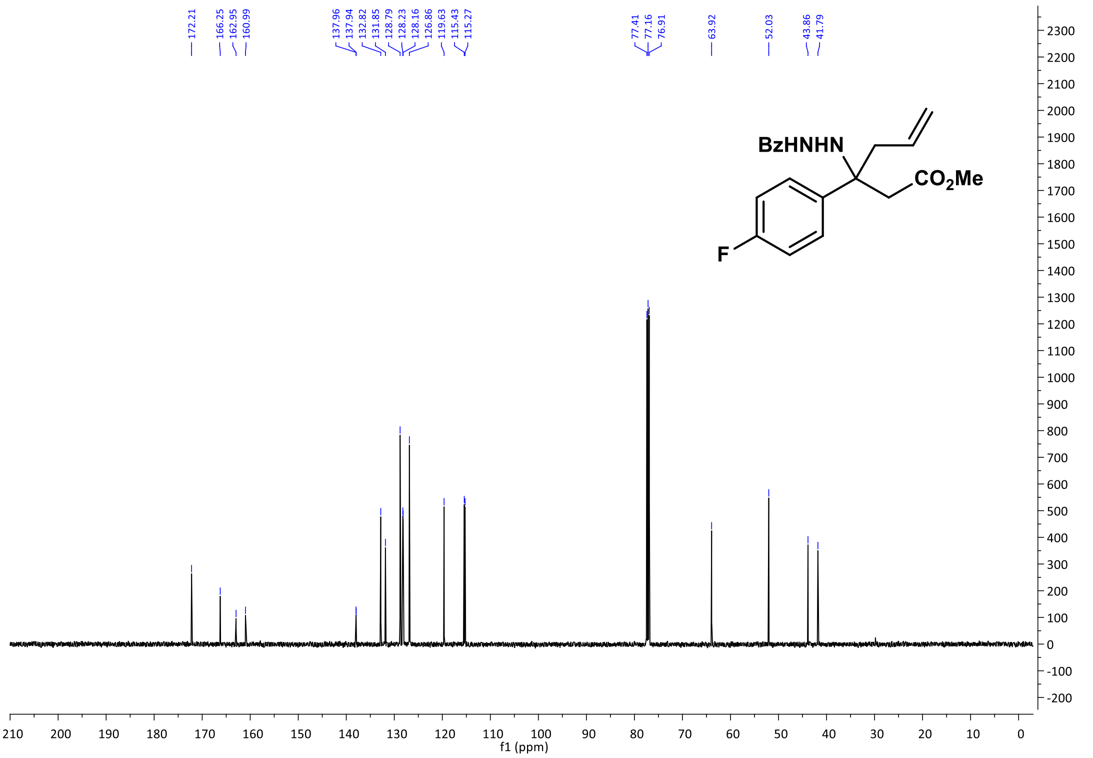


**Supplementary Figure 10.** $^{1}$H and $^{13}$C NMR spectra (**4h**)

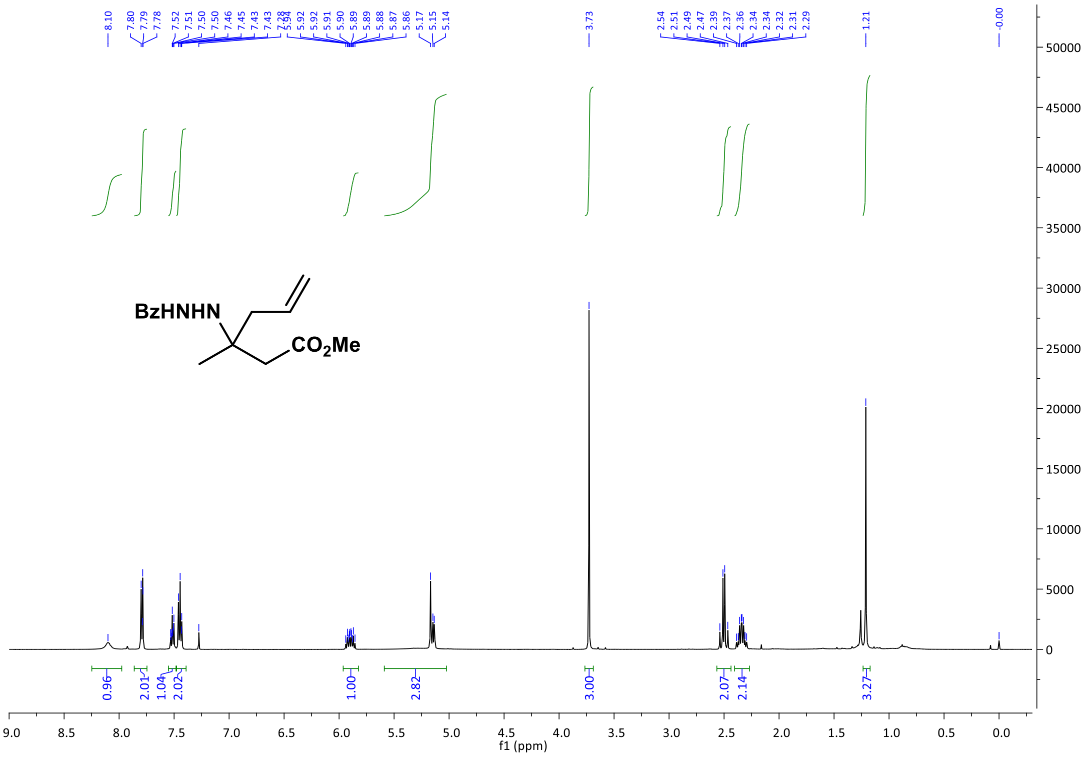


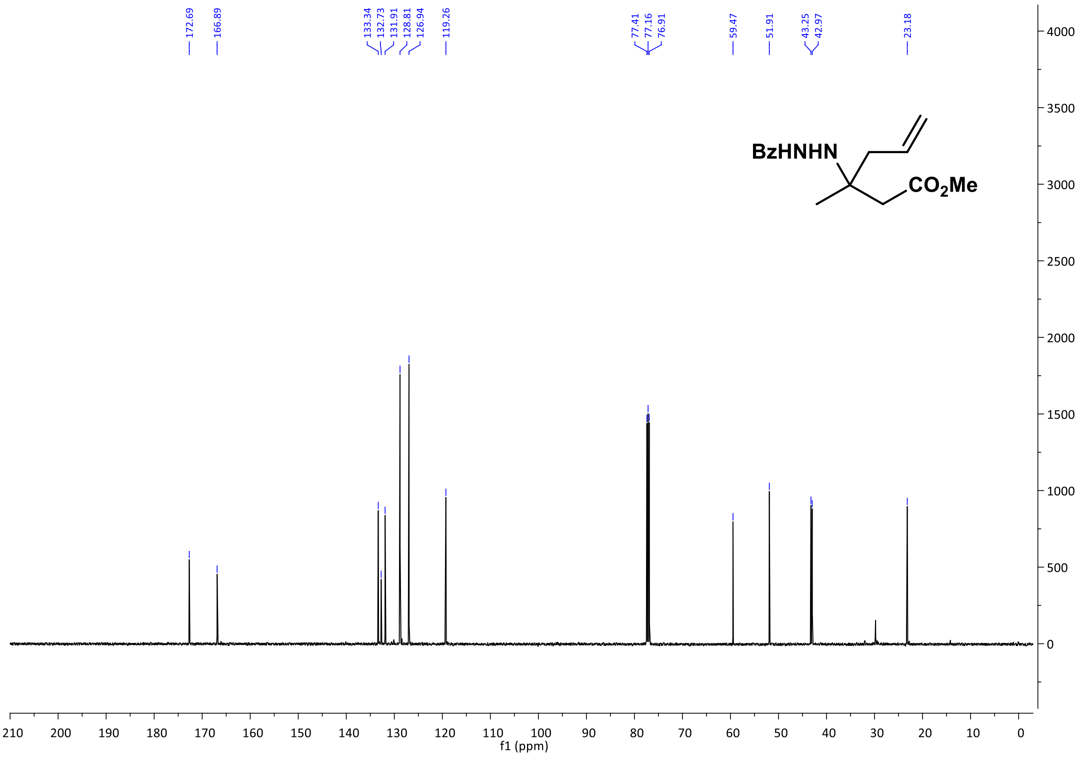


**Supplementary Figure 11.** $^{1}H$ and $^{13}C$ NMR spectra (**4i**)

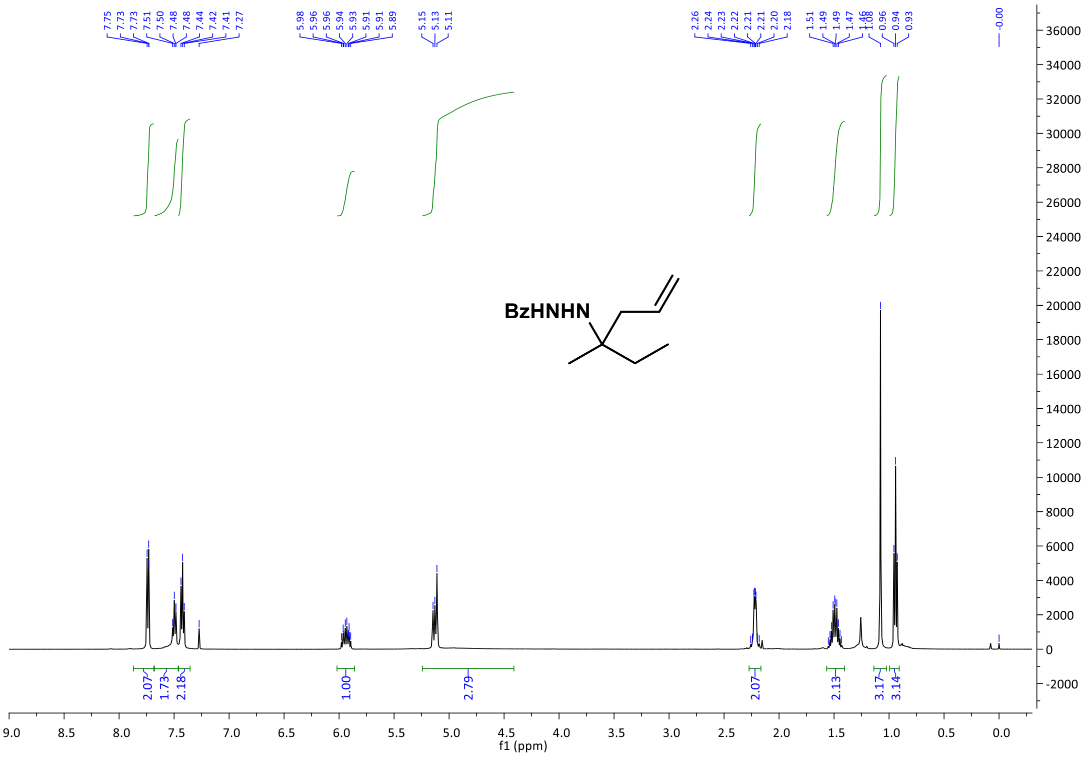


**Supplementary Figure 12.** $^{1}$H NMR spectrum (**11a**)

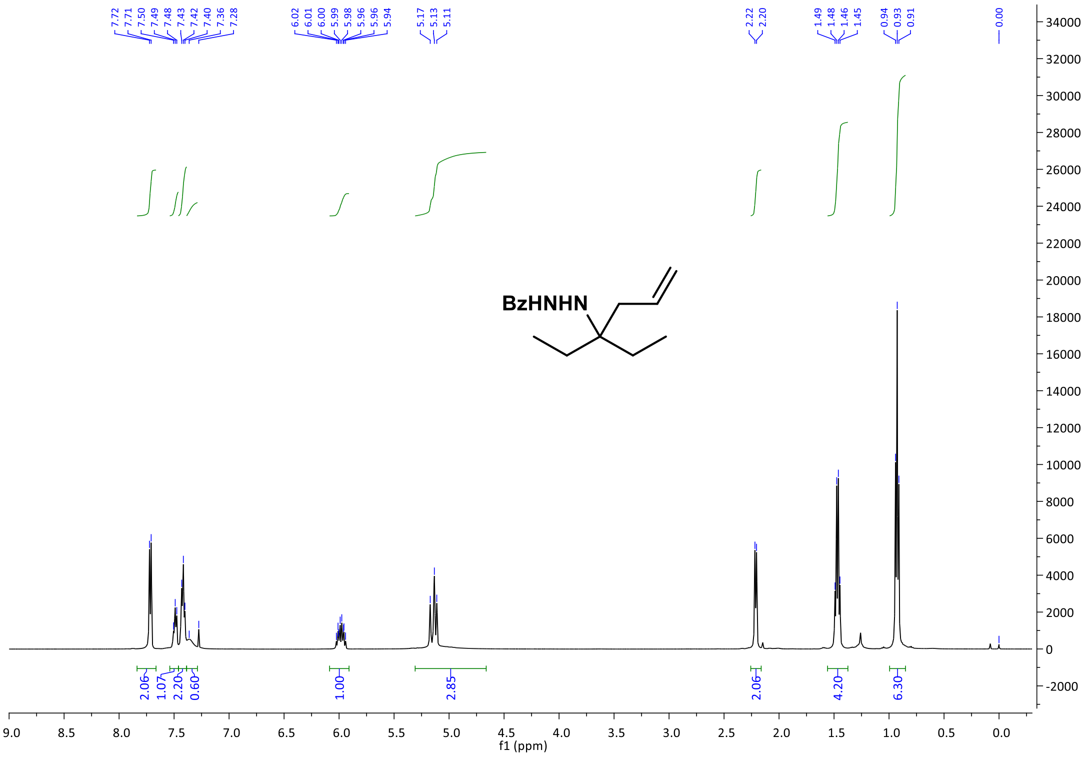


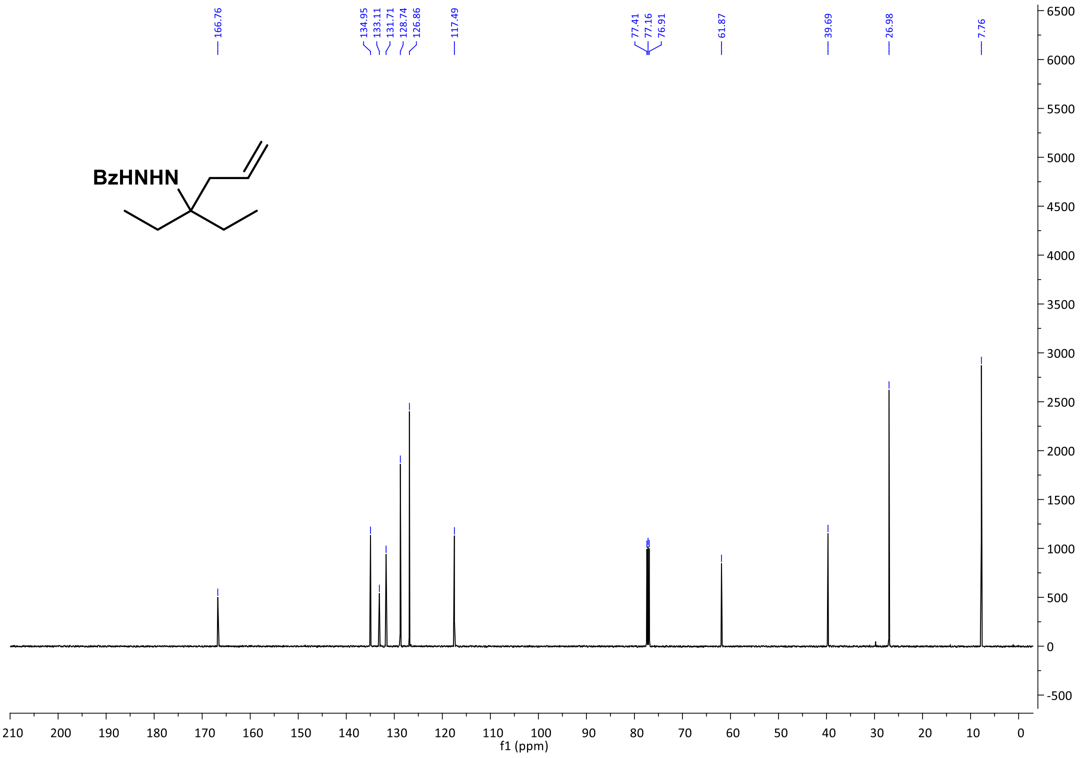


**Supplementary Figure 13.** $^{1}$H and $^{13}$C NMR spectra (**11b**)

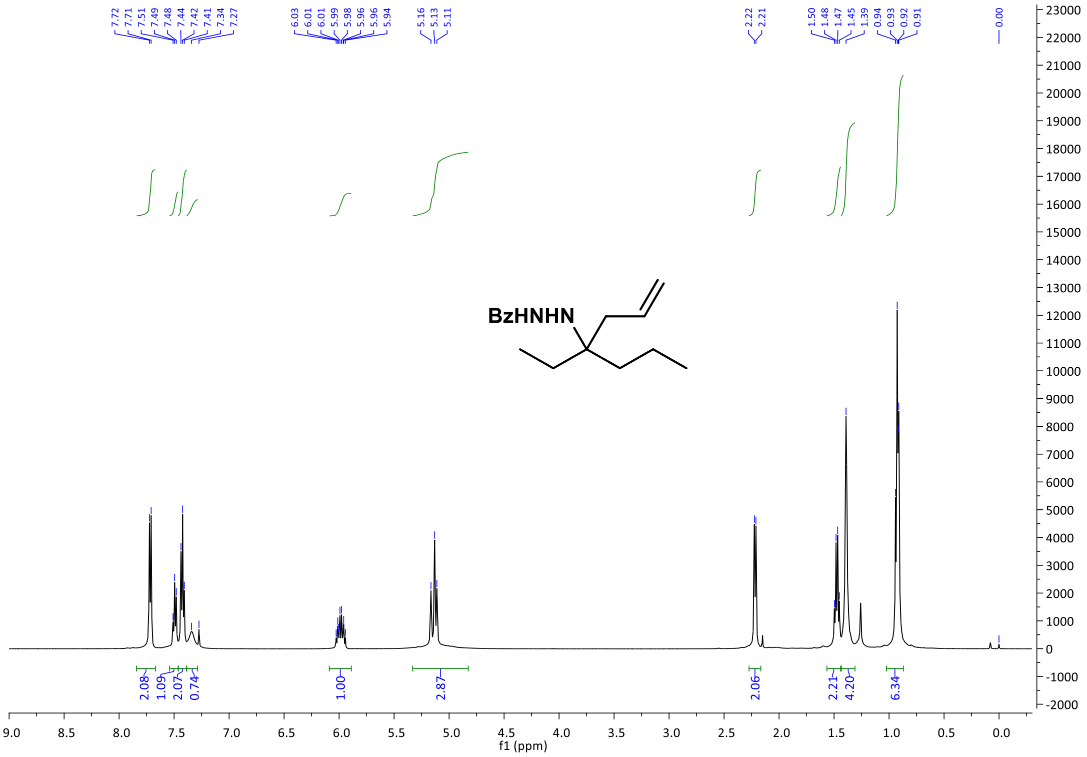


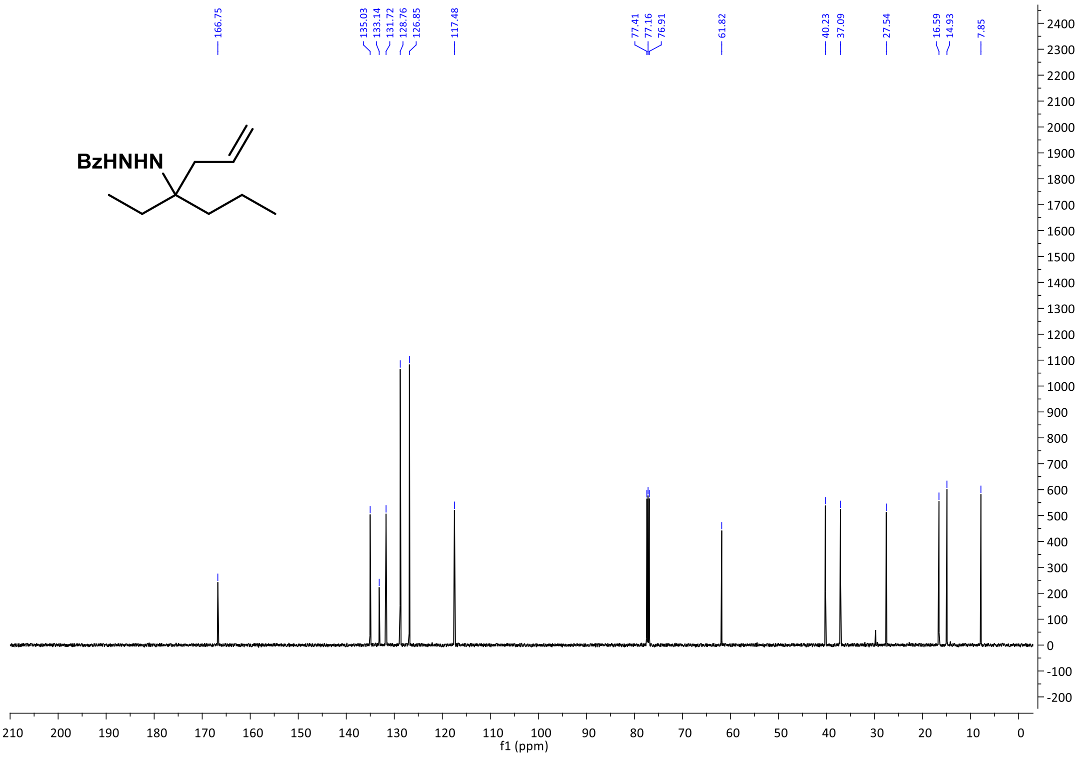


**Supplementary Figure 14.** $^{1}$H and $^{13}$C NMR spectra (**11c**)

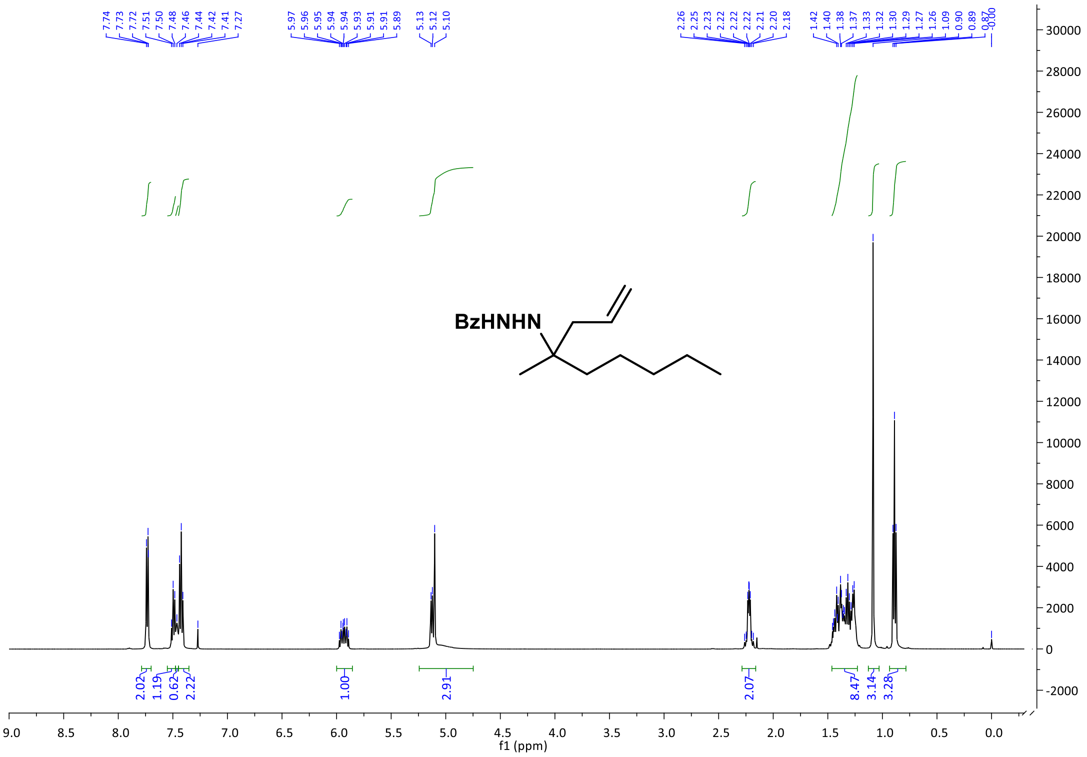


**Supplementary Figure 15.** $^1$H NMR spectrum (**11d**)

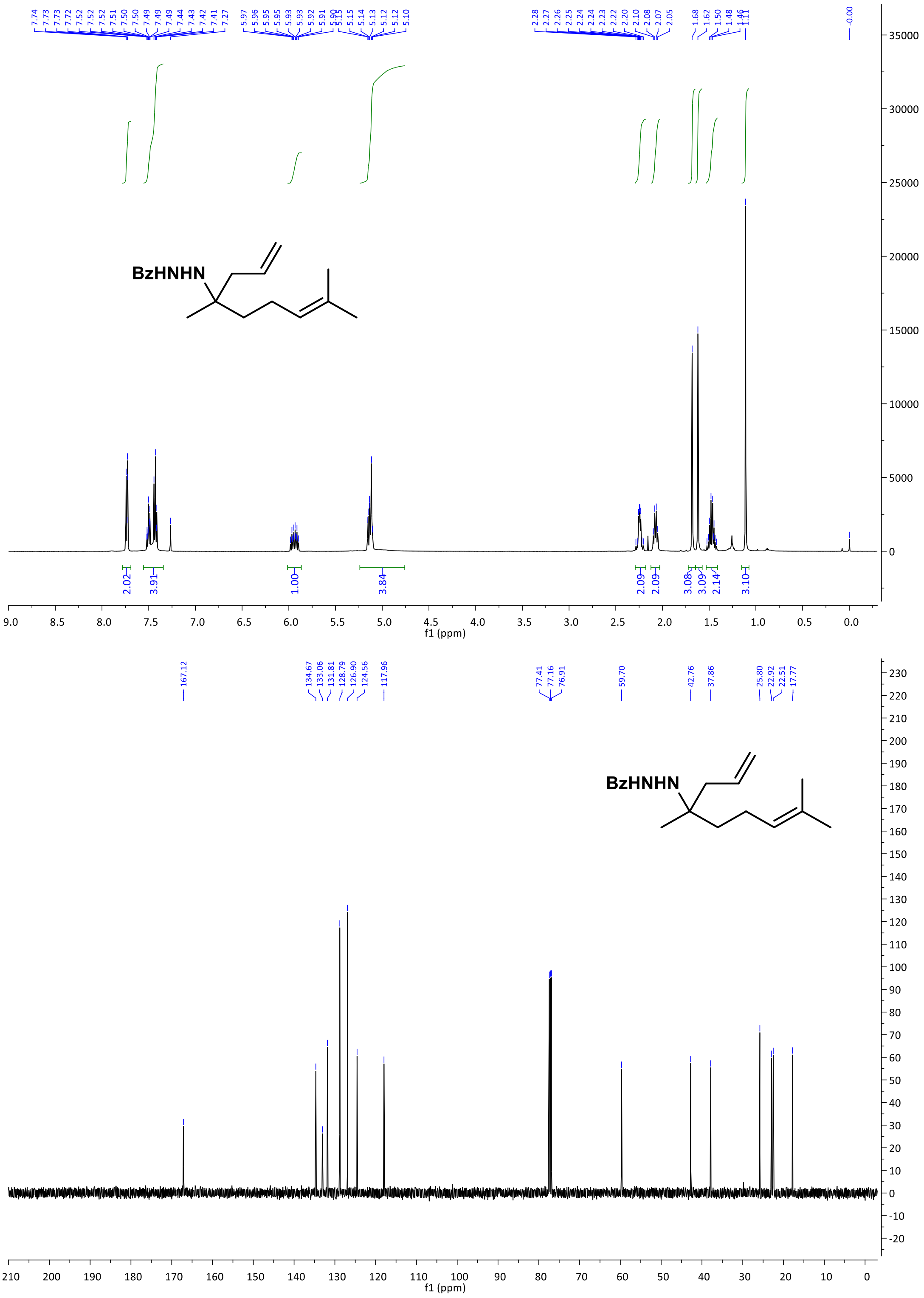

**Supplementary Figure 16.** $^{1}H$ and $^{13}C$ NMR spectra (**11e**)

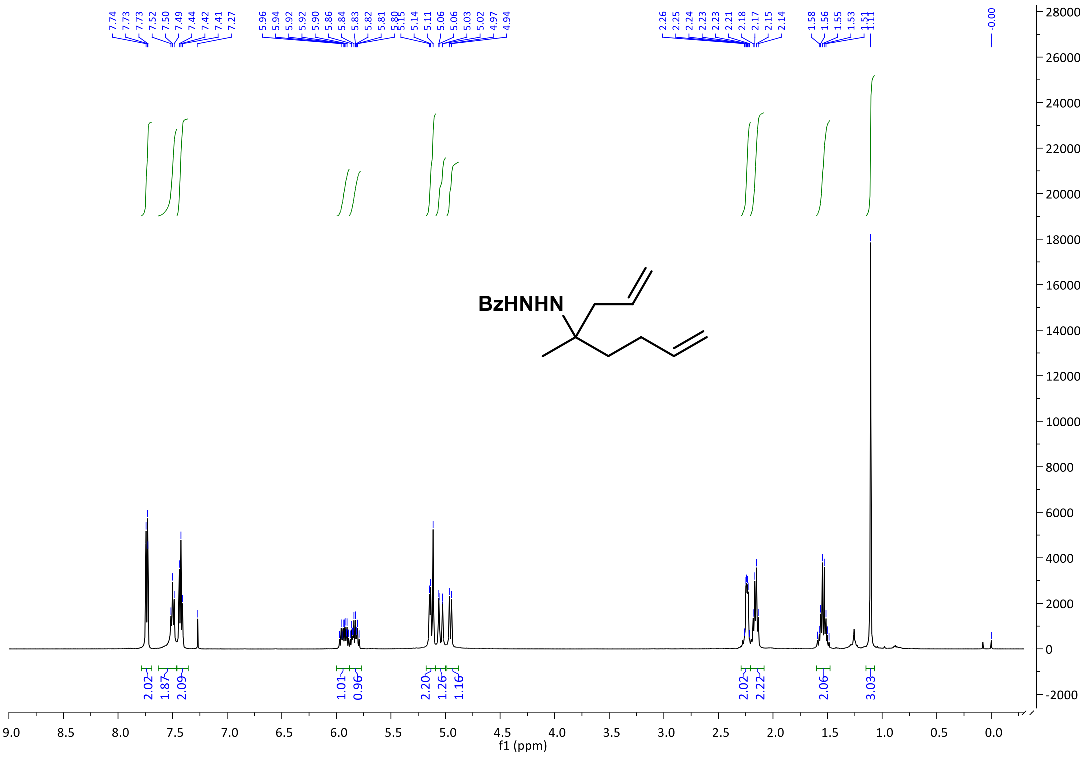


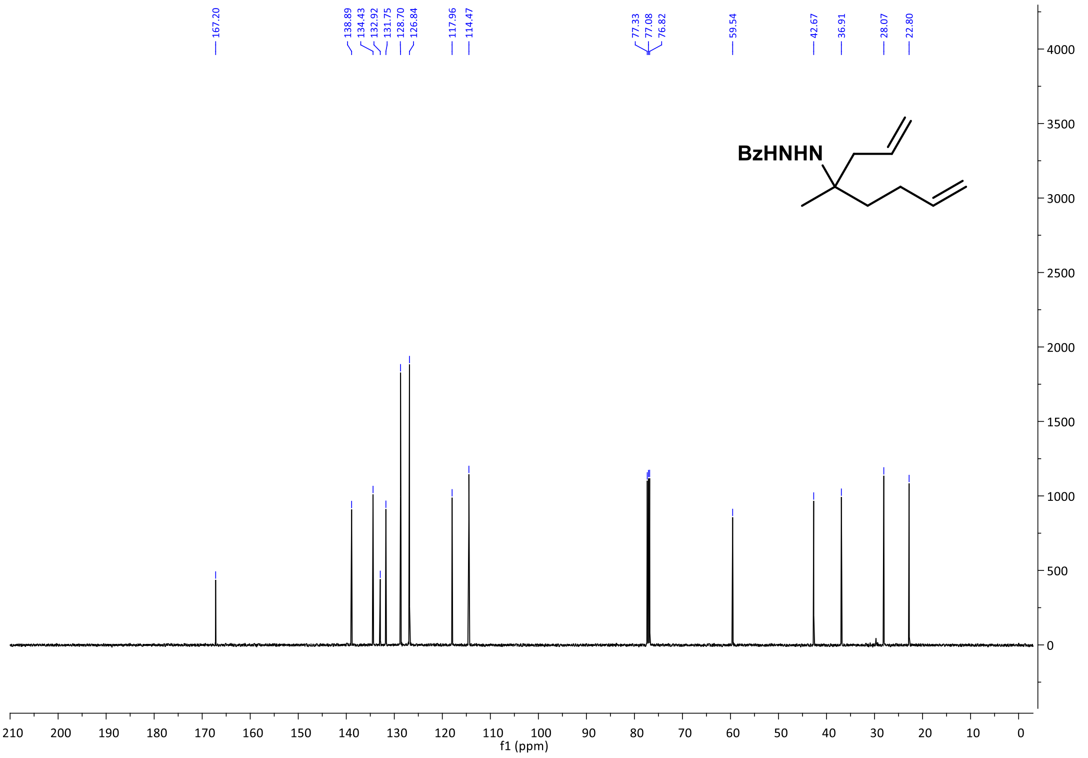


**Supplementary Figure 17.** $^{1}$H and $^{13}$C NMR spectra (**11f**)

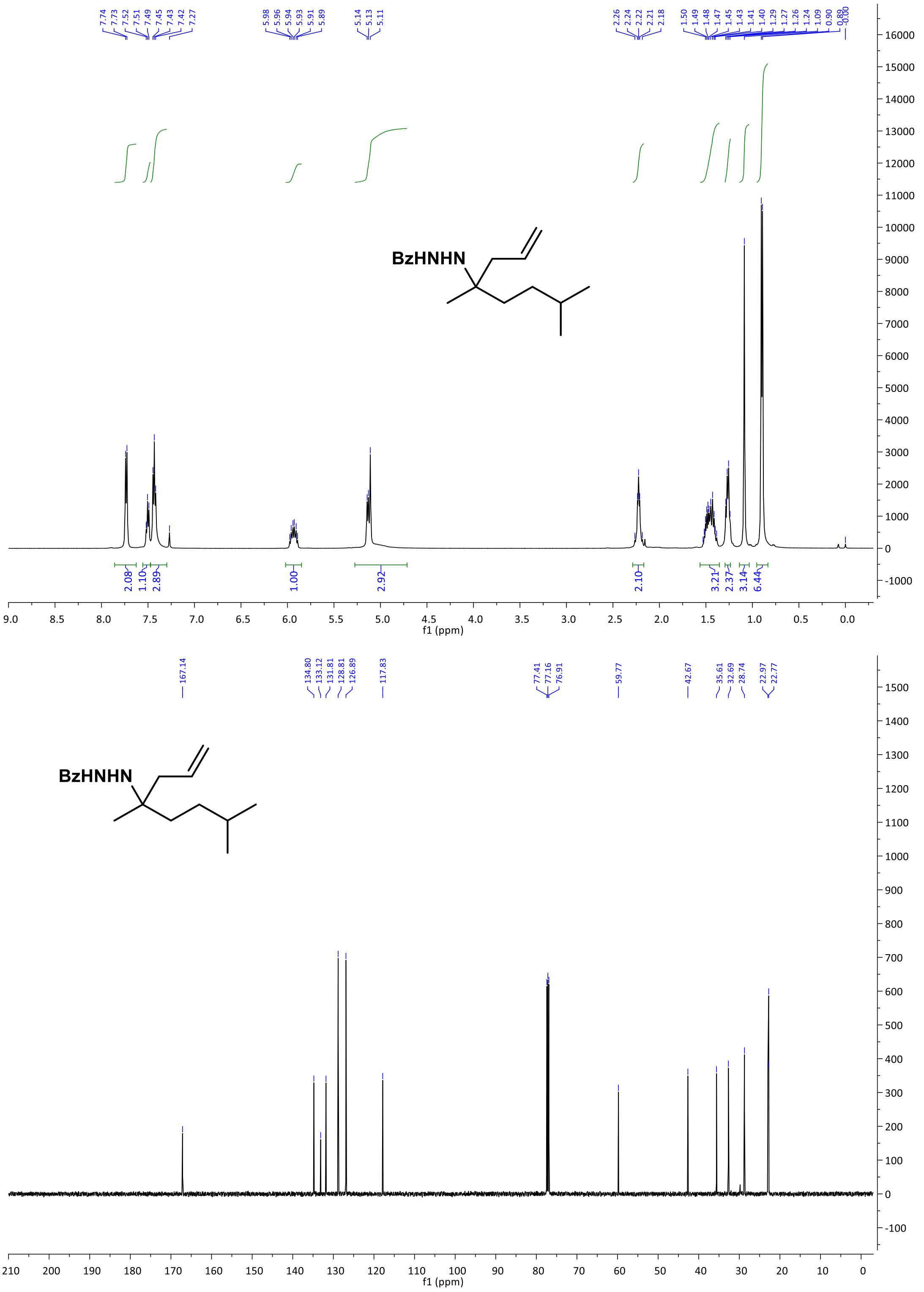


**Supplementary Figure 18.** $^{1}H$ and $^{13}C$ NMR spectra (**11g**)

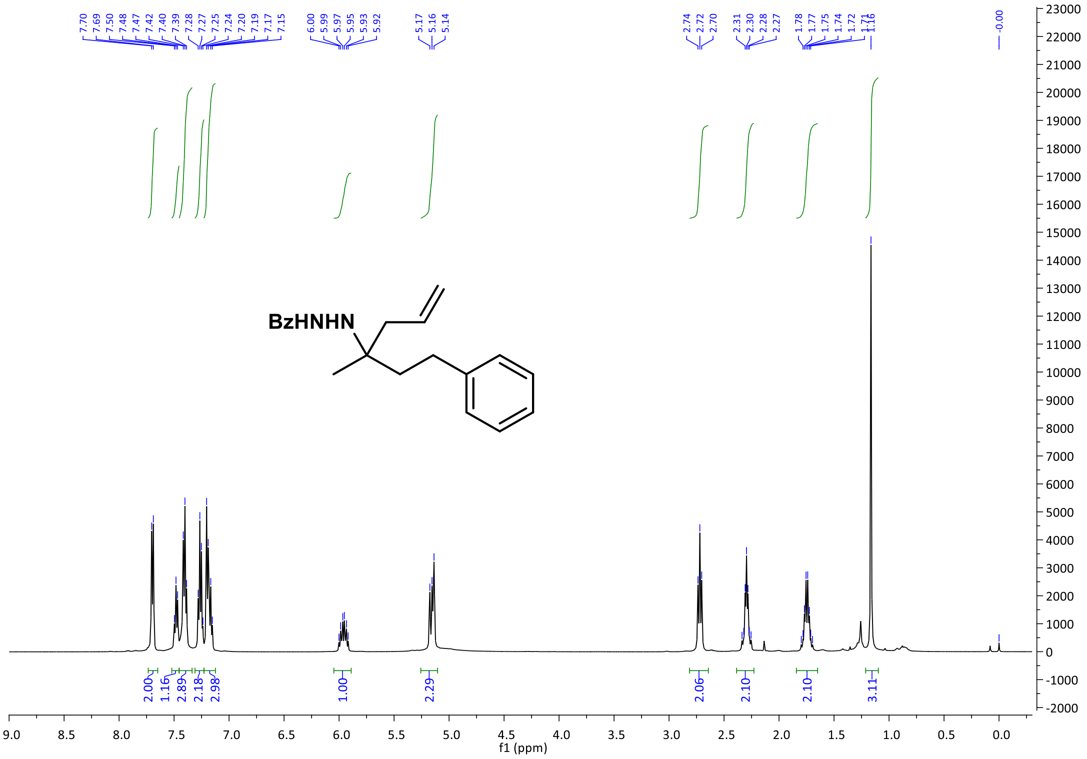

**Supplementary Figure 19.** $^{1}$H NMR spectrum (**11h**)

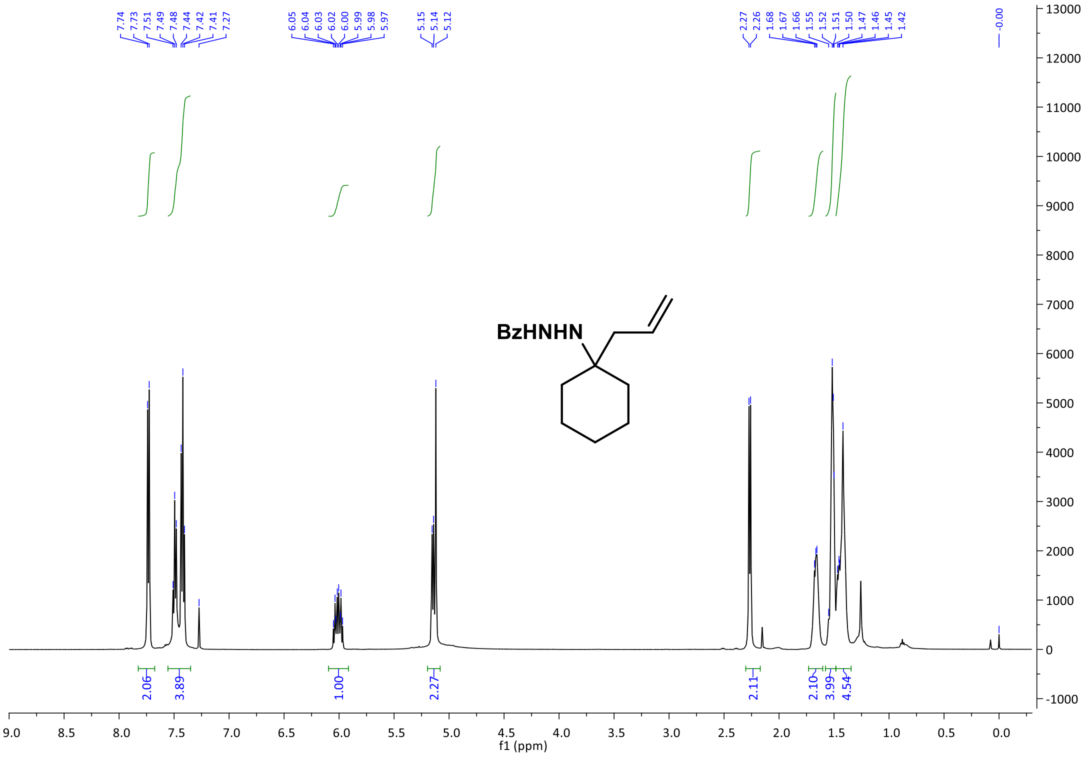


**Supplementary Figure 20.** $^{1}$H NMR spectrum (**11i**)

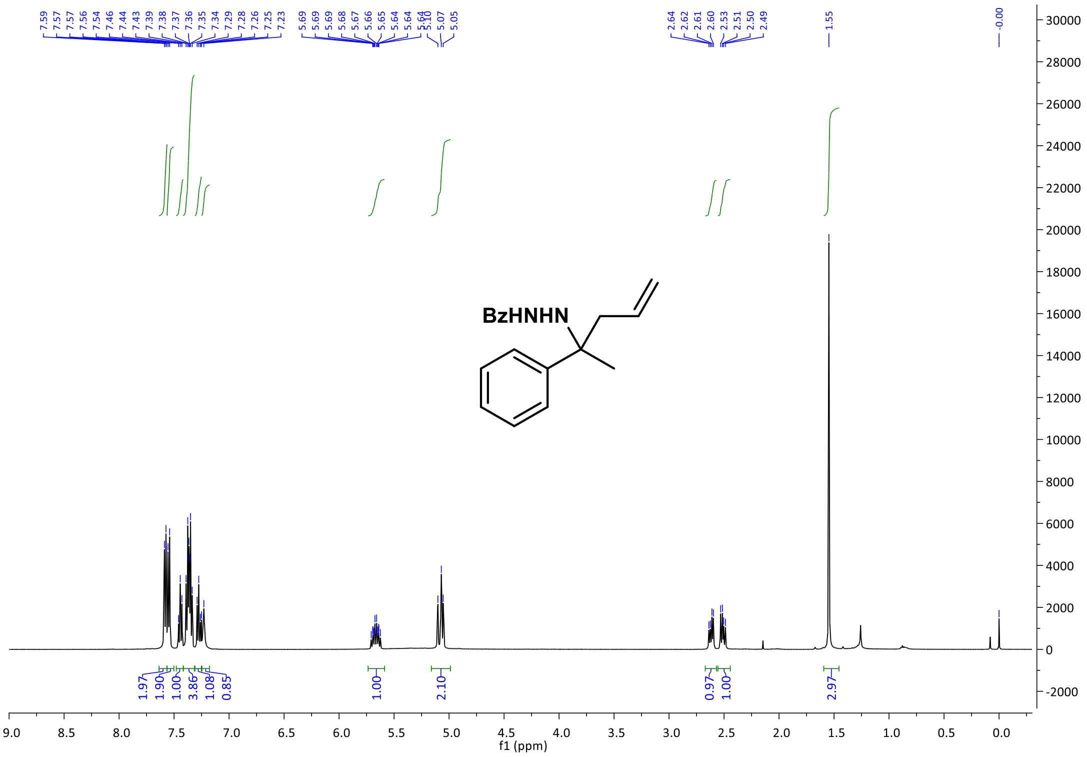


**Supplementary Figure 21.** $^{1}$H NMR spectrum (**15a**)

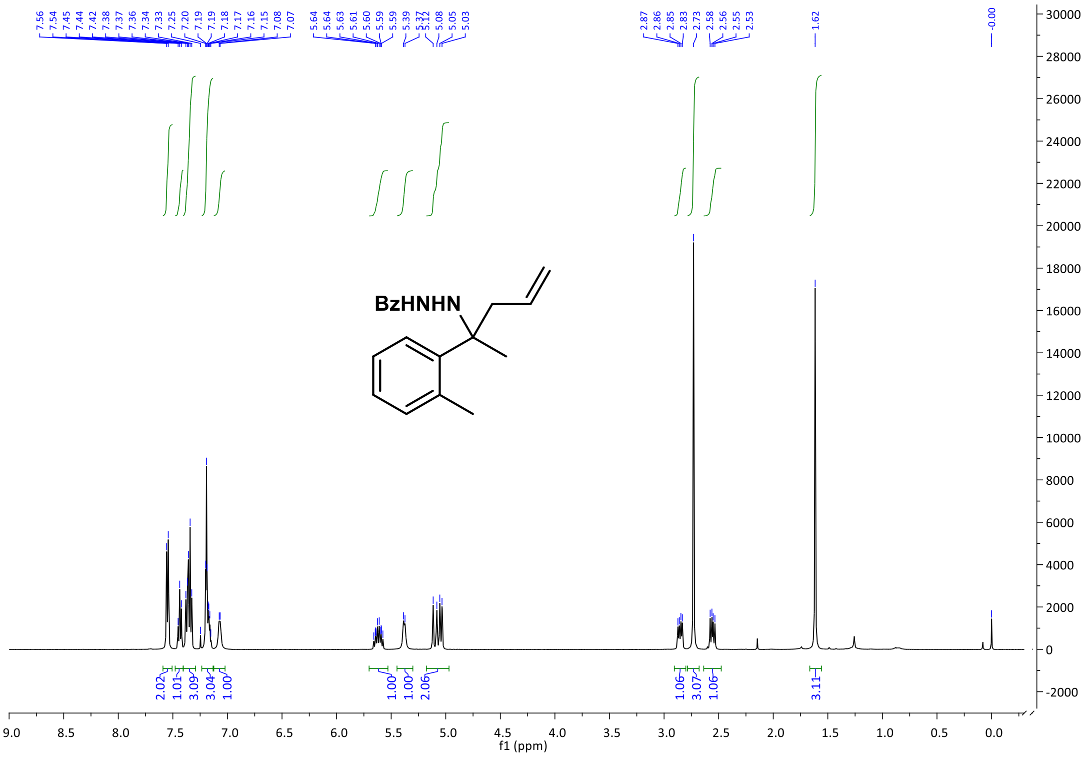


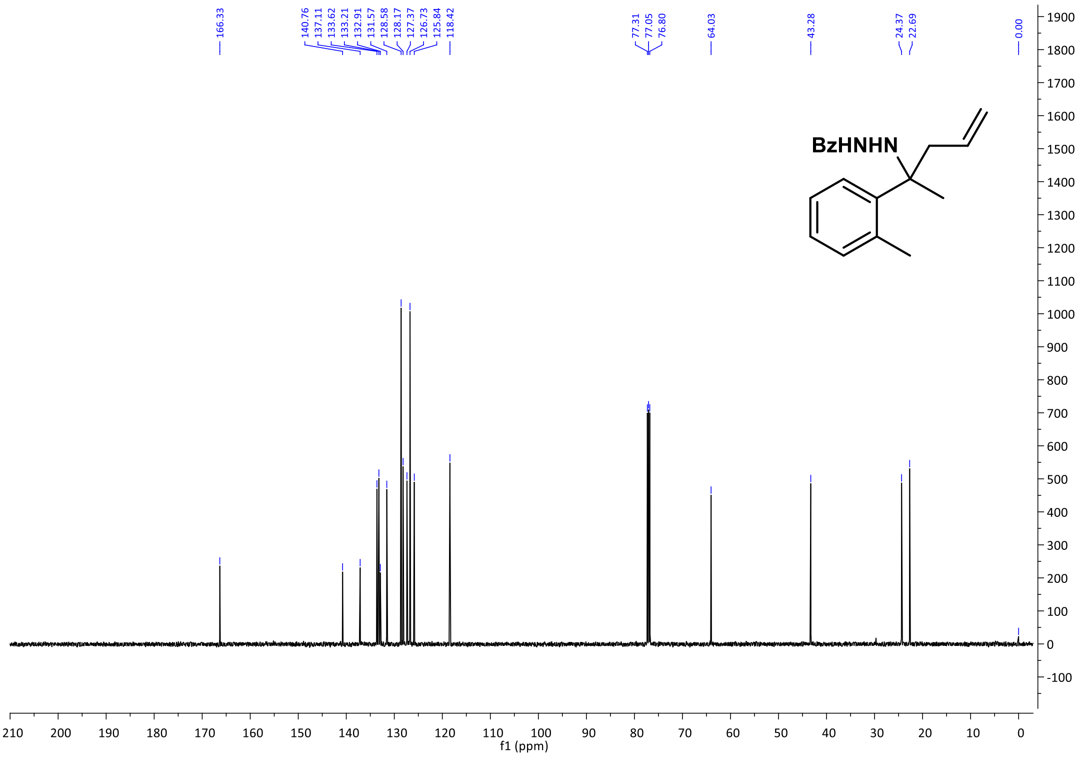


**Supplementary Figure 22.** $^{1}H$ and $^{13}C$ NMR spectra (**15b**)

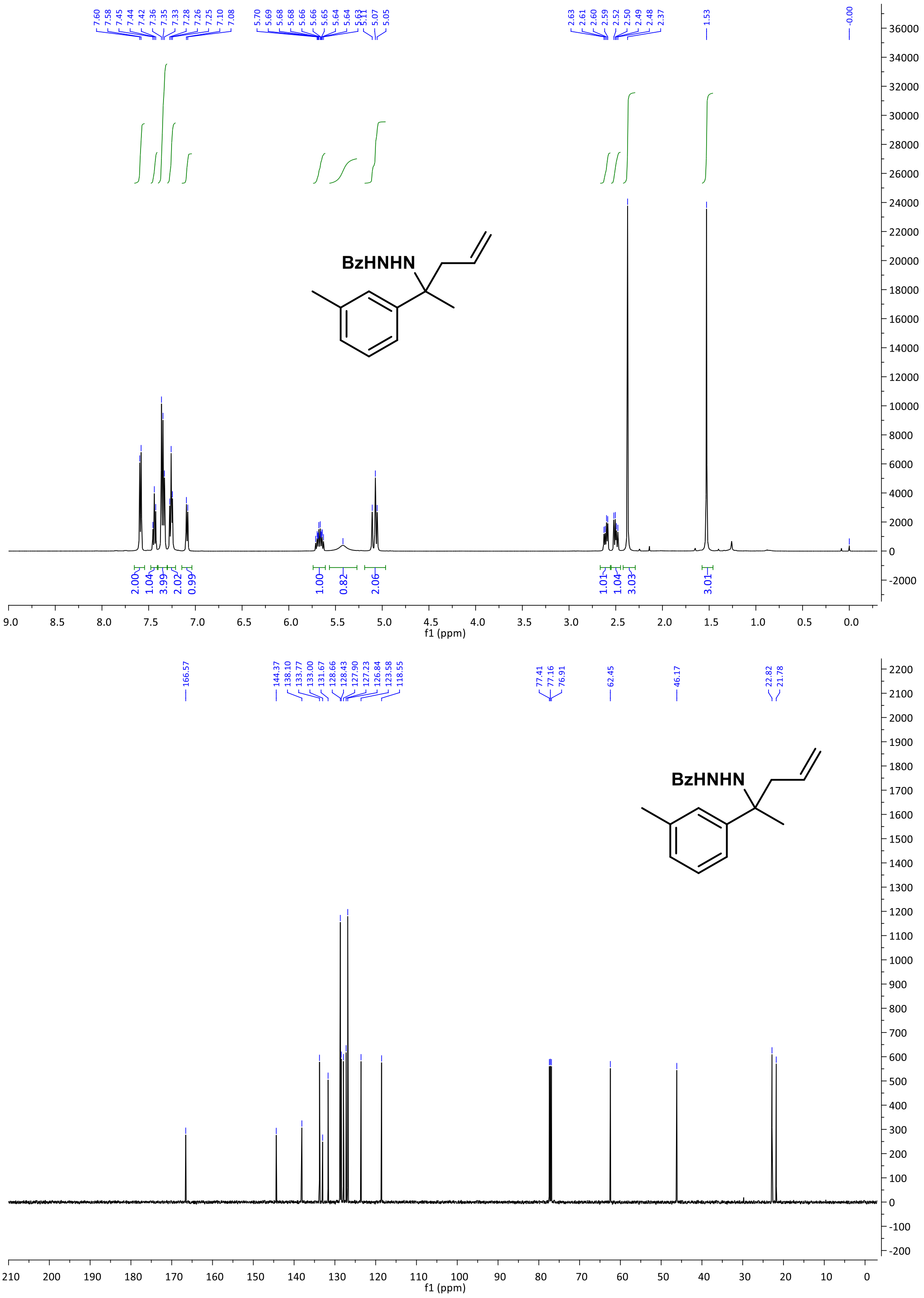

**Supplementary Figure 23.** $^{1}H$ and $^{13}C$ NMR spectra (**15c**)

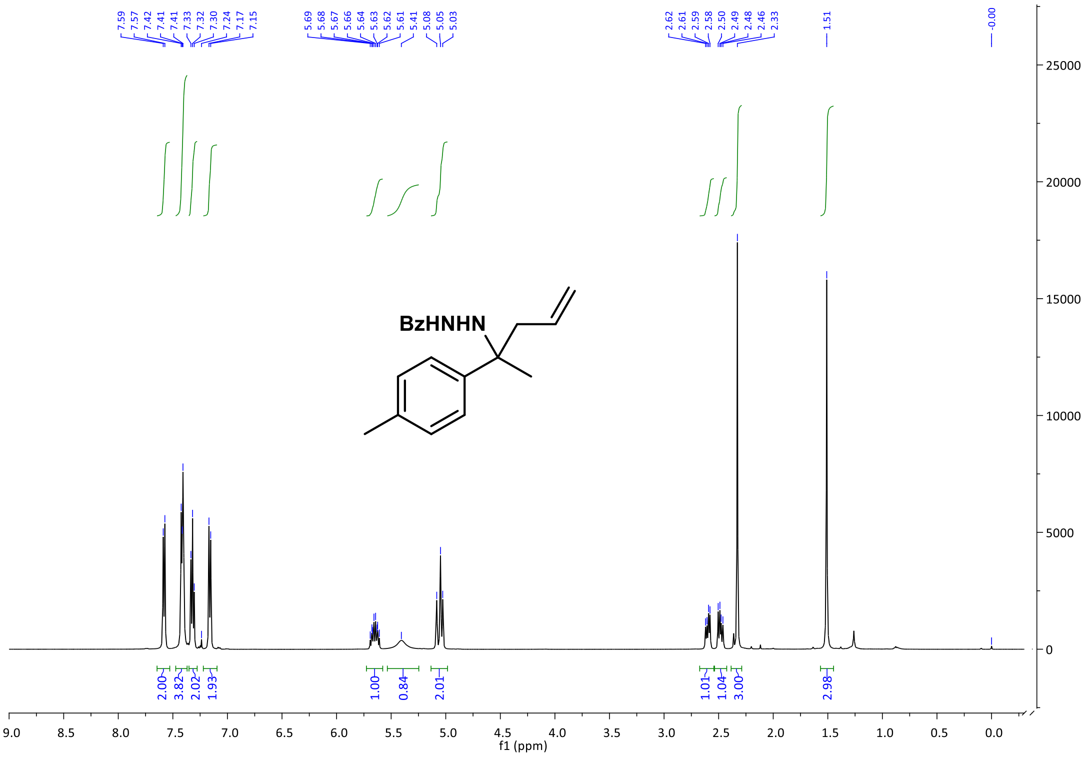

**Supplementary Figure 24.** $^{1}$H NMR spectrum (**15d**)

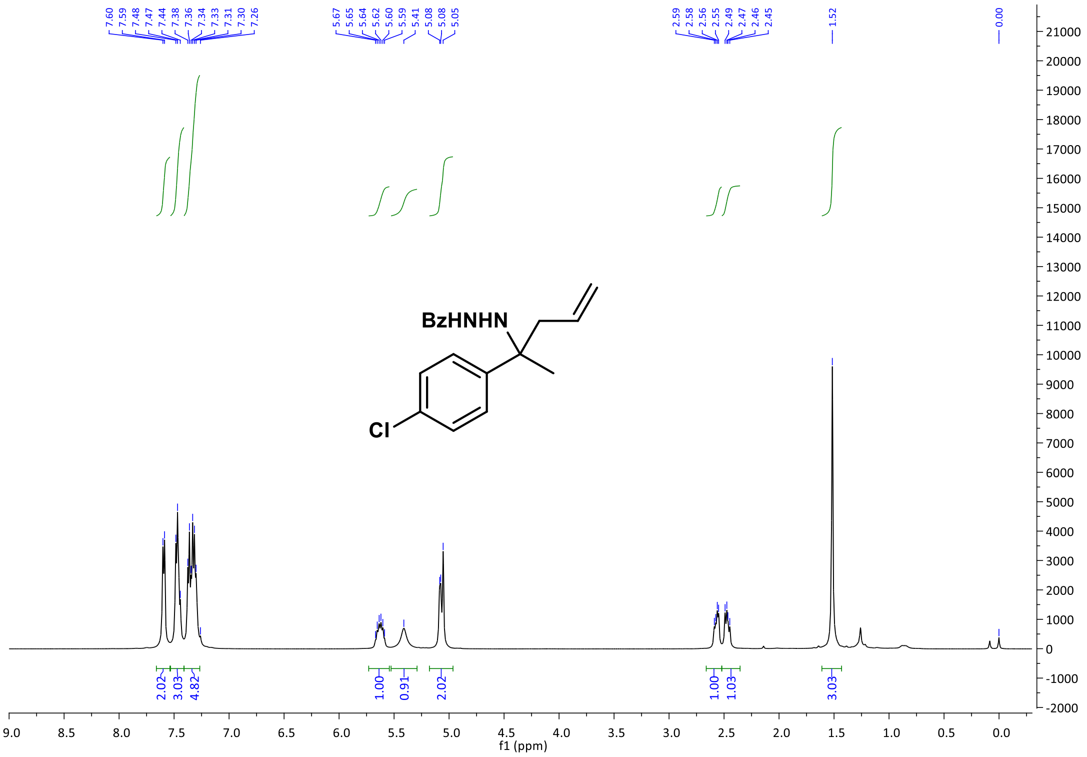


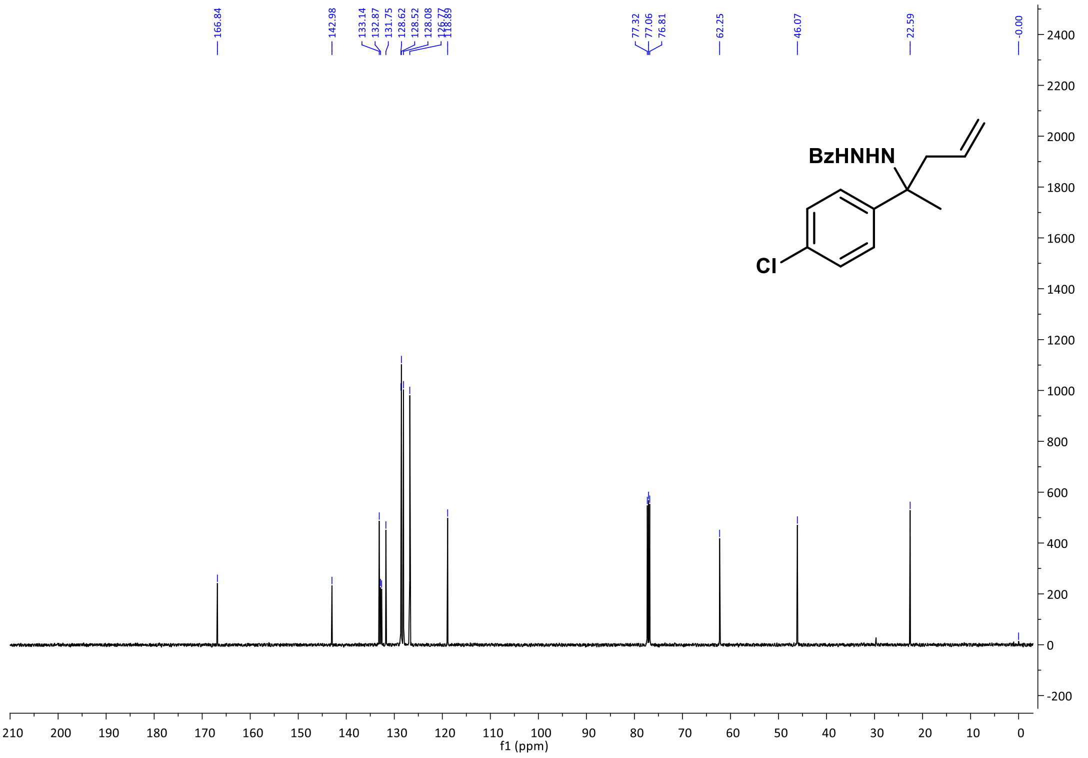


**Supplementary Figure 25.** $^{1}H$ and $^{13}C$ NMR spectra (**15e**)

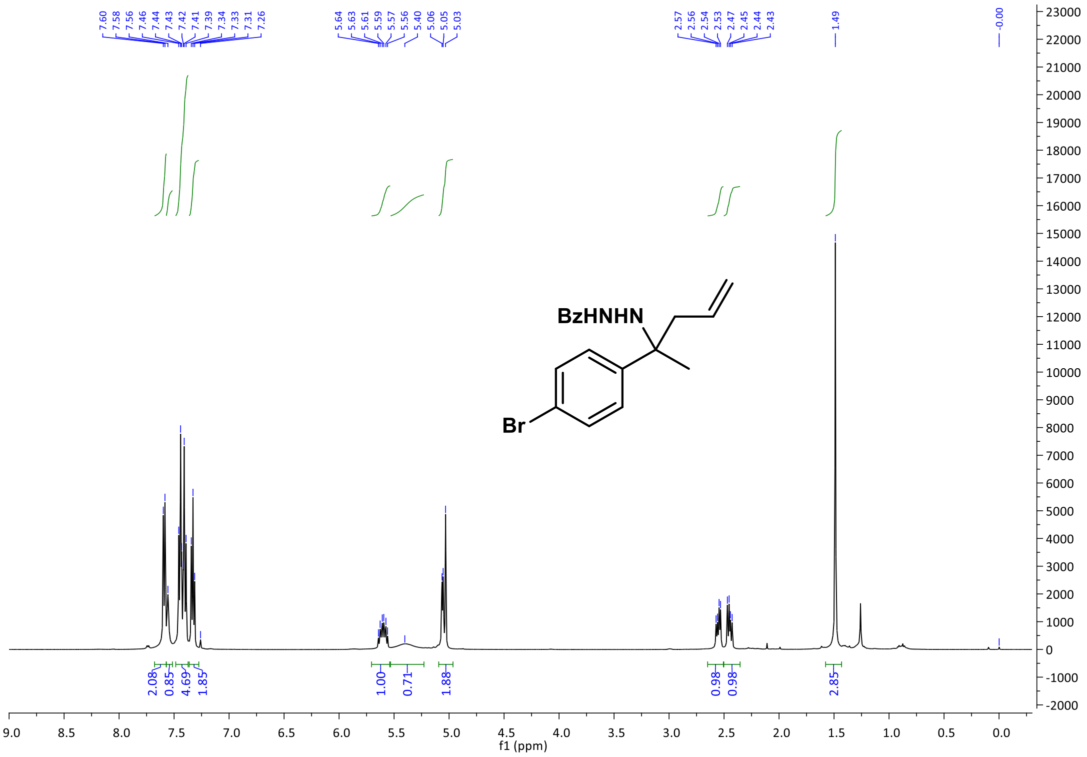


**Supplementary Figure 26.** $^{1}$H NMR spectrum (**15f**)

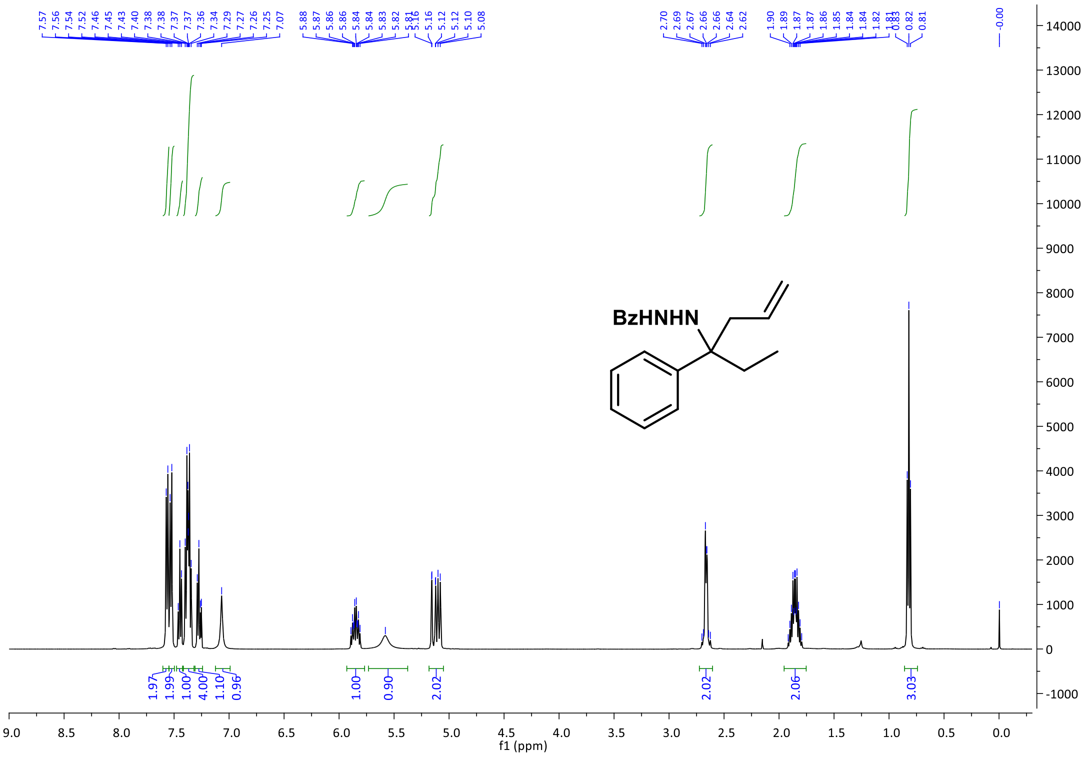


**Supplementary Figure 27.** $^{1}$H NMR spectrum (**15g**)

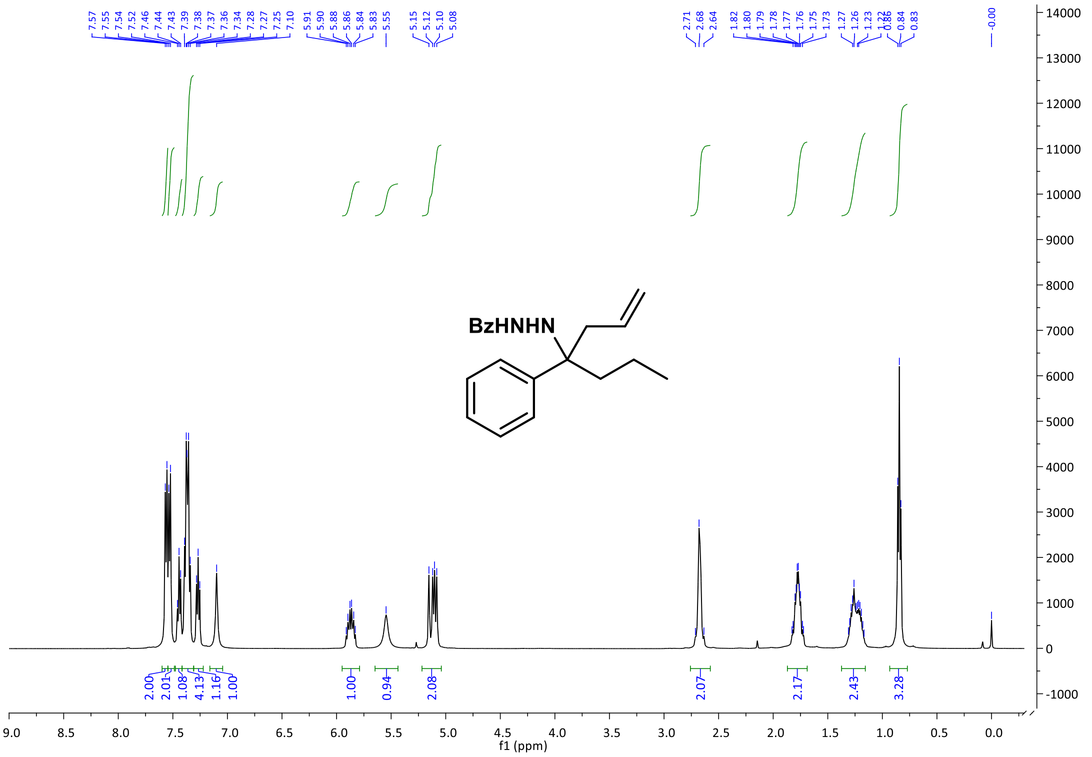

**Supplementary Figure 28.** $^{1}$H NMR spectrum (**15h**)

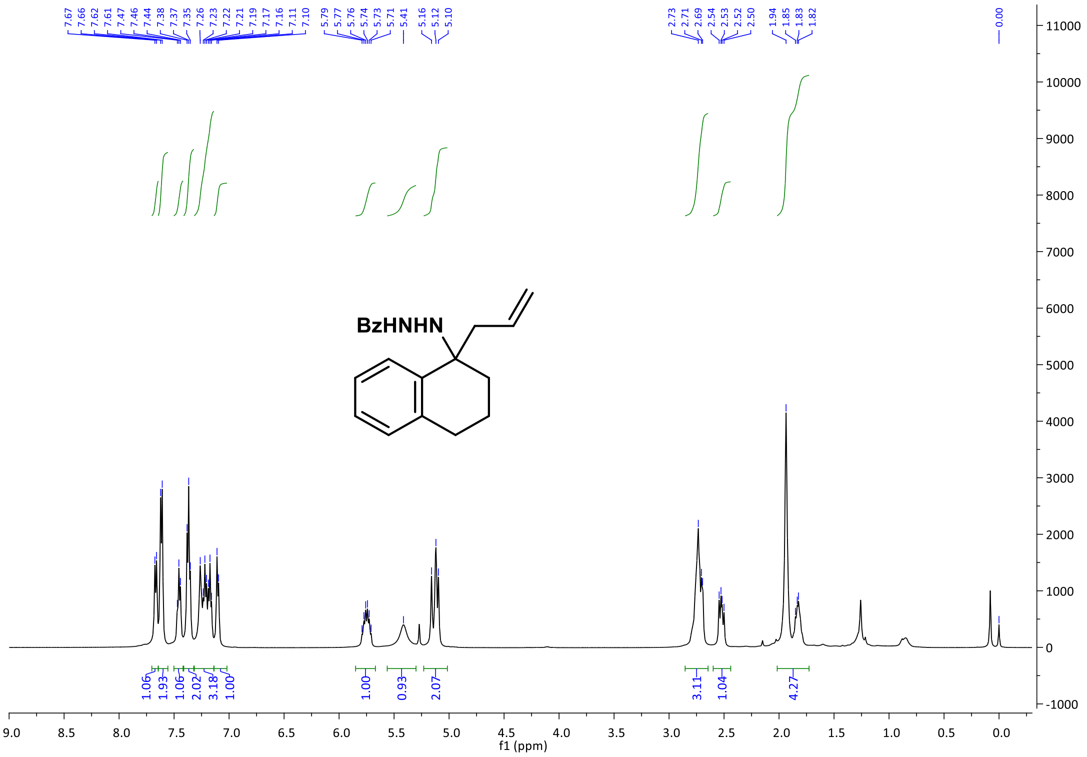


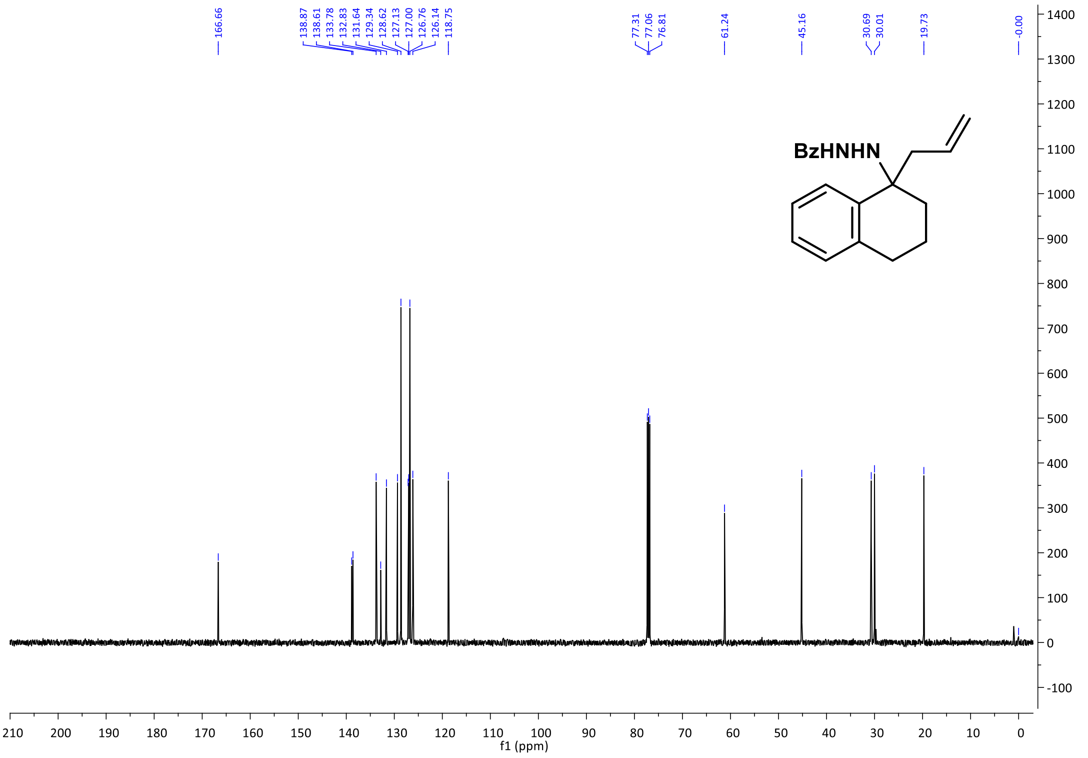


**Supplementary Figure 29.** $^{1}$H and $^{13}$C NMR spectra (**15i**)

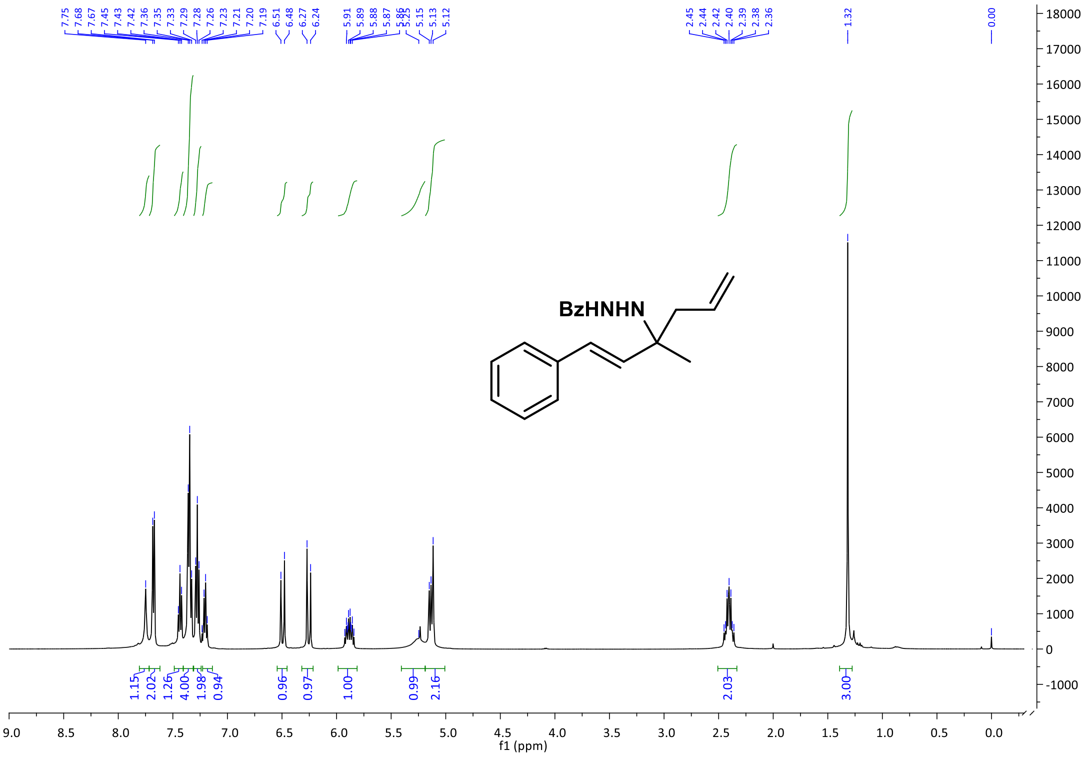


**Supplementary Figure 30.** $^{1}$H NMR spectrum (**17a**)

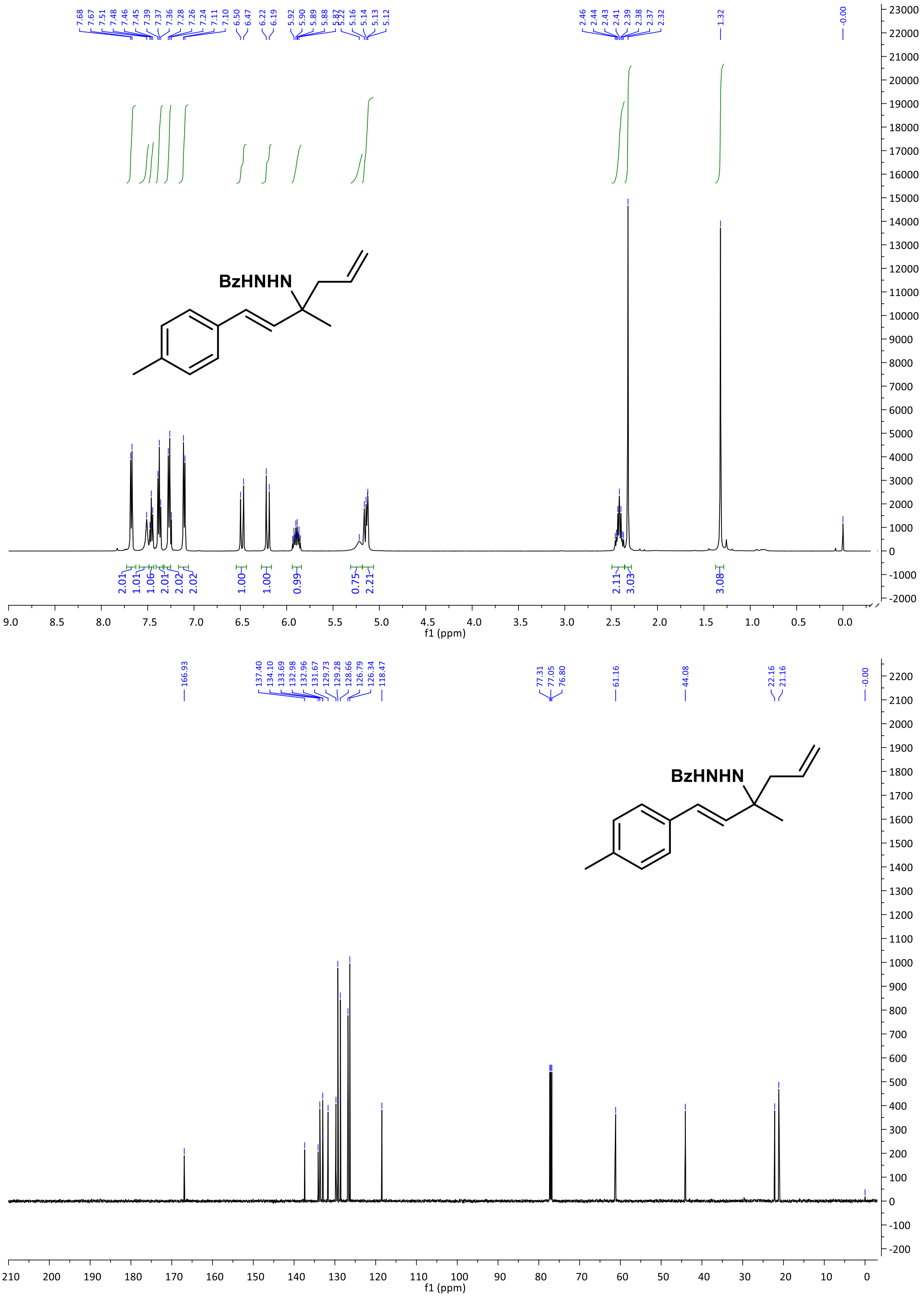


**Supplementary Figure 31.** $^{1}$H and $^{13}$C NMR spectra (**17b**)

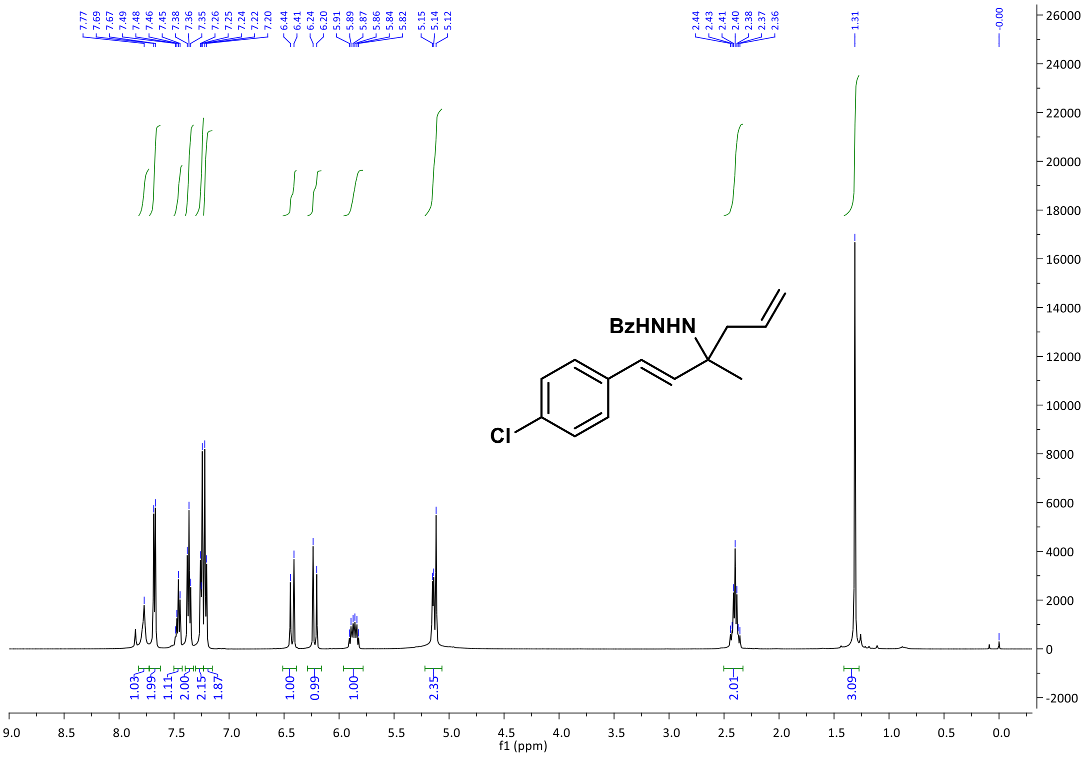

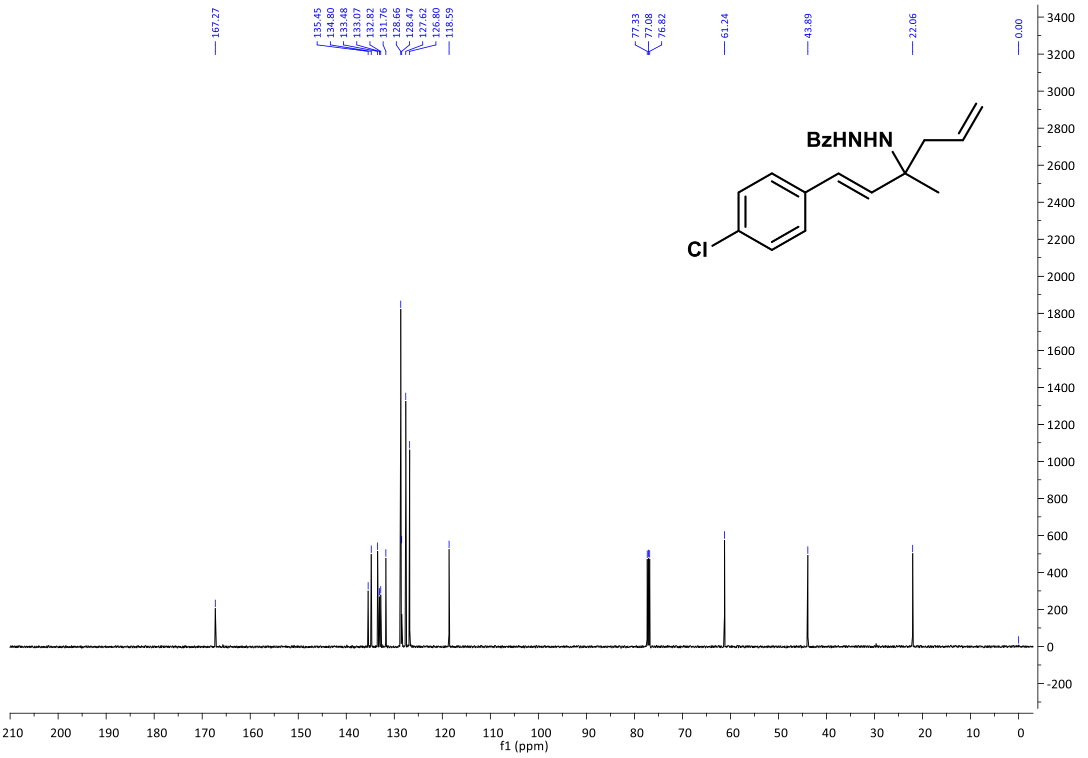

**Supplementary Figure 32.** $^{1}H$ and $^{13}C$ NMR spectra (**17c**)

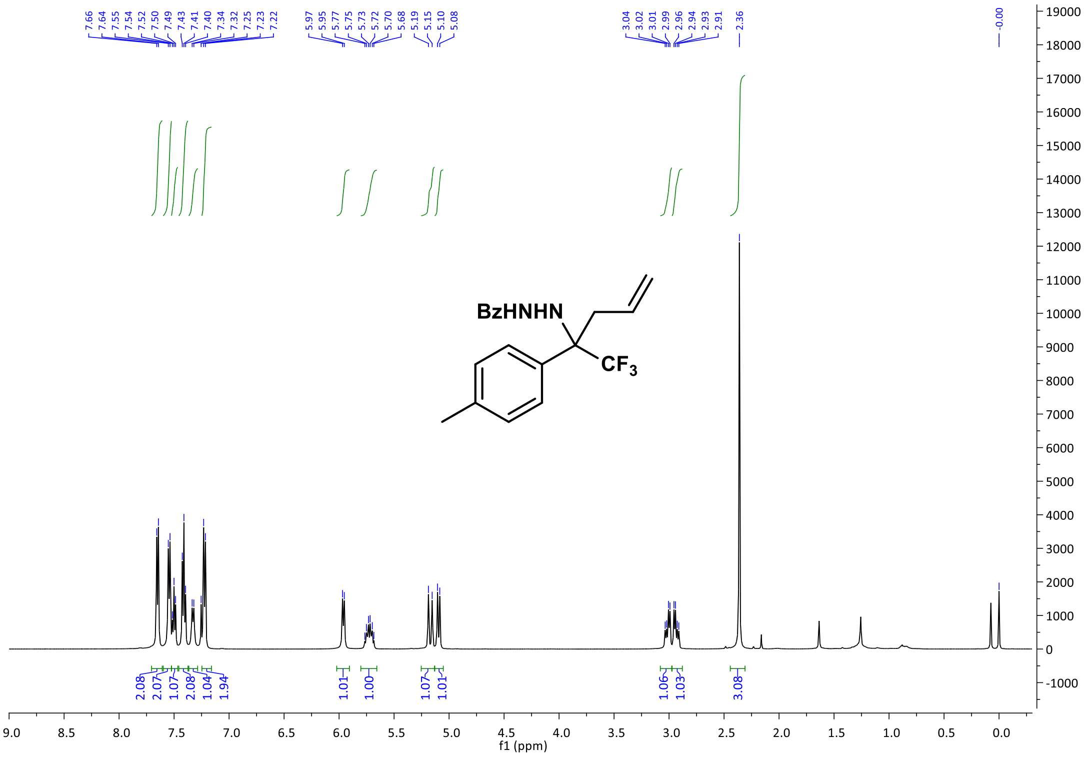


**Supplementary Figure 33.** $^{1}$H NMR spectrum (**19a**)

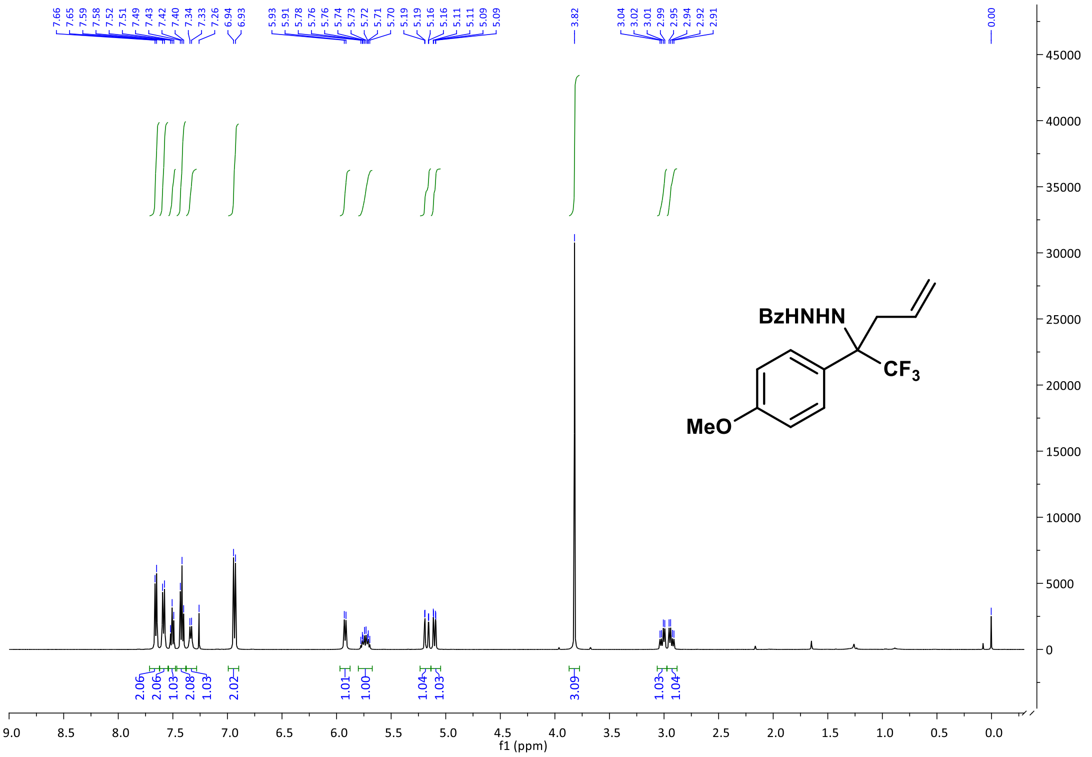


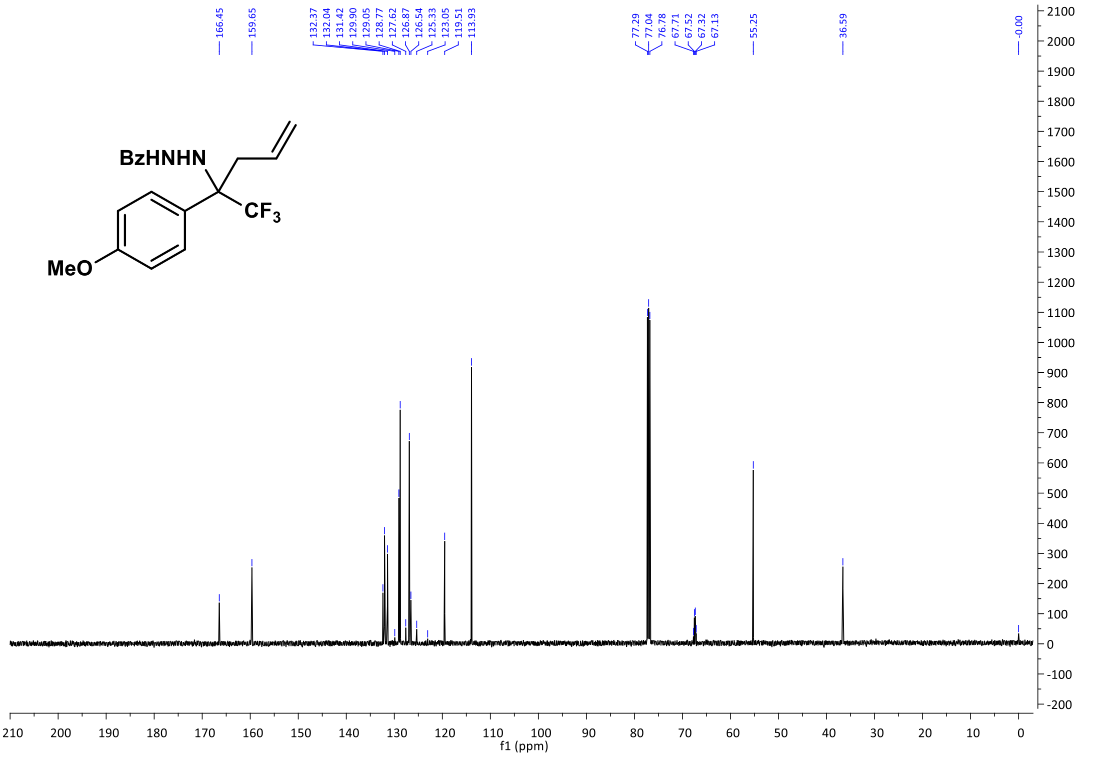


**Supplementary Figure 34.** $^{1}$H and $^{13}$C NMR spectra (**19b**)

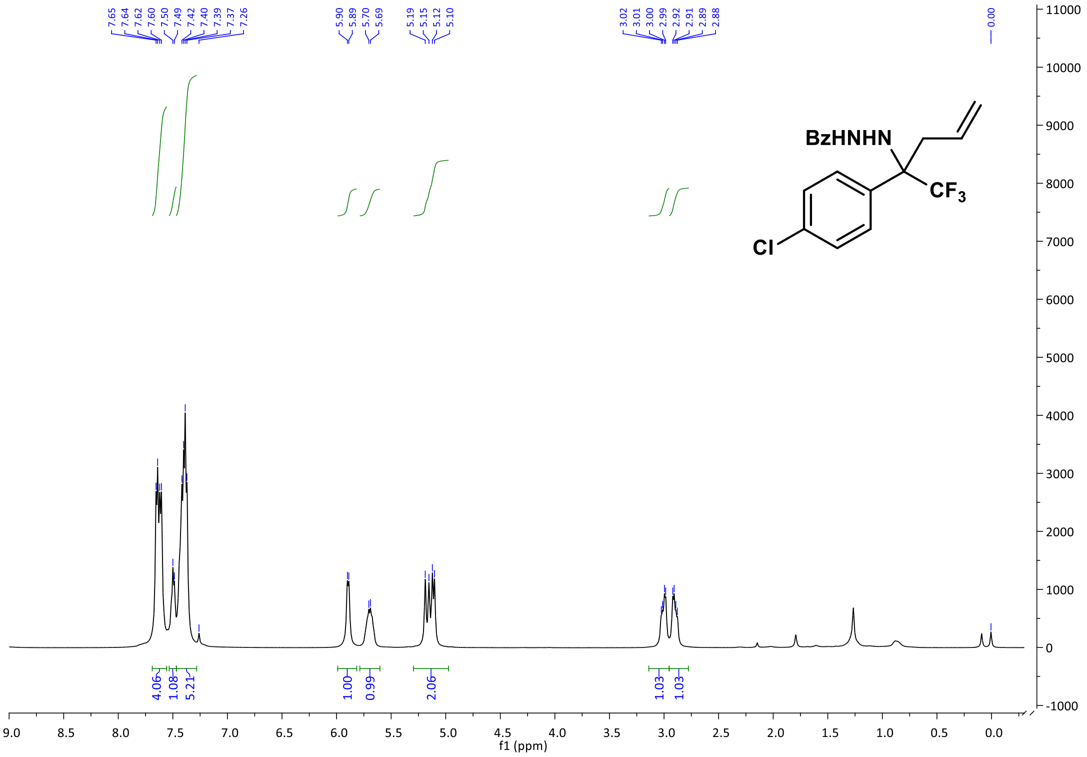


**Supplementary Figure 35.** $^1$H NMR spectrum (**19c**)

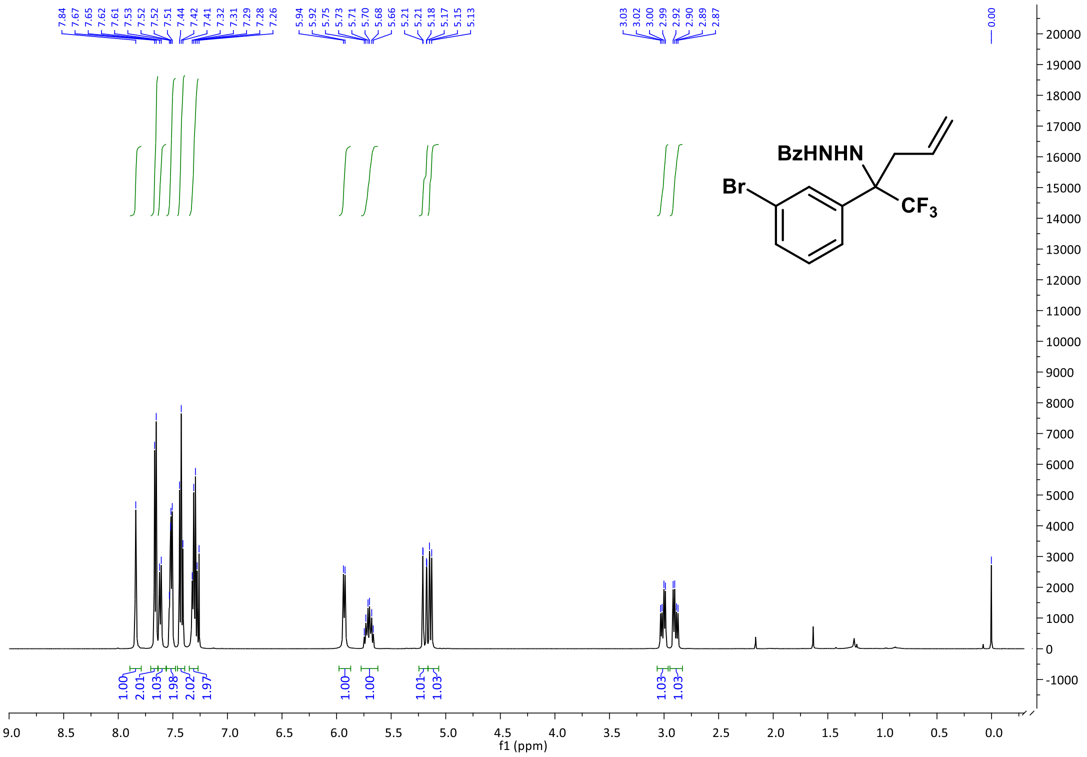

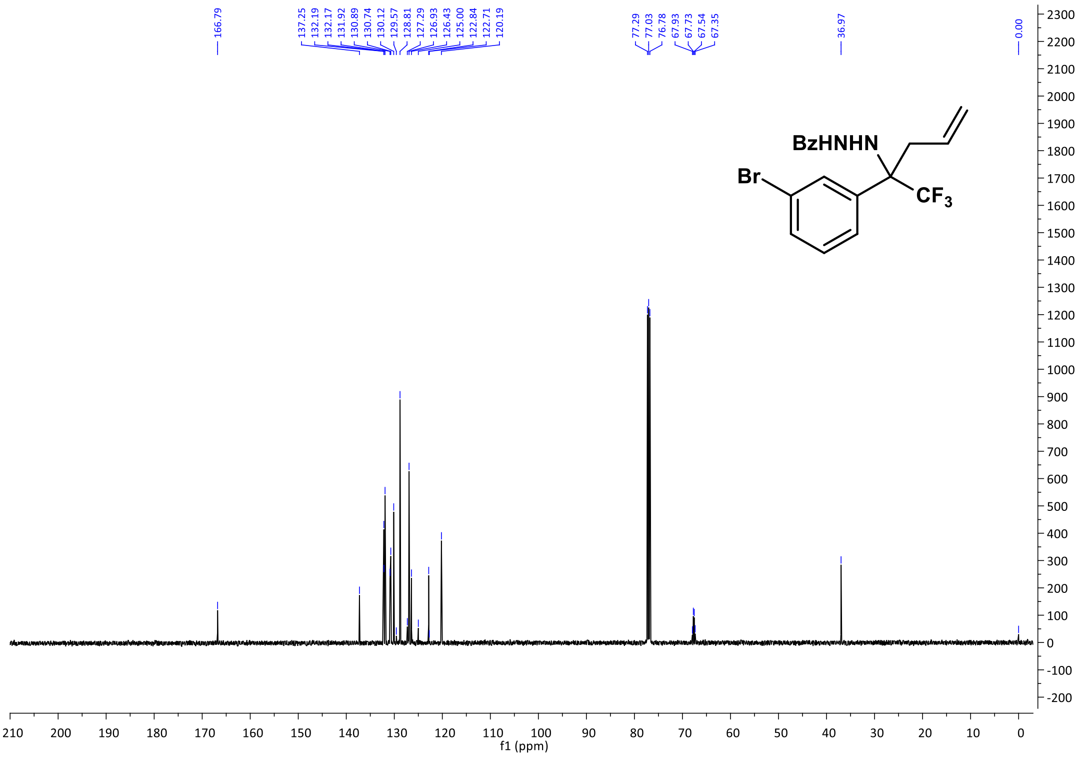

**Supplementary Figure 36.** $^{1}H$ and $^{13}C$ NMR spectra (**19d**)

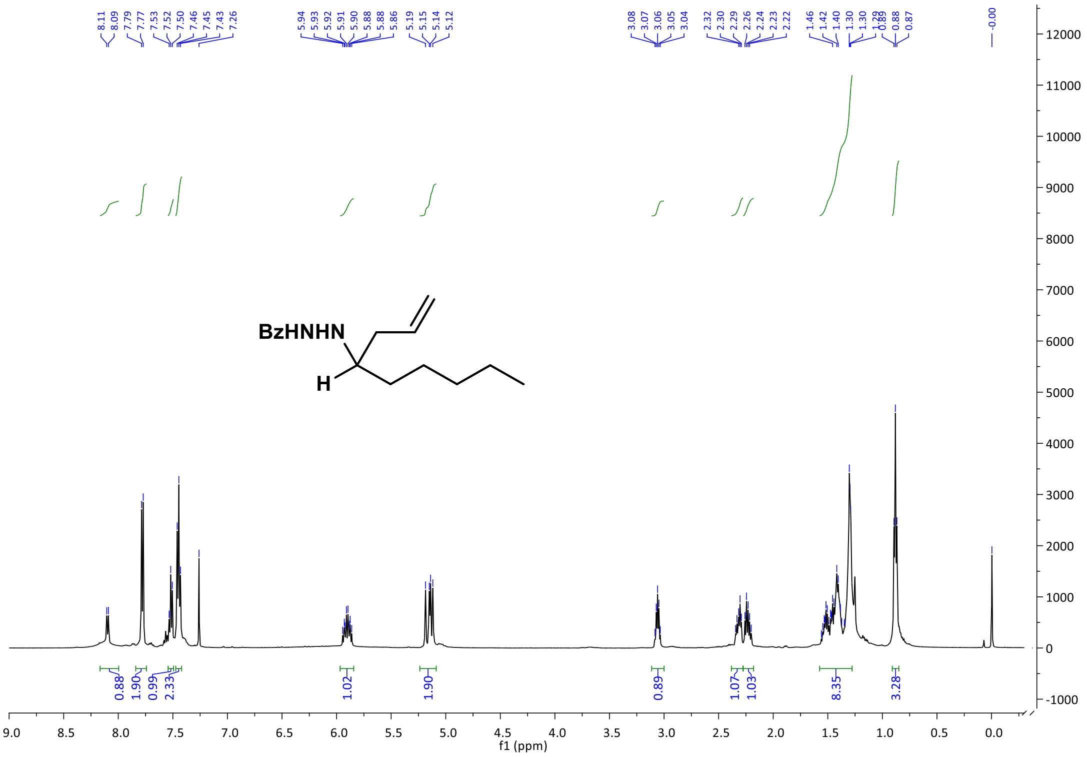


**Supplementary Figure 37.** $^{1}$H NMR spectrum (**21a**)

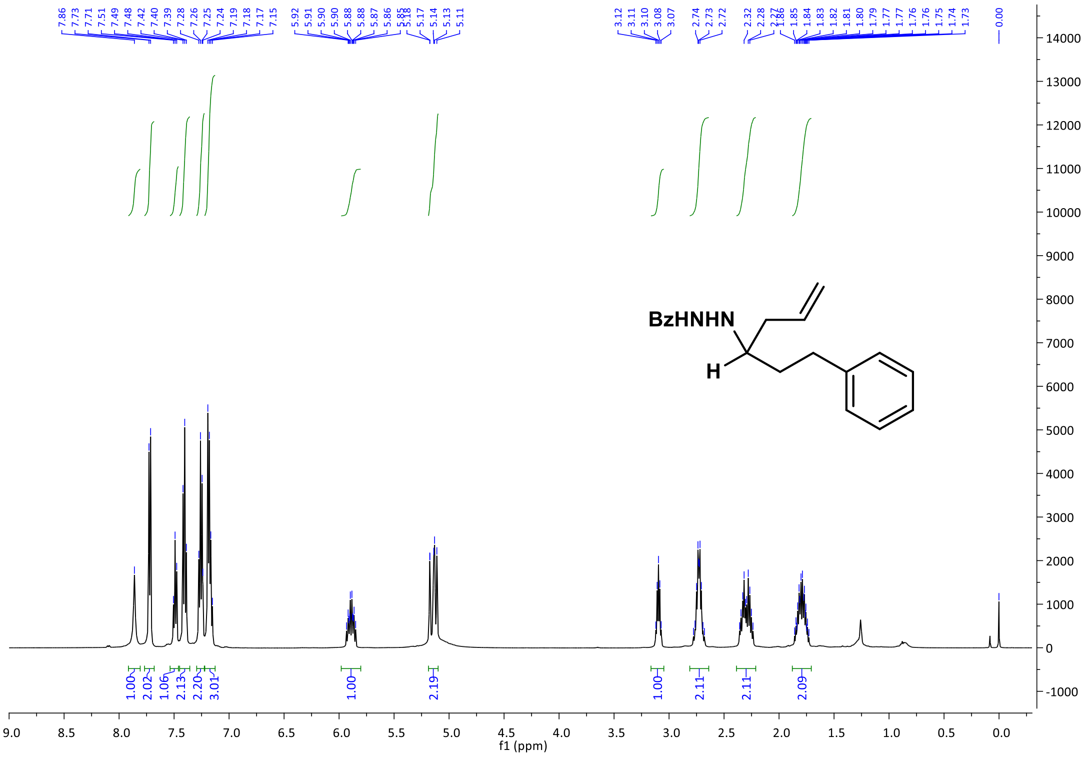

**Supplementary Figure 38.** $^1$H NMR spectrum (**21b**)

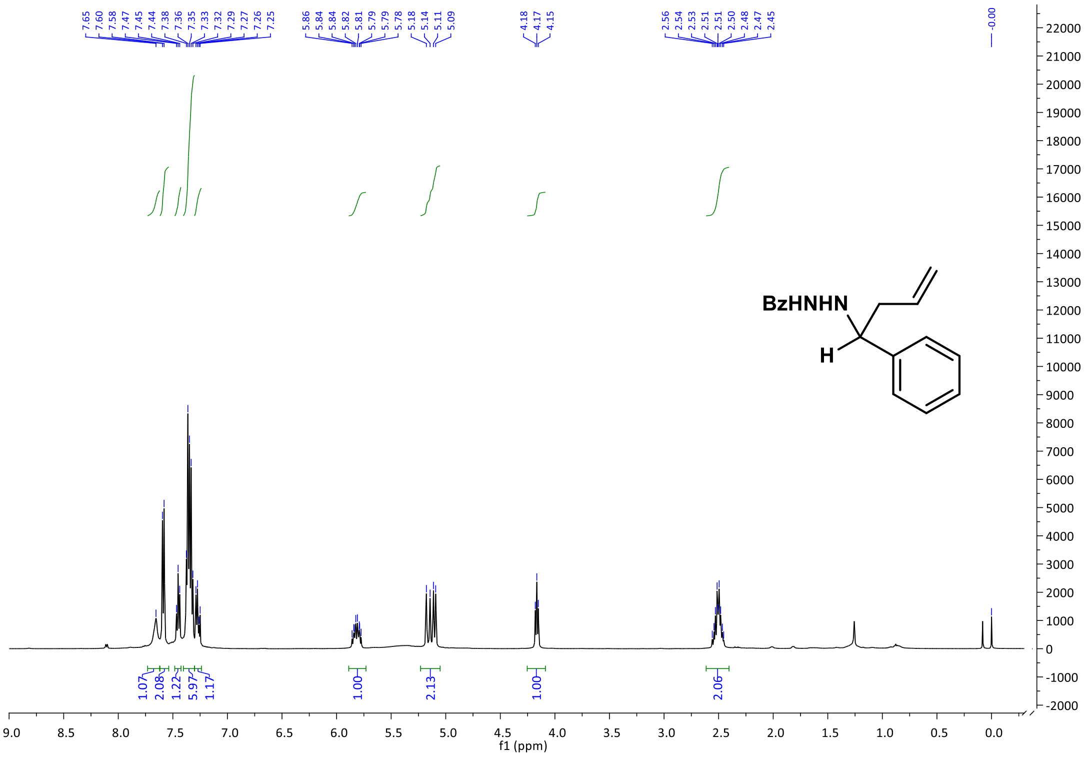

**Supplementary Figure 39.** $^1$H NMR spectrum (**21c**)

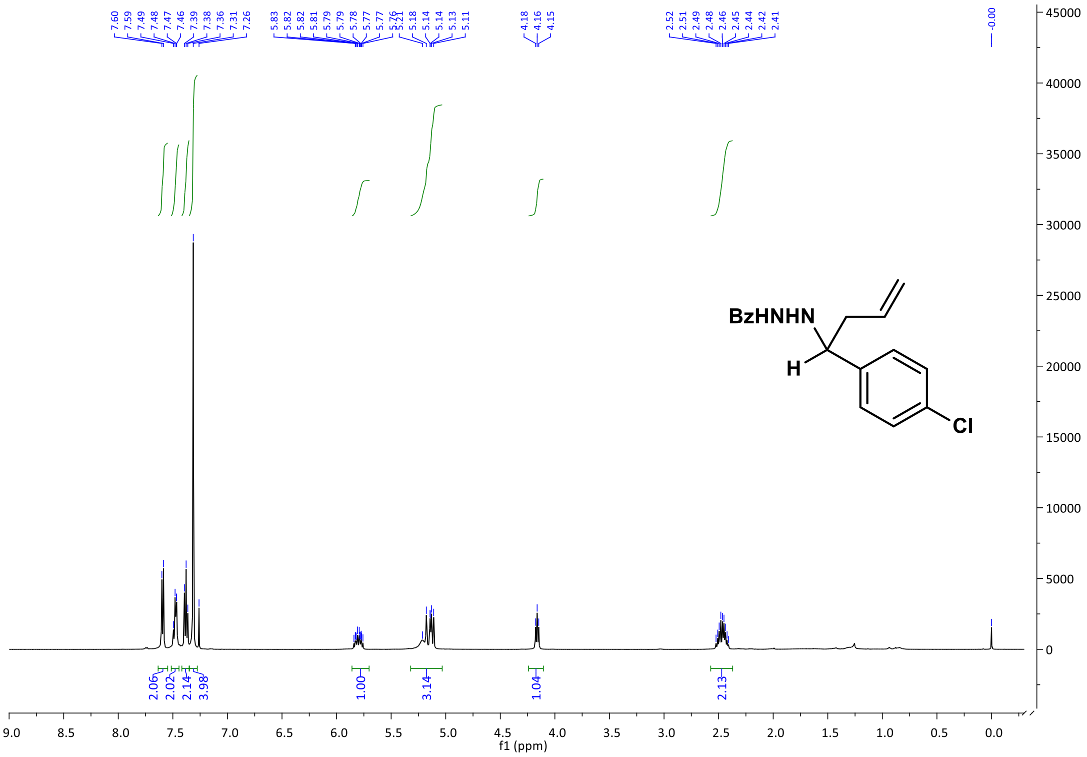

**Supplementary Figure 40.** $^{1}$H NMR spectrum (**21d**)

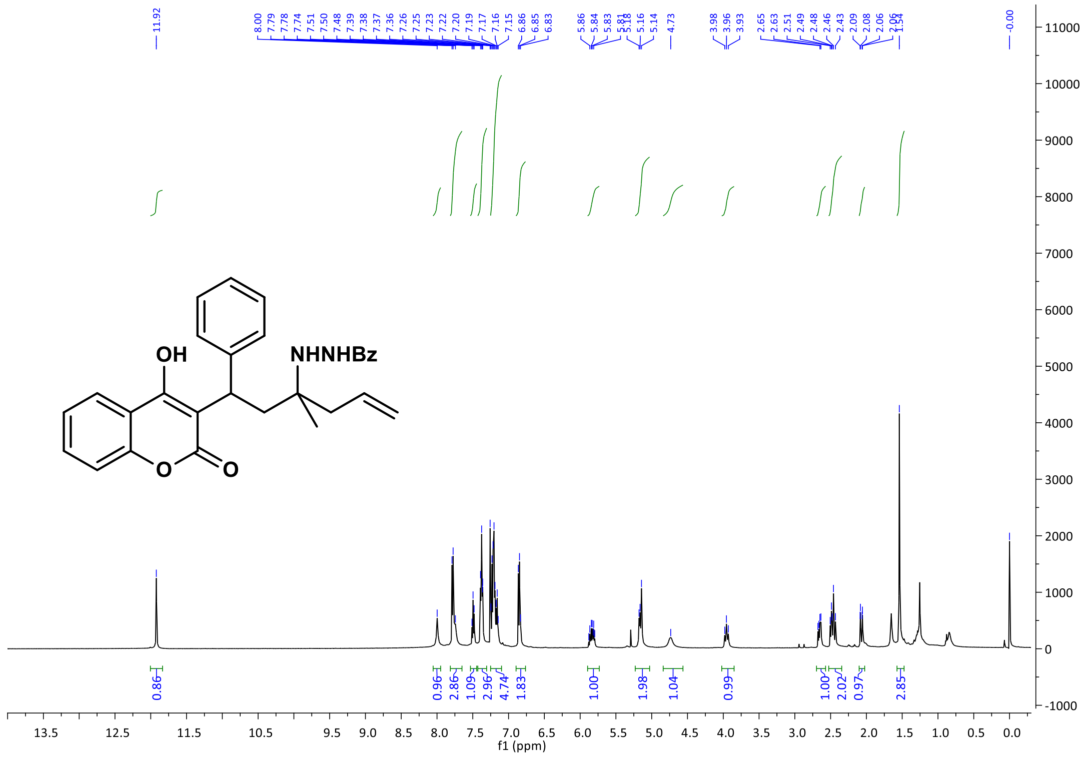

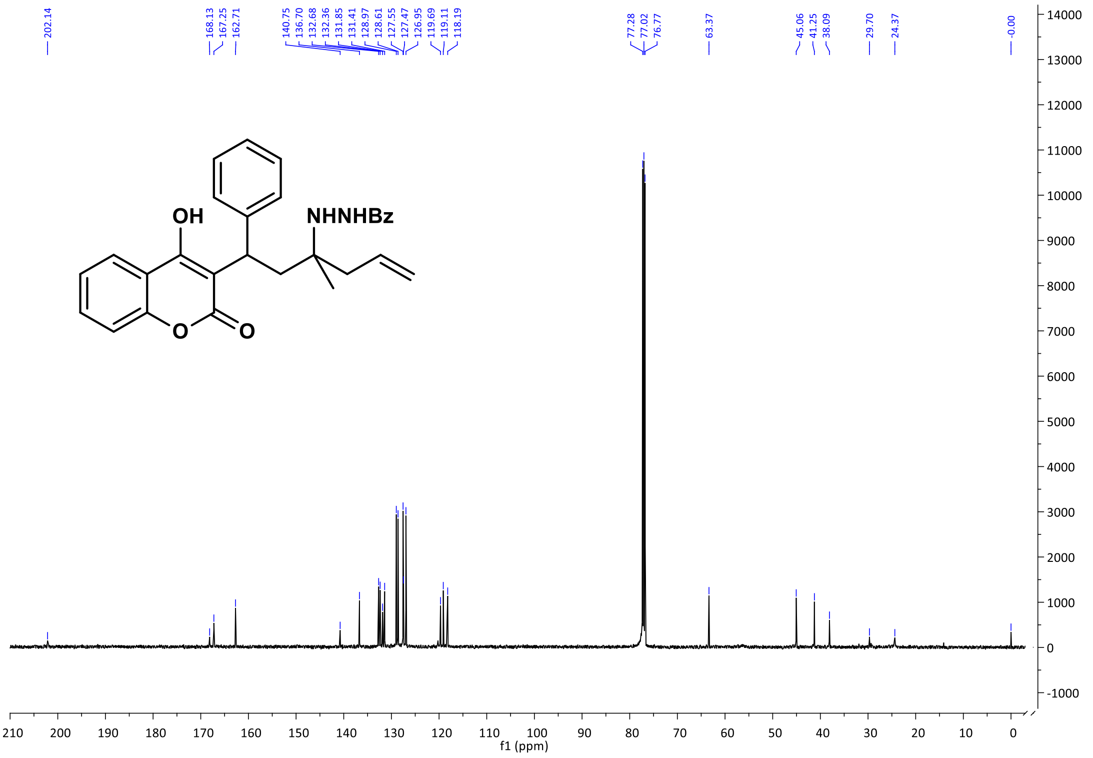

**Supplementary Figure 41.** $^{1}$H and $^{13}$C NMR spectra (**23a**)

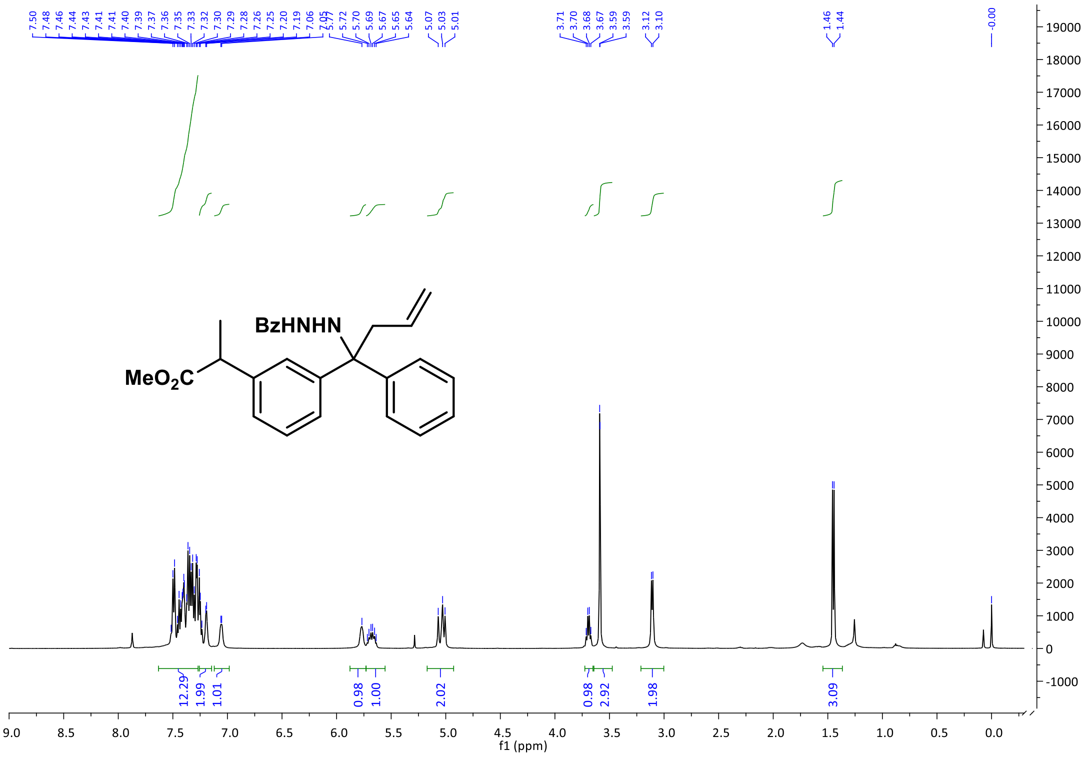


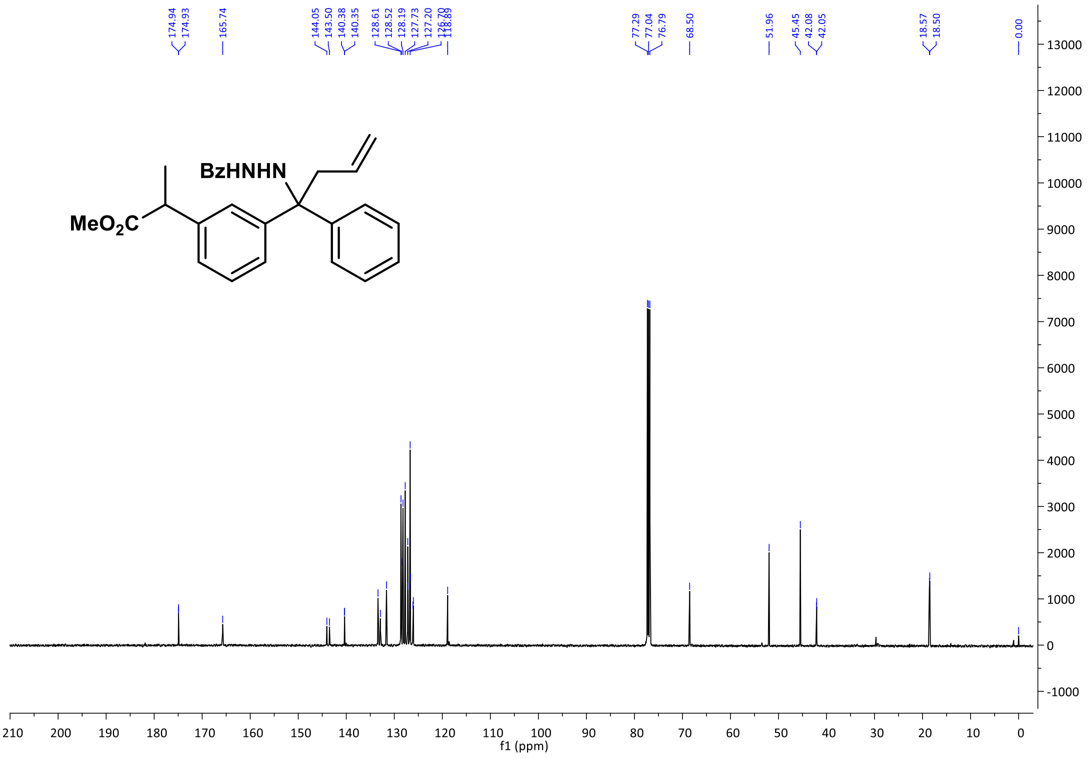


**Supplementary Figure 42.** $^{1}H$ and $^{13}C$ NMR spectra (**23b**)

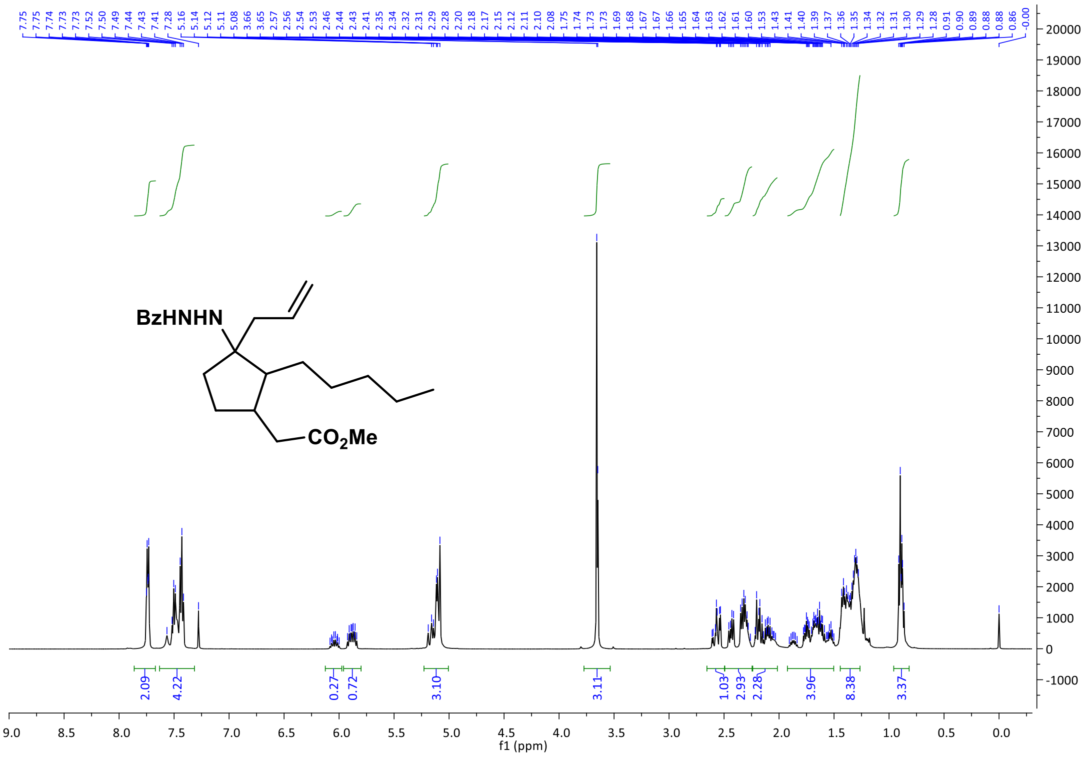

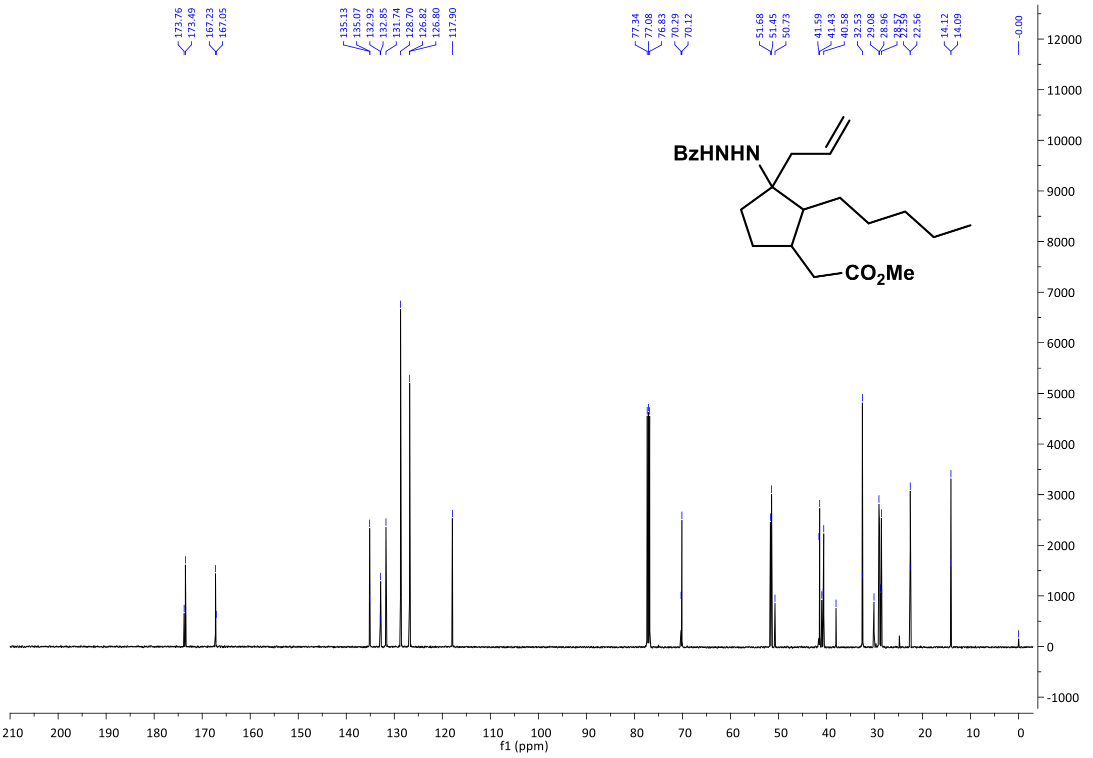

**Supplementary Figure 43.** $^{1}H$ and $^{13}C$ NMR spectra (**23c**)

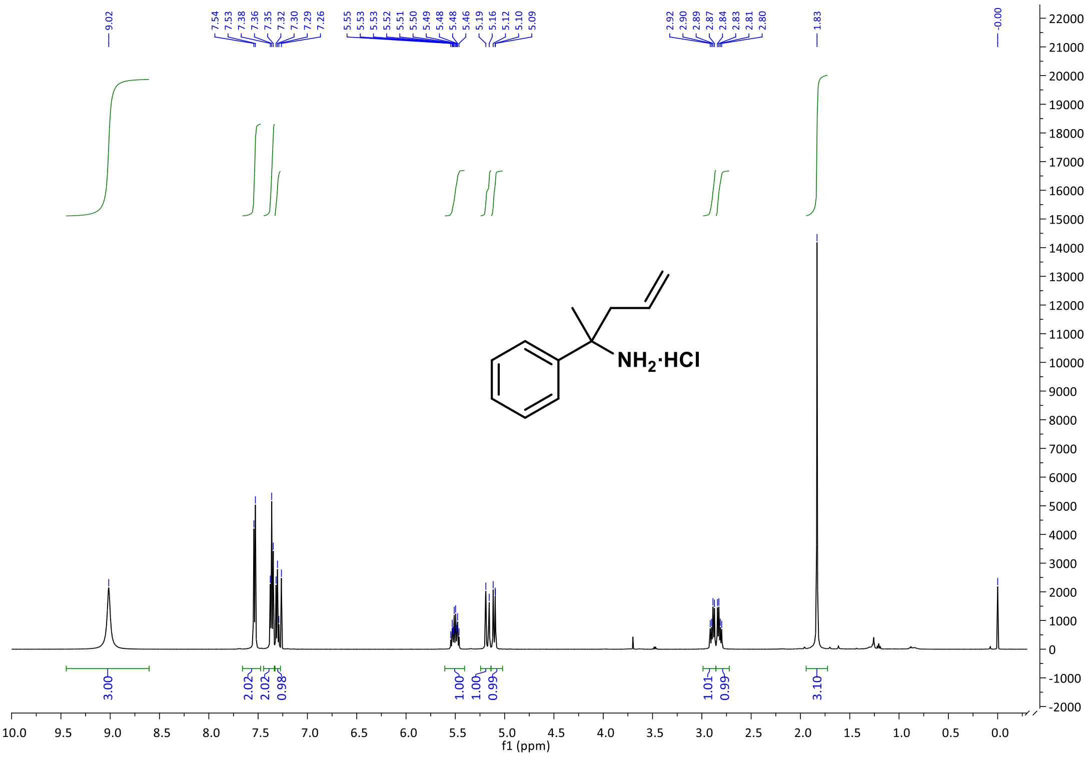

**Supplementary Figure 44.** $^{1}$H NMR spectrum (**25**)

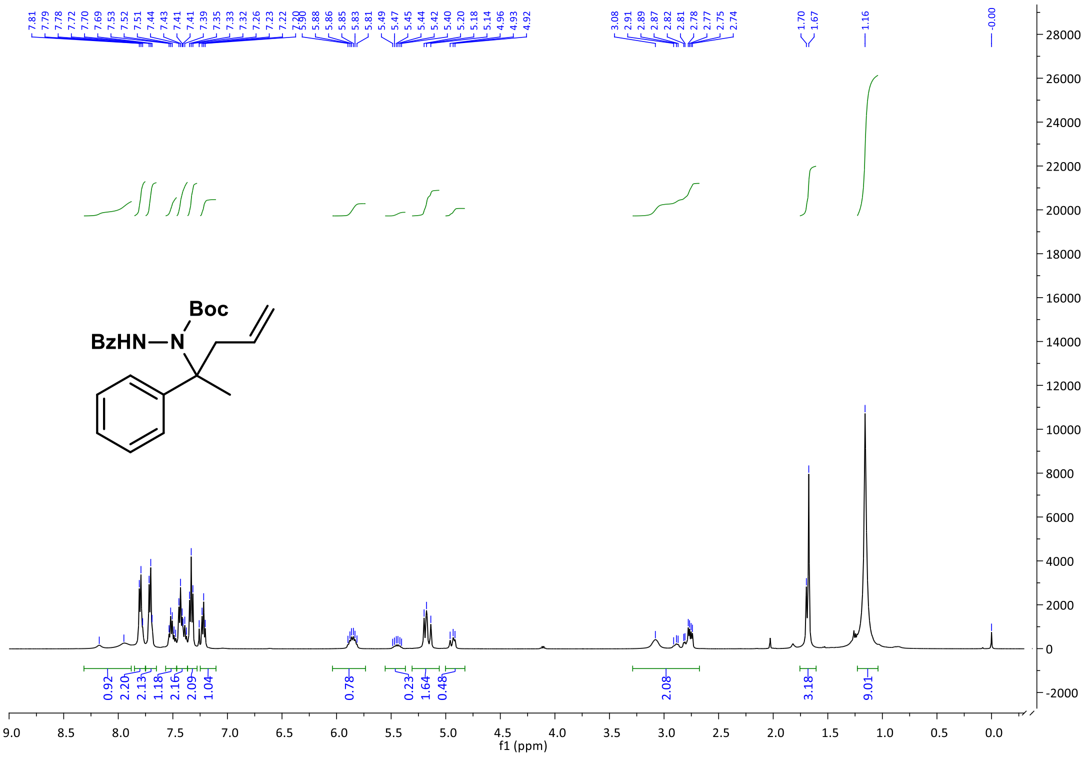

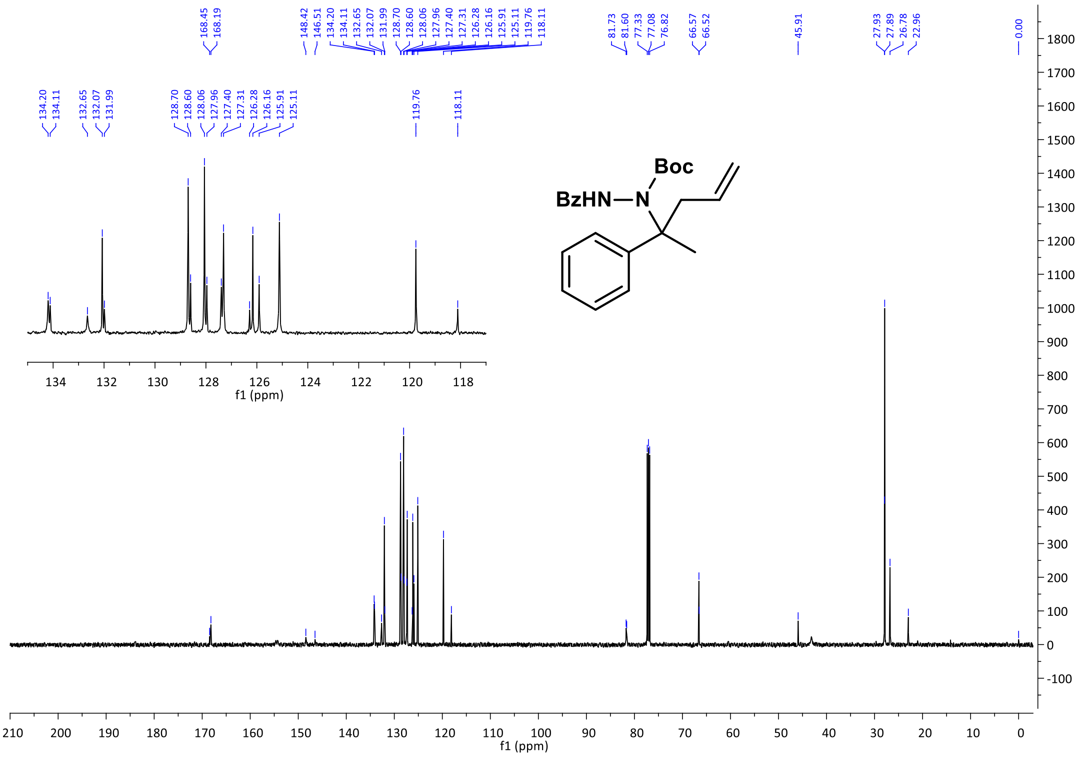

**Supplementary Figure 45.** $^{1}$H and $^{13}$C NMR spectra (**26**)

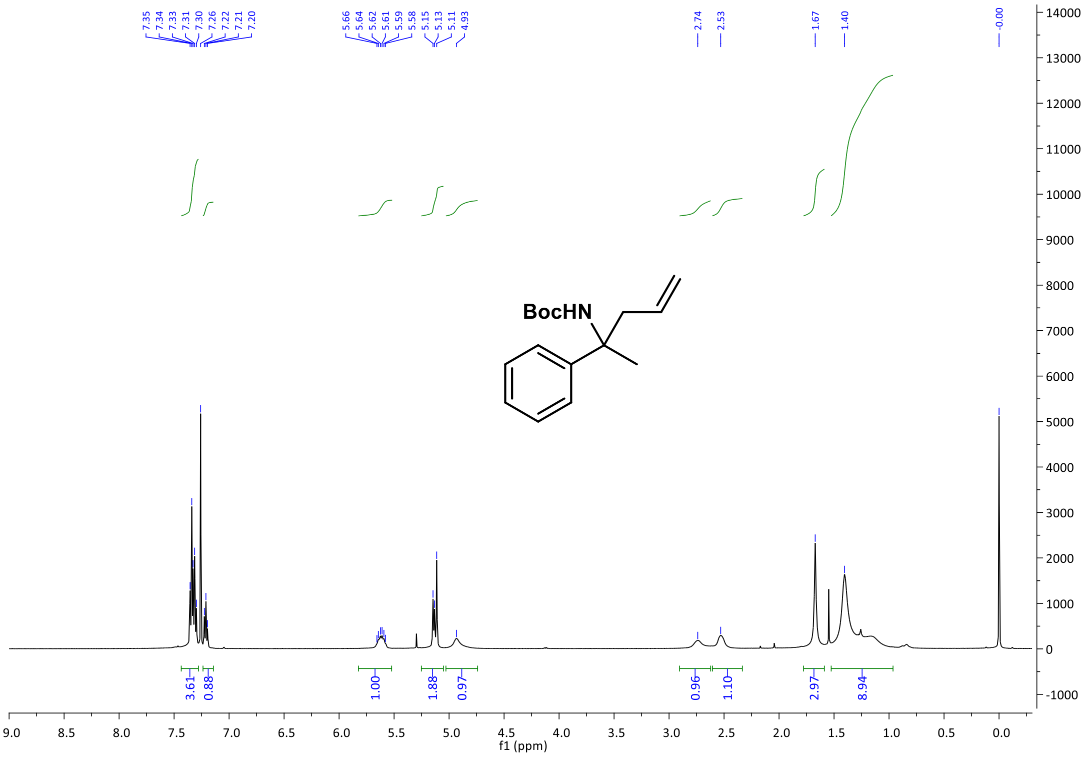


**Supplementary Figure 46.** $^{1}$H NMR spectrum (**27**)

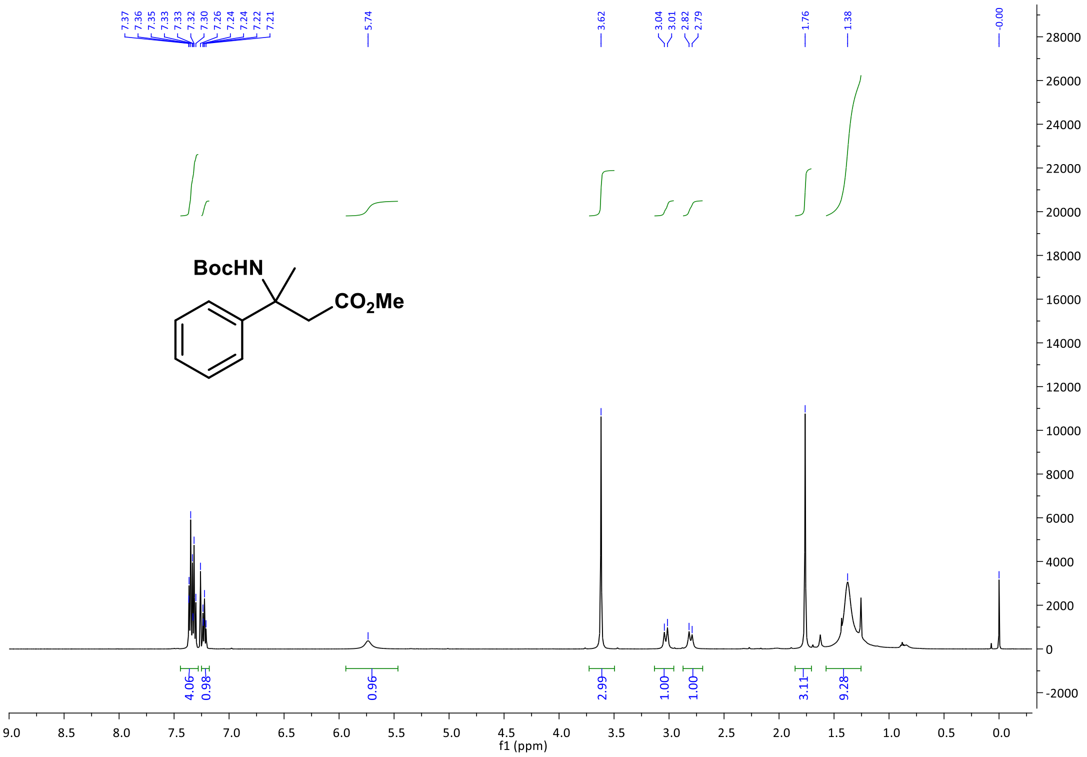


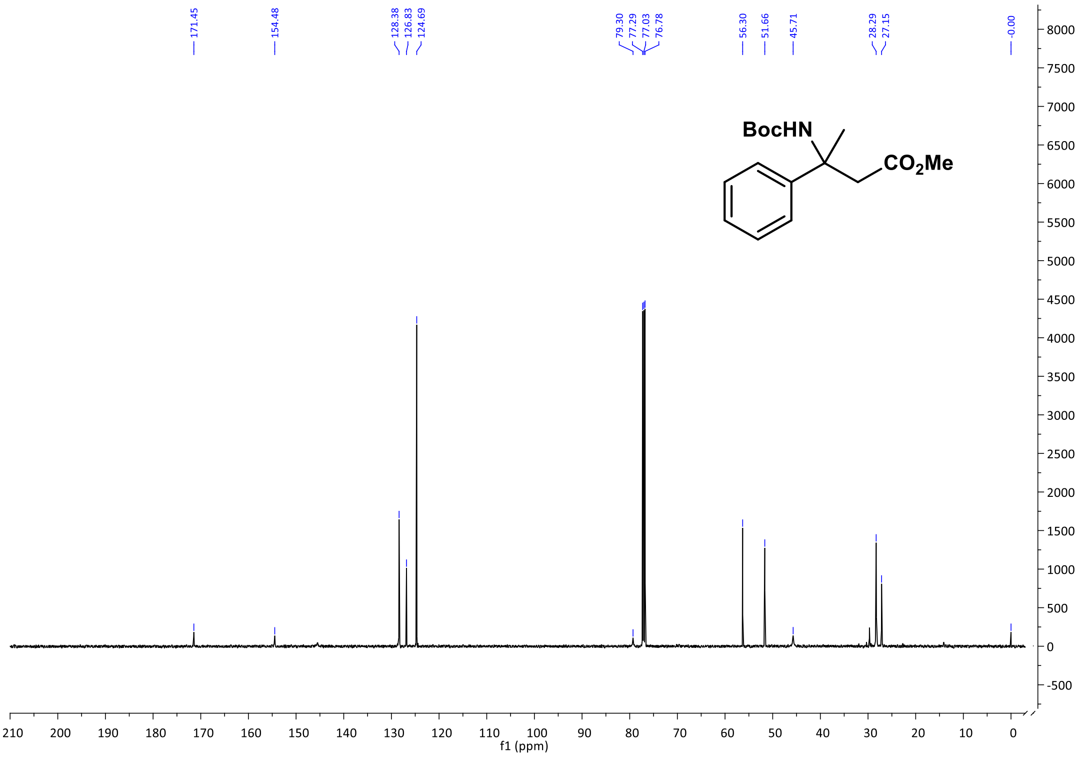


**Supplementary Figure 47.** $^{1}$H and $^{13}$C NMR spectra (**29**)

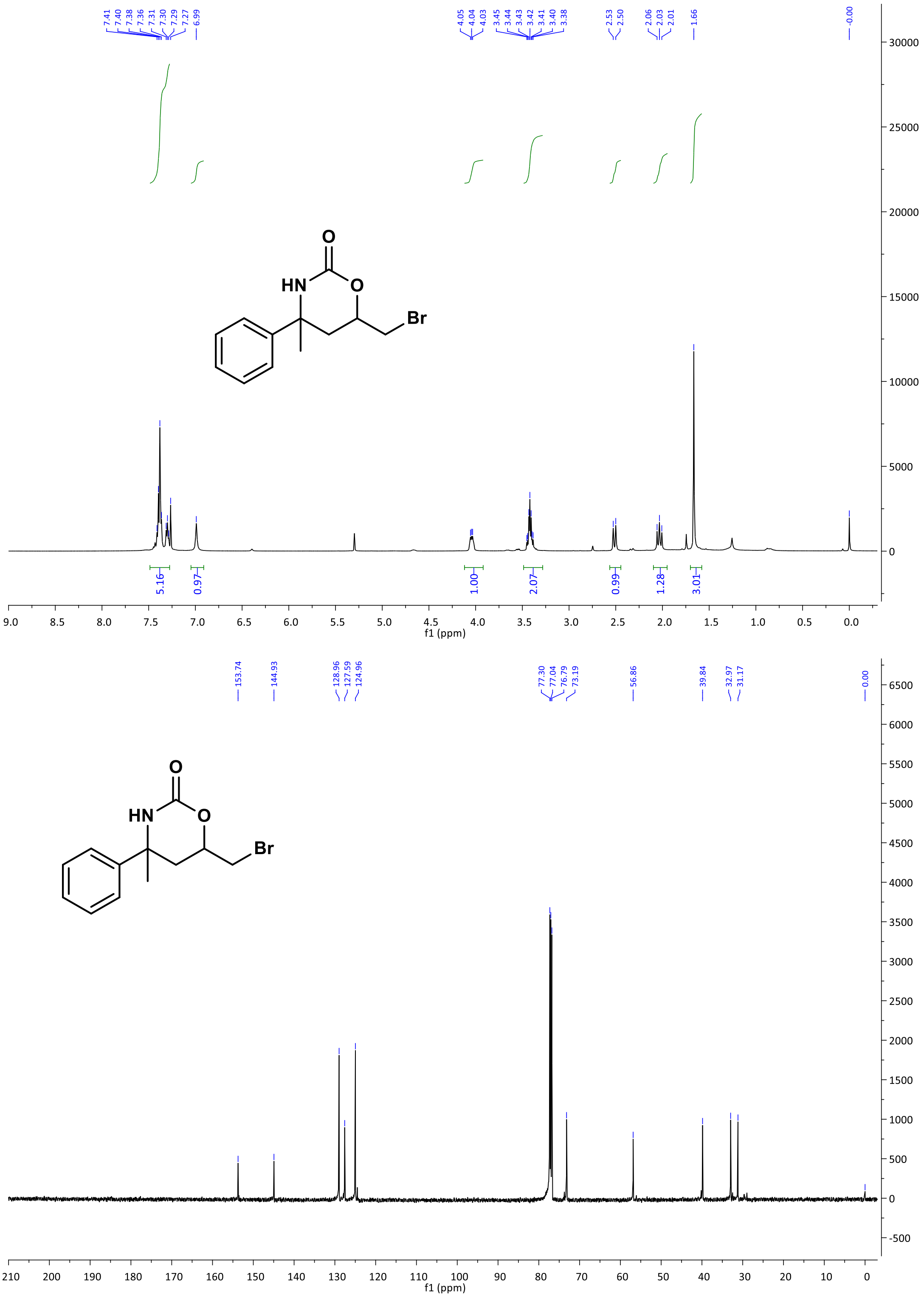


**Supplementary Figure 48.** $^{1}H$ and $^{13}C$ NMR spectra (**30**)

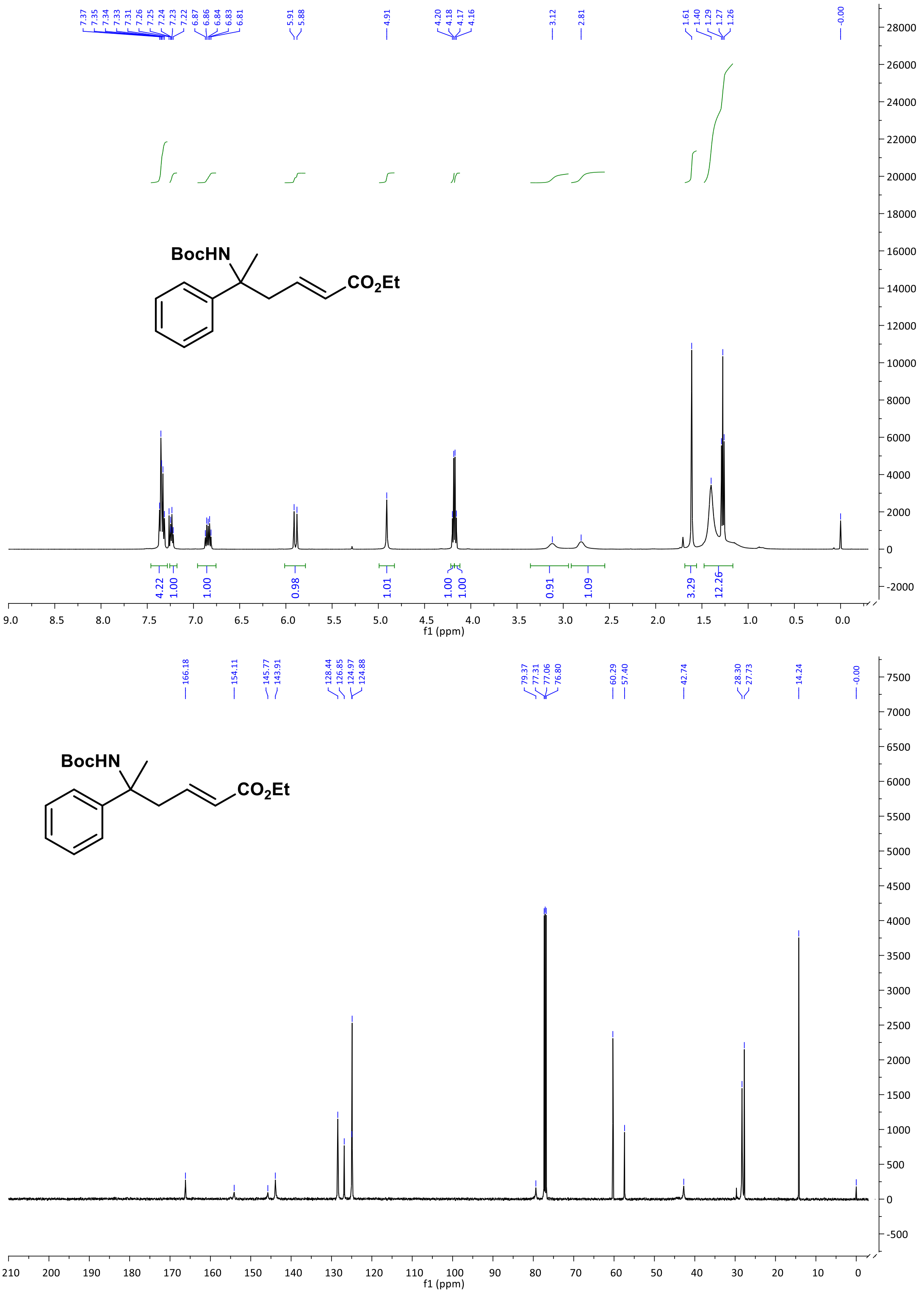


**Supplementary Figure 49.** $^{1}$H and $^{13}$C NMR spectra (**31**)

## 6. Computational Studies on the Mechanistic Investigation

§ Note: For mechanistic investigation, computational studies were systemized by using Spartan '14 (ver. 1.1.4). Geometry minimization was refined by molecular mechanics (MM; MM/MMFF). Energy was calculated by density functional theory (DFT; DFT/B3LYP/6-31G*/PhMe). Analytical result was obtained in a.u. unit, thus converted to kcal/mol unit for comparison (1 a.u. = 2625.5 kJ/mol = 627.51 kcal/mol).

*** SQA-III**

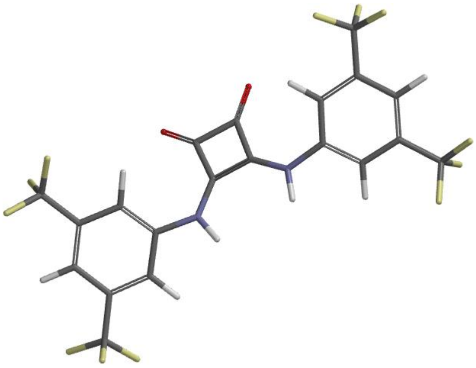

***Summary***
Job type: Energy
Method: B3LYP (with TOLUENE Solvation added)
Basis set: 6-31G*
Charge: neutral (singlet)
Energy: -2224.988789 hartrees
***Output***
Job type: Single point
Method: RB3LYP
Basis set: 6-31G(D)
Number of shells: 160
Number of basis functions: 556
Multiplicity: 1
SCF model: A restricted hybrid HF-DFT SCF calculation will be performed using Pulay DIIS + Geometric Direct Minimization
Solvation: toluene [SM8]
Free Energy of Solvation: -67.7088045 kJ/mol
SCF total energy: -2224.9887892 hartrees

*** *p*TsOH**

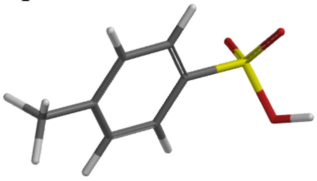

***Summary***
Job type: Energy
Method: B3LYP (with: TOLUENE Solvation added)
Basis set: 6-31G*
Charge: neutral (singlet)
Energy: -895.368506 hartrees
***Output***
Job type: Single point
Method: RB3LYP
Basis set: 6-31G(D)
Number of shells: 61
Number of basis functions: 185
Multiplicity: 1
SCF model: A restricted hybrid HF-DFT SCF calculation will be performed using Pulay DIIS + Geometric Direct minimization
Solvation: toluene [SM8]
Free Energy of Solvation: -40.1962555 kJ/mol
SCF total energy: -895.3685059 hartrees

*** DBSA**

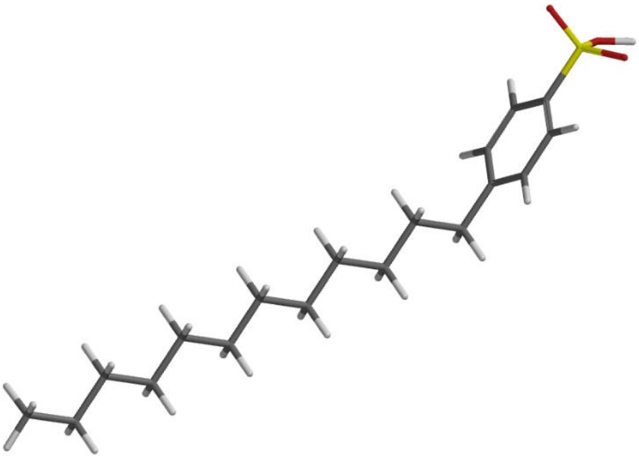

***Summary***
Job type: Energy
Method: B3LYP (with: TOLUENE Solvation added)
Basis set: 6-31G*
Charge: neutral (singlet)
Energy: -1327.827110 hartrees
***Output***
Job type: Single point
Method: RB3LYP
Basis set: 6-31G(D)
Number of shells: 149
Number of basis functions: 394
Multiplicity: 1
SCF model: A restricted hybrid HF-DFT SCF calculation will be performed using Pulay DIIS + Geometric Direct Minimization
Solvation: toluene [SM8]
Free Energy of Solvation: -67.6197277 kJ/mol
SCF total energy: -1327.8271102 hartrees

*** SQA-III + *p*TsOH**

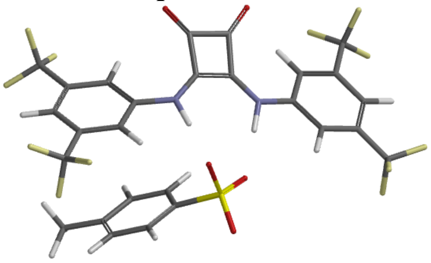

***Summary***
Job type: Energy
Method: B3LYP (with: TOLUENE Solvation added)
Basis set: 6-31G*
Charge: -1
Energy: -3119.918695 hartrees
HB Count: 2

*** SQA-III + DBSA**

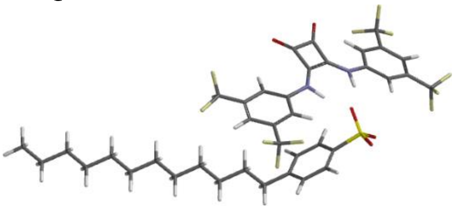

***Summary***
Job type: Energy
Method: B3LYP (with: TOLUENE Solvation added)
Basis set: 6-31G*
Charge: -1
Energy: -3552.380732 hartrees
HB Count: 2

**Supplementary Table 4.** Complex energy comparison

| | **SQA-III + *p*TsOH** | **SQA-III + DBSA** |
|---|---|---|
| **a.u.** | -3119.91870 | -3552.38073 |
| **kJ/mol** | -8191346.55 | -9326775.61 |
| **kcal/mol** | -1957780.18 | -2229154.43 |
| **$\Delta E_{(complex-catalyst)}$ (kcal/mol)** | -1395927.49 | -1395929.64 |
| **$\Delta E_{(stabilization)}$ (kcal/mol)** | | -2.15 |

## 7. Supplementary References


1. Sammet, K., Gastl, C., Baro, A., Laschat, S., Fischer, P. & Fettig, I. Enders's SAMP-Hydrazone as Traceless Auxiliary in the Asymmetric 1,4-Addition of Cuprates to Enones. *Adv. Synth. Catal.* **352**, 2281–2290 (2010).
2. Kerr, W. J., Mudd, R. J., Paterson, L. C. & Brown, J. A. Iridium(I)-Catalyzed Regioselective C–H Activation and Hydrogen-Isotope Exchange of Non-aromatic Unsaturated Functionality. *Chem. Eur. J.* **20**, 14604–14607 (2014).
3. Liu, J., Chen, C., Li, Z., Wu, W., Zhi, X., Zhang, Q., Wu, H., Wang, X., Cui, S. & Guo, K. A squaramide and tertiary amine: an excellent hydrogen-bonding pair organocatalyst for living polymerization. *Polym. Chem.* **6**, 3754–3757 (2015).
4. Rombola, M., Sumaria, C. S., Montgomery, T. D. & Rawal, V. H. Development of Chiral, Bifunctional Thiosquaramides: Enantioselective Michael Additions of Barbituric Acids to Nitroalkenes. *J. Am. Chem. Soc.* **139**, 5297–5300 (2017).
5. Sandler, I., Larik, F. A., Mallo, N., Beves, J. E. & Ho, J. Anion Binding Affinity: Acidity versus Conformational Effects. *J. Org. Chem.* **85**, 8074–8084 (2020).
6. Lee, B. S. & Jang, D. O. A Mild and Efficient Three-Component Synthesis of Secondary and Tertiary Homoallylic Hydrazides. *Eur. J. Org. Chem.* **2013**, 3123 – 3130 (2013).
7. Lu, A., Huang, D., Wang, K.-H., Su, Y., Ma, J., Xu, Y. & Hu, Y. Tin-Mediated One-Pot Synthesis of α,α-Disubstituted Homoallylic Hydrazides from Ketones, Acylhydrazines and Allyl Bromide. *Synthesis* **48**, 293–301 (2016).
8. Berger, R., Duff, K. & Leighton, J. L. Enantioselective Allylation of Ketone-Derived Benzoylhydrazones: Practical Synthesis of Tertiary Carbinamines. *J. Am. Chem. Soc.* **126**, 5686–5687 (2004).
9. Wang, K., Wang, Y., Yin, X., Peng, X., Huang, D., Su, Y. & Hu, Y. Tin-Promoted One-Pot Synthesis of Aryl/Trifluoromethyl Group Substituted Homoallylic *N*-Acylhydrazines. *Chin J. Org. Chem.* **37**, 1764–1773 (2017).
10. Hirabayashi, R., Ogawa, C., Sugiura, M. & Kobayashi, S. Highly Stereoselective Synthesis of Homoallylic Amines Based on Addition of Allyltrichlorosilanes to Benzoylhydrazones. *J. Am. Chem. Soc.* **123**, 9493–9499 (2001).
11. Ogawa, C., Konishi, H., Sugiura, M. & Kobayashi, S. Phosphine oxides as efficient neutral coordinate-organocatalysts for stereoselective allylation of *N*-acylhydrazones. *Org. Biomol. Chem.* **2**, 446–448 (2004).
12. Schneider, U., Chen, I-H. & Kobayashi, S. Development of General Catalytic Allylation of Acylhydrazones with Pinacolyl Allylboronate Using an Indium(I) Catalyst. *Org. Lett.* **10**, 737–740 (2008).
13. Park, Y. S., Boys, M. L. & Beak, P. (–)-Sparteine-Mediated α-Lithiation of *N*-Boc-*N*-(*p*-methoxyphenyl)benzylamine: Enantioselective Syntheses of (*S*) and (*R*) Mono- and Disubstituted *N*-Boc-benzylamines. *J. Am. Chem. Soc.* **118**, 3757–3758 (1996).
14. Kuznetsov, N. Y., Tikhov, R. M., Strelkova, T. V. & Bubnov, Y. N. Adducts of Triallylborane with Ammonia and Aliphatic Amines as Stoichiometric Allylating Agents for Aminoallylation Reaction of Carbonyl Compounds. *Org. Lett.* **20**, 3549–3552 (2018).
